# A Review of Fungal Protoilludane Sesquiterpenoid Natural Products

**DOI:** 10.3390/antibiotics9120928

**Published:** 2020-12-19

**Authors:** Melissa M. Cadelis, Brent R. Copp, Siouxsie Wiles

**Affiliations:** 1 School of Chemical Sciences, University of Auckland, 23 Symonds Street, Auckland 1010, New Zealand; b.copp@auckland.ac.nz; 2 Bioluminescent Superbugs Lab, School of Medical Sciences, University of Auckland, 85 Park Road, Grafton, Auckland 1023, New Zealand; s.wiles@auckland.ac.nz

**Keywords:** sesquiterpenoid, natural products, protoilludanes

## Abstract

Natural products have been a great source for drug leads, due to a vast majority possessing unique chemical structures. Such an example is the protoilludane class of natural products which contain an annulated 5/6/4-ring system and are almost exclusively produced by fungi. They have been reported to possess a diverse range of bioactivities, including antimicrobial, antifungal and cytotoxic properties. In this review, we discuss the isolation, structure elucidation and any reported bioactivities of this compound class, including establishment of stereochemistry and any total syntheses of these natural products. A total of 180 protoilludane natural products, isolated in the last 70 years, from fungi, plant and marine sources are covered, highlighting their structural diversity and potential in drug discovery.

## 1. Introduction

Fungi remain an untapped source of bioactive natural products with an estimate of over 1.5 million species of fungi worldwide, of which only approximately 7% have been chemically investigated [1,2,3,4,5,6]. Over 90% of the recorded species are higher fungi which are considered one of the largest biodiverse resources on Earth. With the development of fungal natural product isolation techniques and a better understanding of biosynthetic pathways, higher fungi have become an abundant source for drug discovery. Ascomycota, the largest phylum of fungi, is one of the two divisions of higher fungi along with Basidiomycota. Both divisions of higher fungi, having undergone evolution to survive harsh and unfavourable environments, are well known producers of an array of structurally diverse natural products that possess a variety of bioactivities. These natural products can be divided into several groups by their chemical structures, one of which are the terpenoids. Representative terpenoid scaffolds in higher fungi include sesquiterpenoids, diterpenoids and triterpenoids which are all derived from common precursors [1,2,3,4,5,6].

**Scheme 1:**
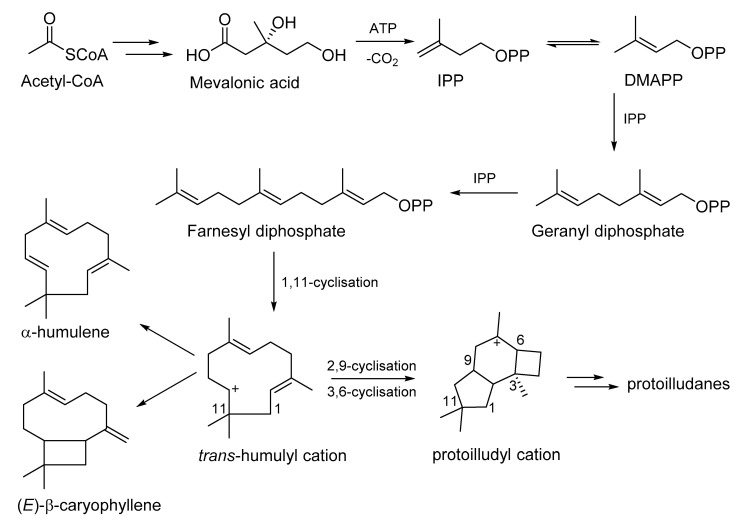
Biosynthesis of sesquiterpenoids from isopentenyl pyrophosphate (IPP) and dimethylallyl pyrophosphate (DMAPP) through the mevalonate pathway.

Fungal terpenoid natural products are derived from isoprene units (C_5_) and are classified by the number of isoprene units in the structure [7,8,9,10,11]. Higher terpenoids such as monoterpenoids (C_10_), sesquiterpenoids (C_15_) and diterpenoids (C_20_) are synthesised from two isoprene intermediates, dimethylallyl pyrophosphate (DMAPP) and isopentenyl pyrophosphate (IPP), in the mevalonate pathway. The mevalonate pathway begins with the phosphorylation of mevalonic acid, derived from acetyl-CoA, by two molecules of adenosine triphosphate (ATP). Subsequent decarboxylation forms IPP, the stereospecific isomerisation of which affords DMAPP (Scheme 1). Loss of diphosphate from DMAPP forms an allylic cation which undergoes nucleophilic attack by IPP to afford geranyl diphosphate. Reaction of geranyl diphosphate with another unit of IPP gives farnesyl diphosphate from which sesquiterpenoids (C_15_) are derived. Repetition of this process results in the formation of terpenoid chains of different length. Linear terpenoids can undergo cyclisation by a number of different terpene cyclases which can generate numerous terpenoid scaffolds [7,8,9,10,11]. 

Cyclisation of farnesyl diphosphate can produce a wide range of natural products, many of which are biosynthesised through a cyclisation pathway involving a humulyl cation [12,13,14,15]. The humulene pathway proceeds through 1,11-cyclisation of 2*E*,6*E*-farnesyl diphosphate to afford the *trans*-humulyl cation which can then undergo rearrangement to produce a number of different classes of sesquiterpenoid carbon skeletons including α-humulene and (*E*)-β-caryophyllene. Of interest is the 2,9 and 3,6-cyclisation product, protoilludyl cation, the precursor to various protoilludane natural products [12,13,14,15].

**Figure 1:**
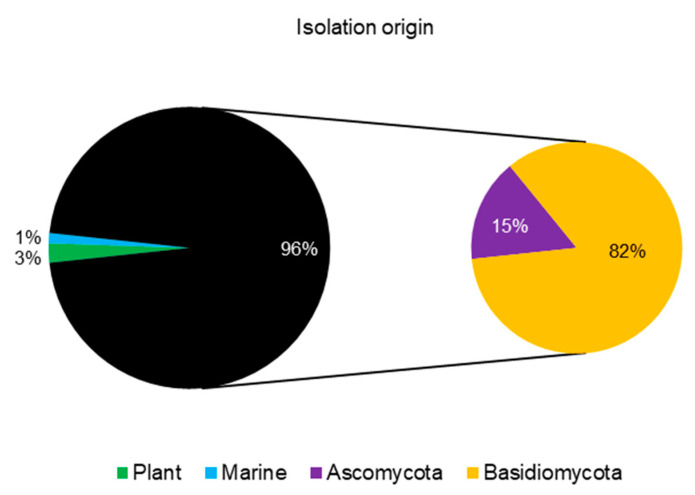
Isolation sources of protoilludane natural products.

**Figure 2:**
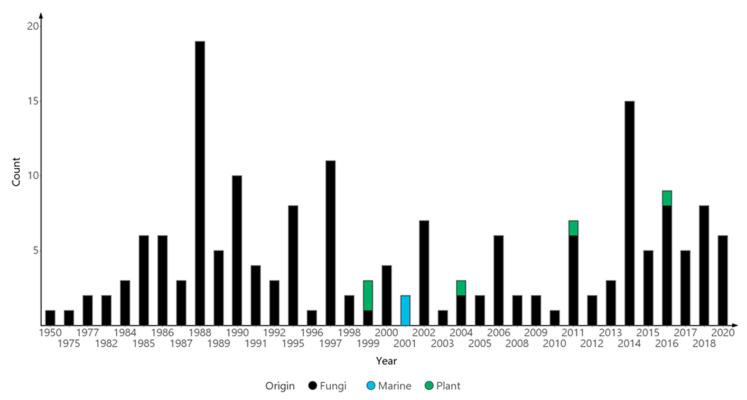
Timeline of the discovery of protoilludane natural products from marine, plant and fungal sources.

Sesquiterpenoid protoilludane natural products contain a characteristic annulated 5/6/4-ring system and have been reported to possess a diverse range of bioactivities. They have been predominantly isolated from higher fungi (96%) with very few examples from marine (1%) and plant (3%) sources (Figure 1). A total of 180 examples of this class have been isolated over a span of 70 years with almost half of these natural products being isolated in the last 20 years (Figure 2). 

The purpose of this review is to give an overview of sesquiterpenoid protoilludane natural products bearing an annulated 5/6/4-ring system and does not cover the structurally related illudins which are derived from protoilludane precursors. In this review, we will first discuss the isolation, structure elucidation, establishment of stereochemistry and summarise the bioactivities of sesquiterpenoid protoilludane natural products isolated to date from fungal origins beginning with those isolated from Basidiomycota followed by Ascomycota. We will then briefly cover protoilludanes isolated from other sources. We will acknowledge any known syntheses of these natural products but will not elaborate on these, as a comprehensive overview of synthetic attempts towards this class of natural products has been reported previously [14]. A short discussion on the biosynthetic studies conducted on these natural products has also been included. 

## 2. Basidiomycota

The Basidiomycota account for approximately 30% of the described species of higher fungi and include the smuts, rusts, jelly fungi, bracket fungi, stinkhorns, fairy clubs, bird’s nest fungi, puffballs, earthstars, toadstools and mushrooms [12,13,14]. This group of fungi are well-known producers of a wide range of terpenoid natural products, including protoilludane sesquiterpenoids [12,13,14]. Indeed, Basidiomycota account for over 82% of the protoilludanes produced by higher fungi (Figure 1).

### 2.1. Protoilludane Sesquiterpenes

**Figure 3:**
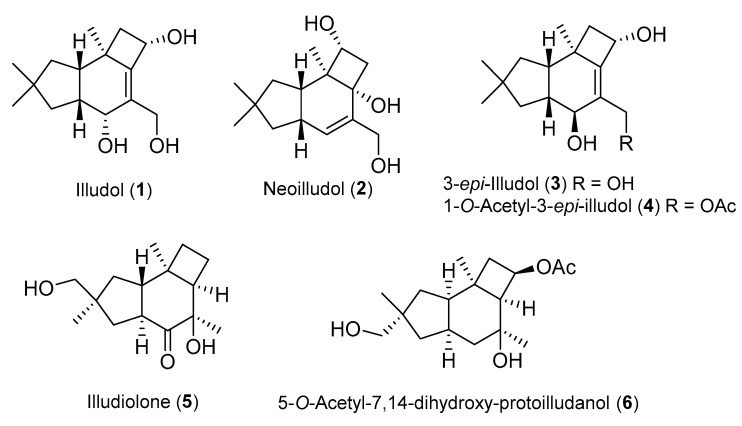
Structures of protoilludanes **1**–**6** isolated from the *Clitocybe* and *Omphalotus* genera of fungi.

The earliest reported protoilludane sesquiterpenoid, with the fused 5/6/4-ring system, was illudol (**1**) (Figure 3) [16]. It was isolated from the mushroom *Omphalotus olearius,* formerly *Clitocybe illudens*, in 1950 and reported as an unknown natural product at the time. Illudol (**1**) was tested for activity against several bacterial species including *Bacillus mycoides*, *Bacillus subtilis*, *Escherichia coli*, *Klebsiella pneumoniae*, *Mycobacterium smegmatis*, *Mycobacterium tuberculosis*, *Pseudomonas aeruginosa* and *Staphylococcus aureus* but was found to be inactive [16]. The planar structure of illudol (**1**) was not reported until 1967, while the absolute stereochemistry was only established in 1971 based on X-ray crystallography and was then successfully synthesised as a racemic mixture in the same year [17,18,19]. 

Three other protoilludanes (Figure 3) have since been isolated from the *Clitocybe* genus including neoilludol (**2**) from *C. illudens* and 3-*epi*-illudol (**3**) and 1-*O*-acetyl-3-*epi*-illudol (**4**) from *C. candicans* [20,21]. Determination of the relative stereochemistry of neoilludol (**2**) was achieved through nuclear Overhauser effect spectroscopy (NOESY) analysis and like illudol (**1**), the natural product exhibited no antimicrobial activity against either *S. aureus* NBRC13276 or *P. aeruginosa* ATCC15442 at a loading of 100 µg per disk [20,22]. The absolute stereochemistry of 3-*epi*-illudol (**3**) was established using the partial resolution method of Horeau on an acetonide derivative and analysis of the electronic circular dichroism (ECD) spectrum of 1-*O*-pivaloyl-3,6-dinitrobenzoyl derivative [21]. The relative stereochemistry of 1-*O*-acetyl-3-*epi*-illudol (**4**) was then established by consideration of shared biogenetic origin with **3** [21]. The latter was reported to exhibit antimicrobial activity against *E. coli* ATCC25922 with a minimum inhibitory concentration (MIC) of 32 µg/mL and a total synthesis towards the racemic mixture of the natural product was reported in 1997 [23,24].

Another protoilludane, illudiolone (**5**) was isolated from a mushroom, *Omphalotus illudens* [25]. X-ray crystallographic analysis of **5** allowed assignment of the structure and absolute configuration [25]. A related protoilludane, 5-*O*-acetyl-7,14-dihydroxy-protoilludanol (**6**), was isolated from *Conocybe siliginea*, the structure and relative configuration of which were assigned using rotating-frame nuclear Overhauser effect spectroscopy (ROESY) spectroscopic data [26]. The cytotoxicity of **6** was investigated against several human cancer cell lines including SK-BR-3, SMMC-7721, HL-60, PANC-1 and A-549 and was found to be non-cytotoxic [26].

**Figure 4:**
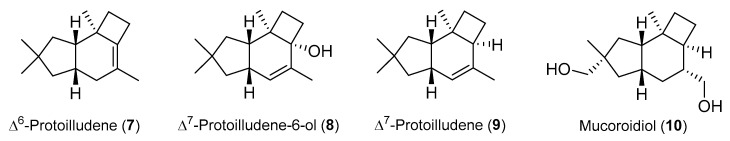
Structures of protoilludanes **7**–**10** isolated from the *Fomitopsis* and *Dictyostelium* genera.

Two examples of less oxygenated protoilludanes (Figure 4) were isolated from the mycelia of *Fomitopsis insularis,* Δ^6^-protoilludene (**7**) and Δ^7^-protoilludene-6-ol (**8**), of which the former has also been isolated from *O. olearius* [27,28]. In the isolation reports of Δ^6^-protoilludene, the sesquiterpenoid was shown to undergo isomerisation upon reaction with palladium on carbon to afford Δ^7^-protoilludene (**9**) [27]. Though Δ^7^-protoilludene (**9**) was only a semi-synthetic derivative at the time*,* it was the first of the three protoilludanes to be synthesised, albeit racemically [29]. Soon after, the synthesis of racemic Δ^6^-protoilludene (**7**) was achieved which allowed the relative stereochemistry of the natural product to be established [28]. Recent reports finally identified Δ^7^-protoilludene (**9**) as a natural product, isolated from slime mold *Dictyostelium discoideum* [30]. The relative stereochemistry of **9** was established by NOESY spectroscopic analysis with absolute stereochemistry being established by conversion of chirally pure deuterium labelled farnesyl diphosphate to Δ^7^-protoilludene (**9**) [30]. Another slime mold from the *Dictyostelium* genus, *D. mucoroides* afforded mucoroidiol (**10**), the relative configuration of which was assigned by analysis of NOESY data, while the absolute was suggested by consideration of shared biogenetic origins with **9** [31]. Mucoroidiol (**10**) exhibited no cytotoxicity (HeLa cells, IC_50_ > 40 µM) or antibacterial activity (*S. aureus* and *E. coli*) [31].

**Figure 5:**
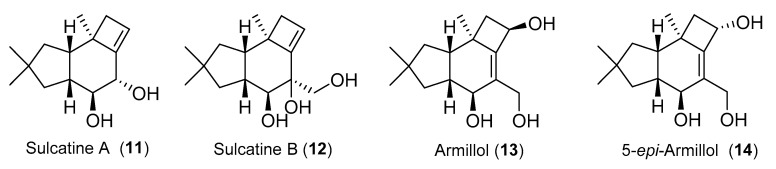
Structures of protoilludanes **11**–**14** isolated from *Laurilia sulcate.*

Four protoilludanes were isolated from the mushroom *Laurilia sulcate* (Figure 5), namely sulcatine A (**11**) and B (**12**), armillol (**13**) and 5-*epi*-armillol (**14**) [32,33]. The relative configurations of **11**–**14** were determined by analysis of NOESY NMR data. The absolute configuration of sulcatine A (**11**) was confirmed through analysis of an ECD spectrum of a dibenzoate derivative, while that of sulcatine B (**12**) and armillol (**13**) were established by biogenetic consideration [32,33]. Both sulcatine A (**11**) and B (**12**) have been shown to exhibit moderate antimicrobial activity against *E. coli* ATCC25922 (MIC 32 µg/mL) with **11** also exhibiting activity against *Mannheimia haemolytica* ATCC14003 (MIC 32 µg/mL) [34]. In addition, **12** and **14** have been reported to exhibit antifungal activity against *Cladiosporium cucumerinum* and *Candida albicans* at a loading of 50 µg per disk [33].

Another mushroom from the *Laurilia* genus, *L. tsugicola*, afforded tsugicolines A–E (**15**–**19**) [35,36]. The relative configurations of tsugicolines A–E (**15**–**19**) were established by NOESY spectroscopic analysis [35]. The absolute configuration of tsugicoline A (**15**) was determined by the partial resolution method of Horeau, while that of tsugicoline E (**19**) was determined by X-ray crystallographic analysis [35,36]. Tsugicolines A (**15**), C (**17**) and E (**19**) have been shown to exhibit moderate antimicrobial activity against *E. coli* ATCC25922 (MIC 32 µg/mL) with **15** and **17** also exhibiting activity against the Gram-negative bacterium, *M. haemolytica* ATCC14003 (MIC 32 µg/mL) [34]. Tsugicoline A (**15**) has also been shown to exhibit allelopathic activity against garden cress, *Lepidium sativum* [35]. 

**Figure 6:**
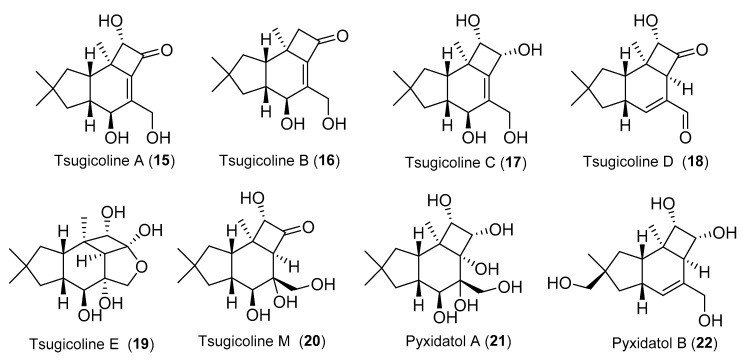
Structures of tsugicolines **15**–**20** and pyxidatols A (**21**) and B (**22**) isolated from the *Laurilia* and *Clavicorona* genera of fungi.

Another closely related compound, tsugicoline M (**20**), was isolated from the edible mushroom *Clavicorona pyxidate,* the relative configuration of which was assigned utilising NOESY NMR data [37]. Tsugicoline M (**20**) was investigated for antimicrobial activity against *E. coli*, *B. subtilis*, *S. aureus* and *Candida albicans,* at a concentration of 50 µg/mL, and cytotoxicity against HeLa cells, at 20 µg/mL and was found inactive in all assays [37]. The same mushroom also afforded two other novel protoilludanes (Figure 6), pyxidatol A (**21**) and B (**22**), along with the previously reported tsugicoline E (**19**) [38]. The relative configurations of pyxidatol A (**21**) and B (**22**) were resolved by ROESY analysis with the absolute configurations of **21** and **22** being assigned by consideration of shared biogenetic origins with **19** [37,38]. 

**Figure 7:**
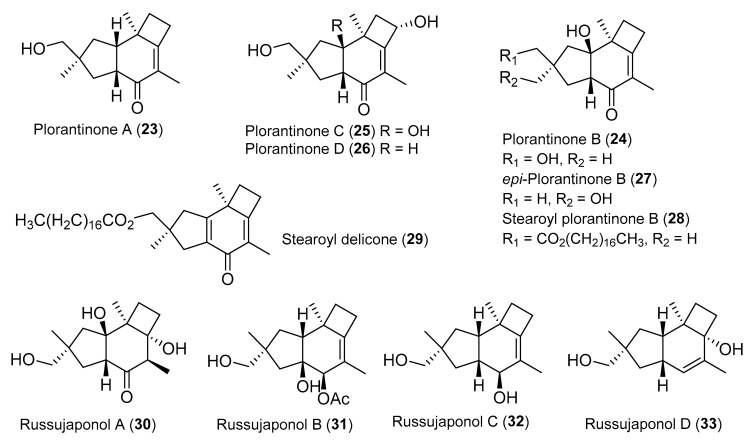
Structures of protoilludanes **23**–**33** isolated from the *Russula* genus of fungi.

The injured fruiting bodies of the mushroom *Russula delica* afforded five protoilludanes (Figure 7), plorantinone A–D (**23**–**26**) and *epi*-plorantinone B (**27**), while the intact fruiting bodies gave the first examples of fatty acid ester protoilludanes, stearoyl plorantinone B (**28**) and stearoyl delicone (**29**) [39,40,41]. The relative configurations of plorantinone A–C (**23**–**25**), stearoyl plorantinone B (**28**) and stearoyl delicone (**29**) were determined from NOESY data. The absolute configuration of plorantinone B (**24**) was established by analysis of ECD spectra, conformational analysis and molecular mechanics (MM3) calculations [39,40,41]. Subsequently, the absolute configurations of **23**, **25**, **28** and **29** were determined by comparison of optical rotation with plorantinone B (**24**) [39,40,41].

The fruiting bodies of another mushroom from the *Russula* genus, *R. japonica,* afforded russujaponols A–D (**30**–**33**) (Figure 7) [42]. Assignment of the relative configuration of **30**–**33** was achieved through analysis of ROESY and NOESY NMR data. The absolute configuration of russujaponol A (**30**) was assigned by X-ray crystallographic analysis of a benzoate derivative which in turn allowed the absolute configuration of russujaponols B–D (**31**–**33**) to be ascertained by biogenetic consideration. The cytotoxicity of **30**, **31** and **33** was investigated and all three natural products were found to be non-cytotoxic against 39 human tumour cell lines. Russujaponol A (**30**) was found to suppress the invasion of human fibrosarcoma cells (HT1080) into Matrigel with 63% inhibition at 3.73 µM [42].

**Figure 8:**
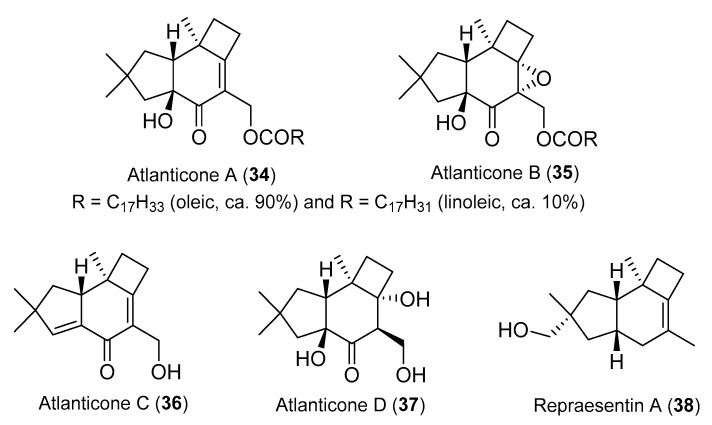
Structures of protoilludanes **34**–**38** isolated from the *Lactarius* genus of fungi.

Investigation of the fruiting bodies of a mushroom, *Lactarius atlanticus*, led to the discovery of atlanticones A–D (**34**–**37**) (Figure 8) [43]. As with the plorantinones, the intact fruiting bodies afforded fatty acid sesquiterpenoids, atlanticones A (**34**) and B (**35**) which were isolated as a mixture of fatty acid esters (ca. 90% oleic acid and ca. 10% linoleic acid), while atlanticones C (**36**) and D (**37**) were isolated from injured fruiting bodies. Both atlanticones A (**34**) and B (**35**) were investigated for antimicrobial activity against *S. aureus* and *E. coli* but were found to be inactive. The relative configurations of all four natural products were assigned by NOESY, while the absolute configuration of atlanticone A (**34**) was determined by a combination of PM3 calculations and ECD spectral comparison with plorantinone B (**24**) [43]. Both a racemic synthesis and enantioselective total synthesis of atlanticone C (**36**) have been reported [44,45]. 

Repraesentin A (**38**) (Figure 8), a closely related natural product to Δ^6^-protoilludene (**7**), was isolated from another mushroom from the *Lactarius* genus, *L. repraesentaneus* [46]. The relative configuration of the natural product was assigned utilising NOESY spectroscopic data, while the absolute configuration was assigned by consideration of common biosynthetic origins with **7**. Repraesentin A (**38**) was shown to promote radicle elongation of lettuce seedlings [46]. 

**Figure 9:**
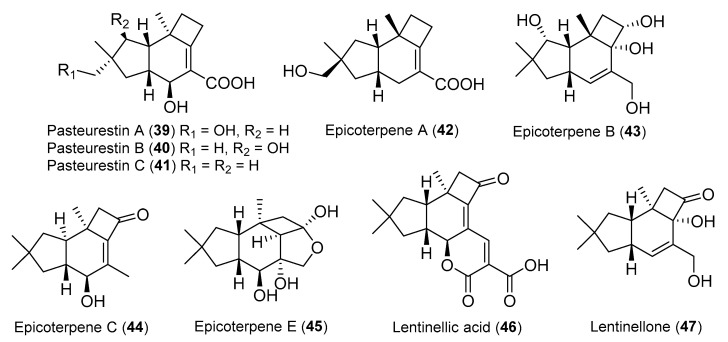
Structures of protoilludanes **39**–**47**.

The mushroom *Agrocybe aegerita* afforded three α,β-unsaturated carboxylic acid containing protoilludanes (Figure 9), pasteurestins A–C (**39**–**41**) [47,48]. Structure elucidation of all three natural products was achieved by spectroscopic analysis [47,48]. The stereoselective syntheses of both **39** and **40** have been reported [49,50]. This allowed assignment of the relative configurations of **39** and **40** by NOESY data and absolute configurations by comparison of optical rotations with the respective natural products [49,50]. The relative configuration of **41** was assigned by analysis of ROESY data, while the absolute configuration was suggested by biogenetic consideration [48]. The antimicrobial activities of pasteurestins A (**39**) and B (**40**) have been investigated against *Pasteurella multocida* N791 BBP 0101 (MIC 0.89 and 0.78 µg/mL, respectively) and *Mannheimia haemolytica* N811 BBP 0102 (MIC 1.56 and 0.78 µg/mL, respectively) where **40** was found to be a more potent inhibitor than **39** [34]. 

Another example of an α,β-unsaturated carboxylic acid containing protoilludane, epicoterpene A (**42**), was isolated from a co-culture of the honey fungus *Armillaria* sp. and the endophytic fungus *Epicoccum* sp. [51]. This co-culture also afforded three other protoilludanes, epicoterpene B (**43**), C (**44**) and E (**45**) (Figure 9). The relative configurations of all four epicoterpenes were assigned by analysis of ROESY data, while the absolute configurations were determined by comparison of experimental ECD spectra with time-dependent density functional theory (TDDFT) calculated ECD spectra. None of the four compounds exhibited cytotoxicity (IC_50_ > 40 µM) against HL-60, A-549, SMMC-7721, MCF7, SW480 cell lines or acetylcholinesterase inhibitory activity at a concentration of 50 µM [51].

The *Lentinellus* genus afforded two protoilludanes, lentinellic acid (**46**) and lentinellone (**47**) [52,53]. Lentinellic acid (**46**), isolated from *Lentinellus ursinus* and *L. omphalodes*, contains a lactone and an α,β-unsaturated carboxylic acid. Structure elucidation and assignment of the relative configuration of **46** was achieved by X-ray crystallography, while that of **47** was achieved by NOESY spectroscopic analysis. The absolute configuration of **47** was tentatively assigned by consideration of shared biogenetic origins. Both natural products were investigated for their antimicrobial activity against a variety of strains including; *Bacillus brevis, B. subtilis, Corynebacterium insidiosum, E. coli, Micrococcus luteus, Mycobacterium phlei* and *Streptomyces* sp., where lentinellone (**47**) was found to be inactive. Alternately, lentinellic acid (**46**) exhibited potent antimicrobial activity (MIC 1–5 µg/mL) against *Aerobacter aerogenes*, *B. brevis*, *C. insidiosum* and moderate activity against *Acinetobacter aerogenes* (MIC 20–50 µg/mL), *B. subtilis* (MIC 20–50 µg/mL), *M. luteus* (MIC 10–20 µg/mL), *Proteus vulgaris* (MIC 20–50 µg/mL), *S. aureus* (MIC 20–50 µg/mL) and *Streptomyces* sp. (MIC 10–20 µg/mL). In addition, **46** also exhibited antifungal activity against *Absidia glauca* and *Nematospora coryli* at a loading of 100 µg per disk. The cytotoxicity of **47** was investigated against various cancer cell lines where it exhibited moderate cytotoxicity against HL-60 (IC_50_ 20 µg/mL) [52,53].

**Figure 10:**
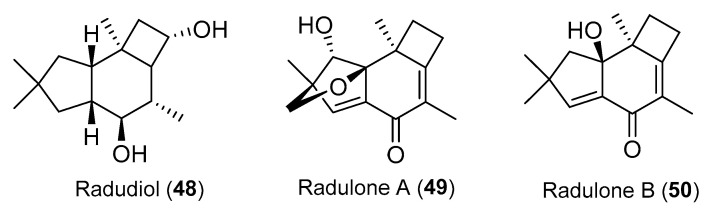
Structures of protoilludanes **48**–**50** isolated from *Radulomyces confluens.*

Three protoilludanes, radudiol (**48**), and radulone A (**49**) and B (**50**), were isolated from the crust fungus, *Radulomyces confluens* (Figure 10) [54]. Radudiol (**48**) is another example of a saturated protoilludane, while radulone A (**49**) has four rings, one of which contains an oxygen bridge. The relative configurations of all three natural products were assigned by analysis of NOESY NMR data. The cytotoxicity of **48**–**50** was investigated against HeLa S3, HL-60 and L-1210 cell lines where **49** (IC_50_ 16, 2, and 1 µM, respectively) exhibited potent cytotoxicity against all three cell lines, while radudiol (**48**) and radulone B (**50**) were non-cytotoxic. Radulone A (**49**) also exhibited weak antimicrobial activity against *Corynebacterium insiduodum*, *Micrococcus luteus*, *Nematospora coryli* and *Ustilago nuda* with an MIC of 20 µM as well as *Streptomyces bikiniensis*, *Saccharomyces cerevisiae* and *Mucor miehei* with an MIC of 41 µM. In addition, **49** was found to be a potent inhibitor of human (IC_50_ 2 µM) and bovine (IC_50_ 12 µM) platelet aggregation induced by adenosine diphosphate (conc 25 µM) [54]. The stereoselective synthesis of the non-natural enantiomer of radudiol (**48**) has been reported allowing assignment of the absolute configuration of the natural product by comparison of optical rotations [55].

**Figure 11:**
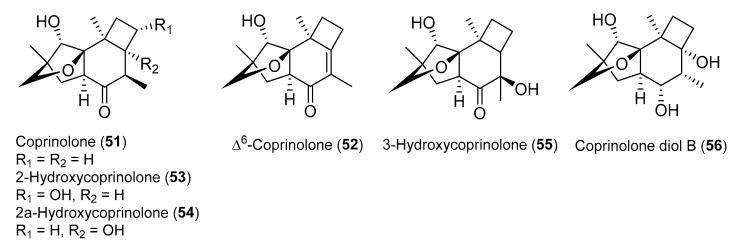
Structures of coprinolones **51**–**56** isolated from the *Coprinus* and *Granulobasidium* genera of fungi.

Two examples of oxygen-bridged protoilludanes (Figure 11), coprinolone (**51**) and Δ^6^-coprinolone (**52**), were isolated from *Coprinus psychromorbidus* [56,57]. The structures of both natural products were assigned by spectroscopic analysis and the relative stereochemistry of coprinolone (**51**) was determined by analysis of NOESY data of the natural product and its derived isomers [56,57]. Further examples of oxygen-bridged protoilludanes, including 2-hydroxycoprinolone (**53**), 2a-hydroxycoprinolone (**54**), 3-hydroxycoprinolone (**55**) and coprinolone diol B (**56**) were isolated from the plant pathogen *Granulobasidium vellereum* [58,59]. Structure elucidation and establishment of the absolute configurations of 2-hydroxycoprinolone (**53**), 2a-hydroxycoprinolone (**54**) and 3-hydroxycoprinolone (**55**) were achieved using a ROESY spectroscopic data and analysis of ECD spectra. The absolute configuration of coprinolone diol B (**56**) was established by consideration of shared biogenetic origin with that of coprinolone diol, a semi-synthetic derivative from coprinolone (**51**). The antimicrobial activities of **53**–**56** were investigated against several species including *Penicillium canescens*, *Fusarium oxysporum* and *Heterobasidion occidentale* at concentrations up to 100 µg/mL and all four natural products were found to be inactive [58,59].

**Figure 12:**
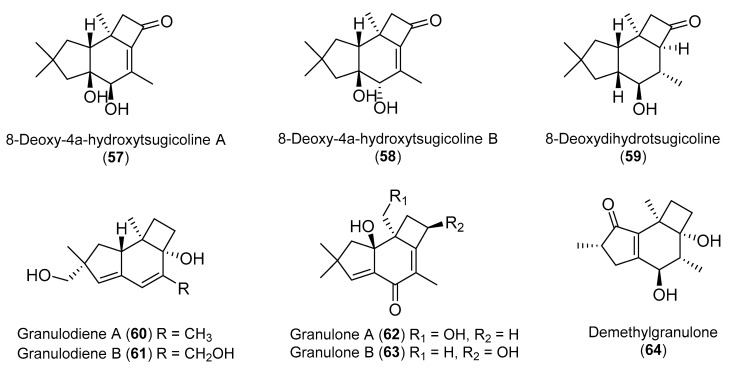
Structures of protoilludanes **57**–**64** isolated from *Granulobasidium vellereum.*

Other protoilludanes isolated from *Granulobasidium vellereum* include 8-deoxy-4a-hydroxytsugicoline A (**57**) and B (**58**), 8-deoxydihydrotsugicoline (**59**), granulodienes A (**60**) and B (**61**), granulones A (**62**) and B (**63**) and demethylgranulone (**64**) (Figure 12) [58,59]. Determination of the relative configurations of **57**–**64** was achieved by ROESY spectroscopic analysis. The absolute configurations **59**–**61** was established by ECD spectral analysis, while those of **57**, **58**, and **62**–**64** were assigned upon biosynthetic consideration with other protoilludanes. The antifungal activities of **57**–**64** were investigated against *Penicillium canescens*, *Fusarium oxysporum* and *Heterobasidion occidentale* at concentration up to 100 µg/mL and none of the natural products exhibited activity [58,59]. The stereoselective synthesis of the non-natural enantiomer of 8-deoxydihydrotsugicoline (**59**) has been reported [55].

**Figure 13:**
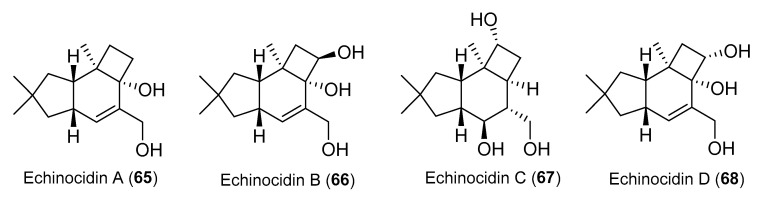
Structures of echinocidins A–D (**65**–**68**) from *Echinodontium tsugicola.*

The crust fungus, *Echinodontium tsugicola*, afforded four protoilludanes named echinocidins A–D (**65**–**68**) (Figure 13) [60,61]. The relative configurations of **65**–**68** was assigned by NOESY spectroscopic analysis where **65** and **67** were based on the natural products, while **66** and **68** were on a diacetyl and acetonide derivative, respectively. The absolute configurations of these natural products were ascertained by consideration of shared biogenetic origins with tsugicoline A (**15**) and B (**16**). The antimicrobial activities of echinocidin A (**65**) and B (**66**) were investigated against *C. albicans* ATCC2019, *S. aureus* NBRC13276 and *P. aeruginosa* ATCC15442 at a loading concentration of 100 µg per disk and both natural products were found to be inactive [60,61]. All four natural products **65**–**68** were investigated in a lettuce seedling bioassay at a concentration of 100 ppm where they were observed to accelerate primary root growth to 117%, 180%, 190% and 179% of controls, respectively [60,61]. The total syntheses of racemic echinocidin B (**66**) and D (**68**) have been reported [62].

### 2.2. Protoilludane Sesquiterpene Aryl Esters

The genus *Armillaria* has been one of the most extensive producers of aryl ester containing protoilludanes. These protoilludanes can be divided into three types of which the first two are classified based on the position of the double bond in the protoilludane skeleton, i.e., Δ^2,4^-protoilludene (e.g., Δ^6^-protoilludene) and Δ^2,3^-protoilludene (e.g., Δ^7^-protoilludene) skeletons, while the third is classified by the absence of double bonds in the 5/6/4-ring system. 

**Figure 14:**
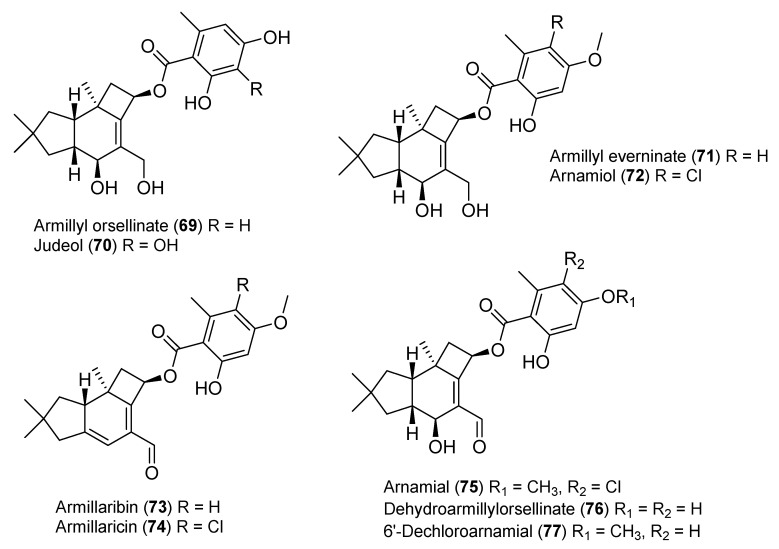
Structures of Δ^2,4^-protoilludene-containing aryl sesquiterpenoids **69**–**77** isolated from *Armillaria mellea.*

Aryl sesquiterpenoids of the first type, of which only a few examples exist, include armillyl orsellinate (**69**) [63], judeol (**70**) [64], armillyl everninate (**71**) [65], arnamiol (**72**) [65], armillaribin (**73**) [66], armillaricin (**74**) [67], arnamial (**75**) [68], dehydroarmillyl orsellinate (**76**) [69] and 6’-dechloroarnamial (**77**) [70], all isolated from the honey fungus *Armillaria mellea* (Figure 14). Structure elucidation and determination of the absolute configurations of armillyl orsellinate (**69**) and armillaricin (**74**) were achieved utilising a combination of spectroscopic and X-ray crystallographic analysis [63,67,71]. Subsequently, the relative configurations of arnamial (**75**) and 6’-dechloroarnamial (**77**) were ascertained by biosynthetic consideration [68,70]. Both armillyl orsellinate (**69**) and judeol (**70**) exhibited antimicrobial activity against *B. subtilis* ATCC6633 (minimum loading of 5.6 and 8.7 µg per disk, respectively) and *S. aureus* ATCC53156 (minimum loading of 5.6 and 8.7 µg per disk, respectively) with **69** also exhibiting activity against *E. coli* (MIC 1.0 µg) and *C. albicans* (MIC 1.0 µg) on bioautographic assays [63,64,72]. Arnamial (**75**), dehydroarmillyl orsellinate (**76**) and 6’-dechloroarnamial (**77**) were also investigated for their antimicrobial properties and were reported to moderately inhibit the growth of *Penicillium notatum*, *Aspergillus flavus* and *Aspergillus nidulans* [70]. In addition, **75** also exhibited potent activity against *Tapinella panuoides*, *Pleurotus ostreatus*, *Omphalotus illudens*, *Fomitopsis pinicola*, *P. oxalicum*, *A. flavus* and *Streptomyces scabies* [73]. Four of these natural products (**74**–**77**) have been shown to exhibit cytotoxicity against several human cell lines [68,69,70,74]. Armillaricin (**74)** was shown to be potently cytotoxic towards H460, MCF7, HT-29 and CEM cell lines (IC_50_ 5.5, 4.8, 4.6, and 5.8 µM, respectively). Meanwhile, **75**–**77** exhibited moderate to potent cytotoxicity towards HUVEC (GI_50_ 2.0, 5.3, 5.1 µM) MCF7 (GI_50_ 15.4, 8.0, 4.1 µM), K-562 (GI_50_ 2.3, 5.0, 4.1 µM) and HeLa (CC_50_ HeLa 4.9, 15.2, 12.3 µM) cell lines. Additionally, **75** was also found to be cytotoxic towards HCT-116, Jurkat and CCRF-CEM (IC_50_ 10.69, 3.9, and 8.91 µM, respectively) cell lines, while **76** exhibited cytotoxicity towards Jurkat (IC_50_ 16.9 µM) cells [68,69,70,74]. 

**Figure 15:**
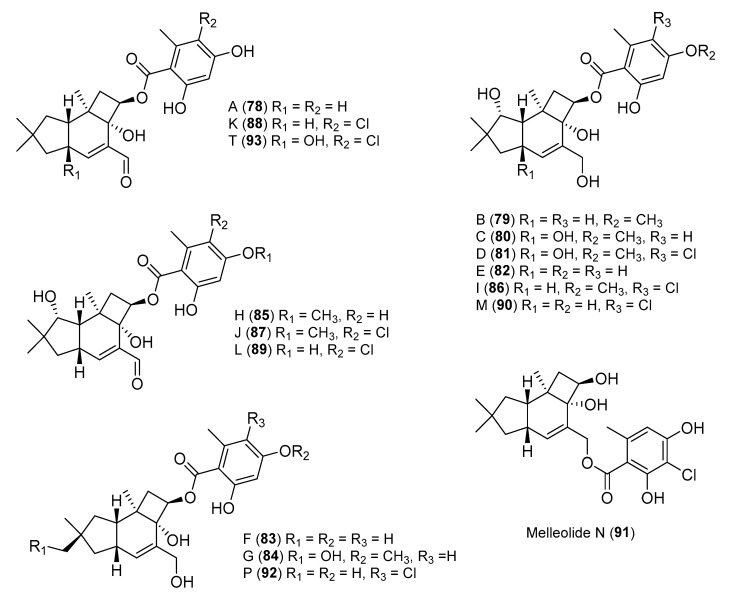
Structures of melleolides **78**–**93** from the *Armillaria* genus of fungi.

The second type of aryl ester sesquiterpenoids, containing the Δ^2,3^-protoilludene skeleton, constitute the largest portion of this protoilludane class. Melleolides A–N (**78**–**91**) [74,75,76,77,78], P (**92**) [79] and T (**93**) (Figure 15) [79] were isolated from *Armillaria mellea* with the exception of melleolide I (**86**) [80] which was first isolated from another mushroom *A*. *novae-zelandiae* along with melleolide J (**87**) (also named armillarikin) [81]. Of these natural products, melleolide N (**91**) is the only example with esterification at the C-1 hydroxyl group [74]. The structure and the absolute configuration of the first melleolide (**78**) was solved by X-ray crystallographic analysis [75]. Assignment of the relative configurations of melleolides B (**79**), C (**80**), E–K (**82**–**88**) and N (**91**) were achieved by extensive NOESY spectroscopic analysis [74,76,77,78,80], while that of melleolide P (**92**), also known as 6’-chloromelleolide F, was ascertained by consideration of common biogenetic origins with melleolide F (**83**) [70,79]. The absolute stereochemistry of melleolide L (**89**) was established by X-ray crystallographic analysis conducted on a 2,4-dinitrophenylhydrazone derivative [78]. On the other hand, the absolute configurations of melleolide D (**81**) and M (**90**) were determined by semi-synthesis of the natural products and subsequent comparison of optical rotations [78,82]. Finally, the absolute configuration of melleolide T (**93**), also named 13-hydroxymelleolide K, was assigned by comparison of ECD spectra with melleolide D (**81**) [79,83]. Melleolides A (**78**) (IC_50_ HepG2 4.95 µg/mL, L02 16.05 µg/mL), F (**83**) (IC_50_ MCF7 8.3 µM, H460 5.1 µM), I also known as 5’-chloromelleolide (**86**) (IC_50_ A-549 20.11 µM, MCF7 30.06 µM), J (**87**) (IC_50_ MCF7 4.4 µM, H460 5.7 µM, HL-60 14.11 µM, A-549 14.73 µM, SMMC-7721 17.41 µM), N (**91**) (IC_50_ HT-29 7.1 µM, H460 5.5 µM, CEM 5.4 µM) and P (**92**) (IC_50_ MCF7 4.8 µM, H460 4.5 µM, CEM 28.8 µM) have been reported to be cytotoxic against human cancer cell lines [70,72,74,84,85]. The antimicrobial activity of some of the melleolides have also been investigated. Melleolide A (**78**) was reported to exhibit antimicrobial activity against *B. subtilis* ATCC6633 (MIC 0.5 µg), *E. coli* (MIC 1.0 µg), *C. cucumerinum* (MIC 1.0 µg) and *C. albicans* (MIC 1.0 µg) in bioautographic assays, while melleolides B–D (**79**–**81**) were reported to exhibit antibacterial activity against *B. subtilis* ATCC6633, *B. cereus* ATCC10702 and *E. coli* ATCC10536 at a minimum loading of 10 µg per disk [72,76]. In addition, melleolides K–M (**88**–**90**) were investigated for antimicrobial activity against several species, where **89** and **90** were found to be inactive [78]. Melleolide K (**88**) exhibited moderate activity against *Micrococcus luteus* IF03333 (MIC 12.5 µg/mL), *B. subtilis* PCI219 (MIC 6.25 µg/mL), *Corynebacterium bovis* 1810 (MIC 25 µg/mL), *Saccharomyces cerevisiae* (MIC 25 µg/mL), *Trichophyton rubrum* IFO9185 (MIC 25 µg/mL), *S. aureus* (FDA209P (MIC 6.25 µg/mL), Smith (MIC 12.5 µg/mL), MS9610 (MIC 12.5 µg/mL)) and MRSA (MS16526 (MIC 12.5 µg/mL), TY-04282 (MIC 12.5 µg/mL)) [78]. The total syntheses of racemic melleolide A (**78**) and F (**83**) were reported recently [62].

**Figure 16:**
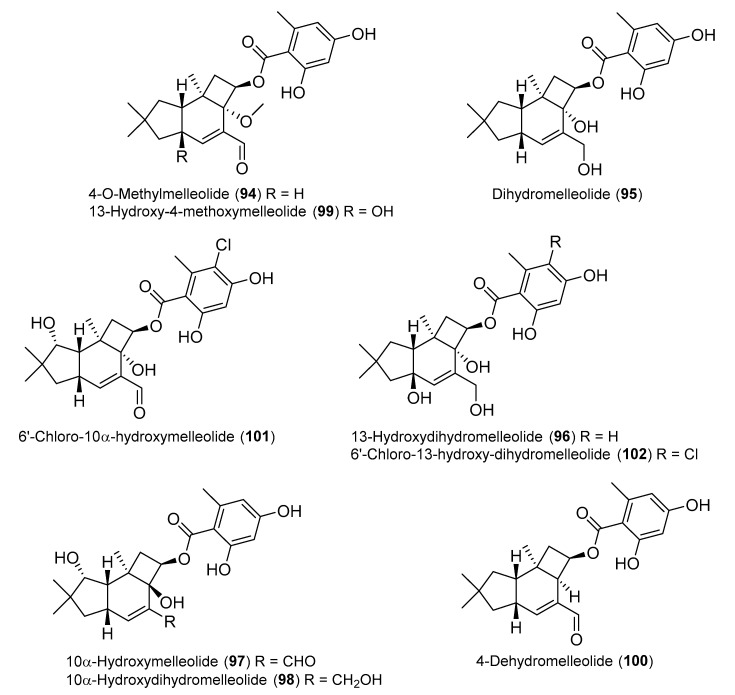
Structures of melleolides **94**–**102** isolated from *Armillaria mellea.*

Other melleolides isolated from *A. mellea* include 4-*O*-methylmelleolide (**94**) [64], dihydromelleolide (**95**) [86], 13-hydroxydihydromelleolide (**96**) [87], 10α-hydroxymelleolide (**97**) [87], 10α-hydroxydihydromelleolide (**98**) [87], 13-hydroxy-4-methoxymelleolide (**99**) [88], 4-dehydromelleolide (**100**) [86], 6’-chloro-10α-hydroxymelleolide (**101**) [72] and 6’-chloro-13-hydroxy-dihydromelleolide (**102**) [69]. Structure elucidation and confirmation of the absolute stereochemistry of 4-*O*-methylmelleolide (**94**) was achieved utilising X-ray crystallography [64]. The relative configurations of 13-hydroxydihydromelleolide (**96**), 13-hydroxy-4-methoxymelleolide (**99**) and 6’-chloro-10α-hydroxymelleolide (**101**) (Figure 16) were determined using NOESY spectroscopic data [72,87,88]. Unlike the other melleolides, 10α-hydroxymelleolide (**97**) and 6’-chloro-13-hydroxy-dihydromelleolide (**102**) were found to be weakly cytotoxic and against CCRF-CEM (IC_50_ 63.23 µM) and Jurkat (IC_50_ 46.6 µM) cells, respectively [68,69]. Upon investigation of the antibacterial properties of some of these compounds, **94** exhibited activity against *B. subtilis* ATCC6633 and *S. aureus* ATCC53156 at a minimum loading of 5.6 µg per disk, while **97** and **101** exhibited activity against *B. subtilis* ATCC6633 (MIC 0.5 µg) and *E. coli* (MIC 1.0 µg) on bioautographic assays [64,72]. In addition, the latter two also exhibited antifungal activity against *C. cucumerinum* and *C. albicans* with MICs of 1.0 µg on bioautographic assays [72].

**Figure 17:**
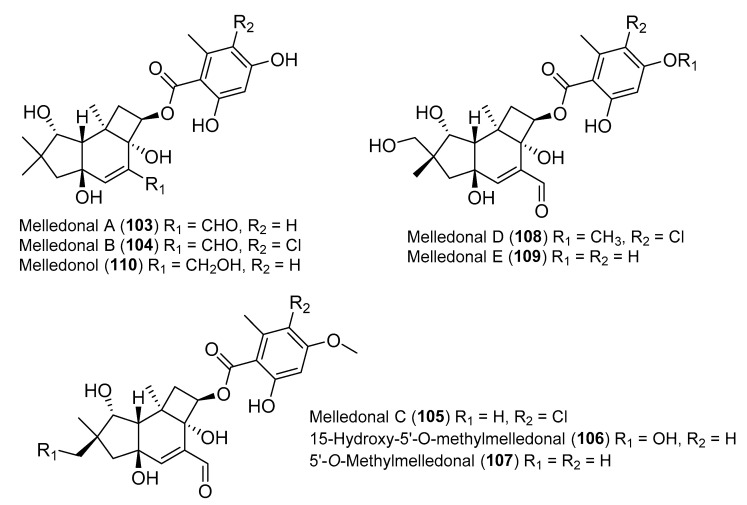
Structures of melledonals **103**–**109** and melledonol (**110**) isolated from the *Armillaria* and *Clitocybe* genera of fungi.

Of the melledonals (Figure 17), a group of α,β-unsaturated aldehydes, melledonals A–C (**103**–**105**) [82,89], 15-hydroxy-5’-*O*-methylmelledonal (**106**) [90] and 5’-*O*-methylmelledonal (**107**) [90] were isolated from *A. mellea*, while melledonals D (**108**) and E (**109**) were isolated from *Clitocybe elegans* [77]. The relative configurations of **105** and **108** were assigned by NOESY spectroscopic analysis of the natural products, while for **103** and **106**, analysis was conducted on a diacetate derivative and a dibenzoate derivative, respectively [77,82,89,90]. On the other hand, the relative configurations of **104**, **107** and **109** were assigned by biogenetic consideration [77,82,89,90]. The absolute configuration of melledonal C (**105**) was established by X-ray crystallographic analysis [82]. Of the melledonals, only **105** was found to exhibit cytotoxicity against human cancer cell lines, albeit weakly (CEM IC_50_ 49.6 µM, HT-29 IC_50_ 85.6 µM) [74]. Melledonal (**103**) and melledonal C (**105**) were investigated for antifungal activity; only **103** exhibited weak activity (MIC 50 µg/mL) against *T. panuoides*, *O. illudens* and *F. pinicola* [73]. In addition, melledonals A–C (**103**–**105**) were found to exhibit antimicrobial activity against *B. cereus* ATCC10702 and *B. subtilis* ATCC6633 at a loading of 100 µg per disk with **105** also exhibiting activity against *E. coli* (MIC 1.0 µg) and *C. albicans* (MIC 3.0 µg) in bioautographic assays [72,82]. A dihydro derivative of melledonal, melledonol (**110**) was also isolated from *A. mellea* [89]. Structure elucidation was conducted by NMR spectroscopic analysis and the relative configuration assigned by biosynthetic consideration [89]. 

**Figure 18:**
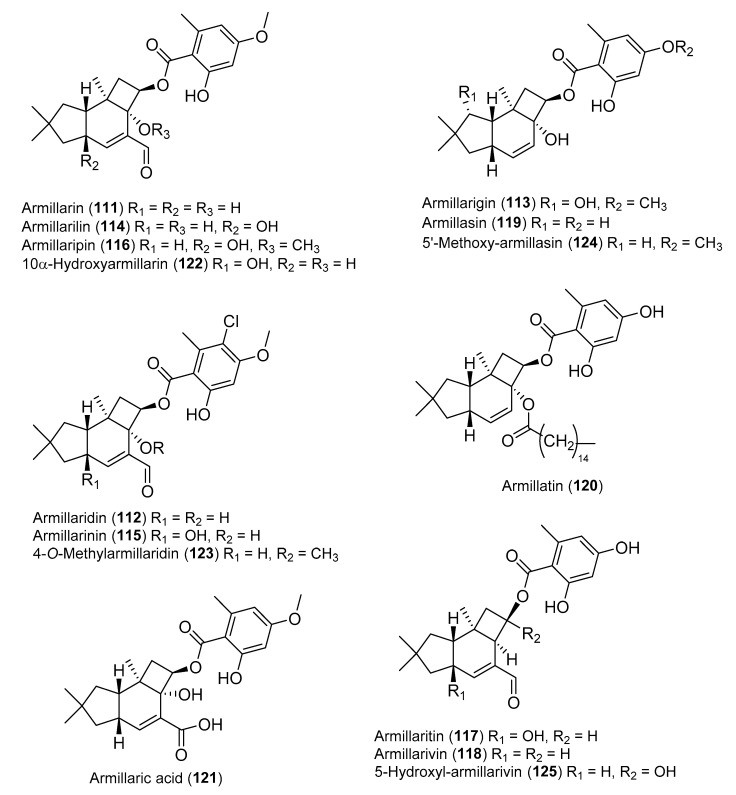
Structures of Δ^2,3^-protoilludene-containing aryl sesquiterpenoids **111**–**125** isolated from *Armillaria mellea.*

Other aryl ester sesquiterpenoids containing Δ^2,3^-protoilludene skeletons isolated from *A. mellea* include armillarin (**111**) [91], armillaridin (**112**) [91], armillarigin (**113**) [81], armillarilin (**114**) [92], armillarinin (**115**) [92], armillaripin (**116**) [93], armillaritin (**117**) [94], armillarivin (**118**) [94], armillasin (**119**) [95], armillatin (**120**) [95], armillaric acid (**121**) [96], 10α-hydroxyarmillarin (**122**) [97], 4-*O*-methylarmillaridin (**123**) [97], 5’-methoxy-armillasin (**124**) [84] and 5-hydroxyl-armillarivin (**125**) [84]. Armillasin (**119**), armillatin (**120**) and 5’-methoxy-armillasin (**124**) are examples of norsesquiterpenoids, while armillaric acid (**121**) is the first example of an α,β-unsaturated carboxylic acid-containing aryl sesquiterpenoid (Figure 18) [84,95,98]. Assignment of the absolute stereochemistry of **111** was achieved by X-ray crystallographic analysis, while that of **112** was ascertained by consideration of shared biogenetic origins [91]. The relative configurations of **119**, **121**, **124** and **125** were established by NOESY spectroscopic analysis, while that of **120**, **122** and **123** were assigned by biogenetic consideration [84,95,97,98]. Several of these natural products have been reported to exhibit moderate cytotoxicity towards human cancer cell lines [69,70,74,84,85]. Armillarin (**111**) and armillaridin (**112**) have been shown to be moderately cytotoxic towards HUVEC (GI_50_ 9.9, 7.8 µM), K-562 (GI_50_ 9.9, 8.9 µM), MCF7 (GI_50_ 11.6, 7.8 µM) and HeLa (CC_50_ 16.7, 9.2 µM) cell lines with the latter also exhibiting cytotoxicity towards H460, CEM, HepG2 and L02 (IC_50_ 4.5 µM, 5.1 µM, 13.37 µg/mL and 12.15 µg/mL) cell lines. Meanwhile, armillarilin (**112**), armillaritin (**117**), armillasin (**119**) and 5-hydroxyl-armillarivin (**125**) exhibited cytotoxicity towards HepG2 (IC_50_ 13.25, 12.26, 15.63, 18.03 µg/mL) and L02 (IC_50_ 18.00, 31.95, 14.38, 22.70 µg/mL) cells. In addition, the cytotoxicity of armillarigin (**113**) (IC_50_ HL-60 14.27 µM, SMMC-7721 19.51 µM, A-546 10.01 µM, MCF7 10.67 µM, SW480 19.19 µM) and 4-*O*-methylarmillaridin (**123**) (IC_50_ MCF7 6.7 µM, Jurkat 4.1 µM, HeLa 18.8 µM) have also been investigated [69,70,74,84,85]. On the other hand, armillaric acid (**121**) has been shown to exhibit antimicrobial activity against *S. aureus* 209PJC (19.8 mm), *M. lutaus* ATCC9341 (8.8 mm) and *C. albicans* NHL4019 (10.1 mm) in zone of inhibition assays at a loading of 250 µg/mL per disk [96,98]. 

**Figure 19:**
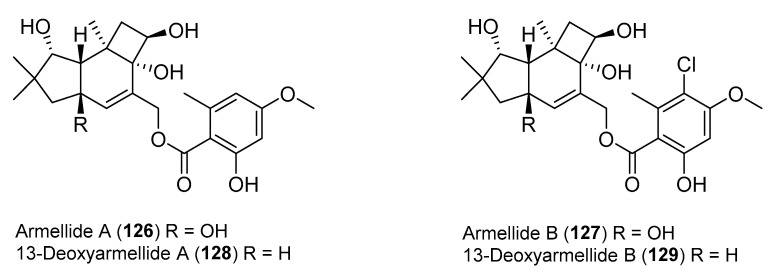
Structures of armellides **126**–**129** isolated from the *Armillaria* genus of fungi.

Armellides A (**126**) and B (**127**), isolated from *Armillaria novae-zelandiae*, were the first examples of aryl ester protoilludanes esterified at the C-1 hydroxyl group (Figure 19) [80]. Two related armellides, 13-deoxyarmellides A (**128**) and B (**129**) were later reported from *A. mellea* [70]. The relative configurations of armellides A (**126**) and B (**127**) were determined by NOESY NMR data, while those of 13-deoxyarmellides A (**128**) and B (**129**) were assigned by consideration of shared biogenetic origins [70,80]. The cytotoxicity of **127**–**129** have been investigated and only **129** was found to be cytotoxic, albeit weakly against HUVEC (GI_50_ 91.0 µM), K-562 (GI_50_ 91.8 µM), MCF7 (GI_50_ 90.8 µM) and HeLa (CC_50_ 89.5 µM) cell lines [69,70].

**Figure 20:**
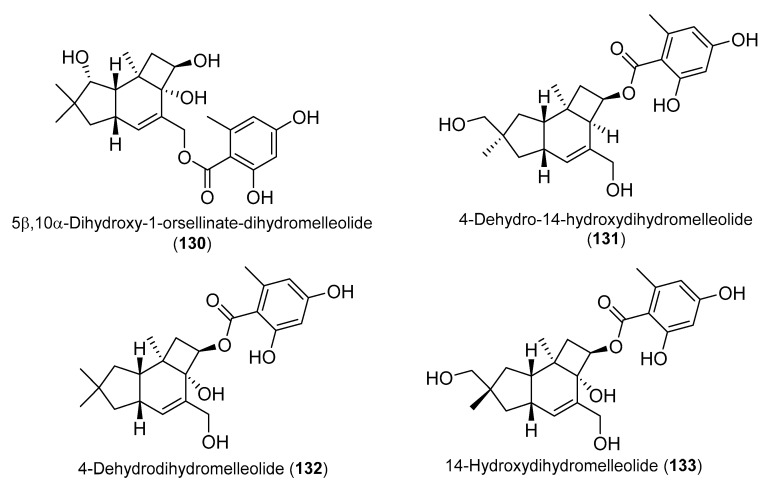
Structures of melleolides **130**–**133** isolated from *Armillaria tabescens.*

Another mushroom from the *Armillaria* genus, *A. tabescens,* afforded four melleolides including 5β,10α-dihydroxy-1-orsellinate-dihydromelleolide (**130**), 4-dehydro-14-hydroxydihydromelleolide (**131**), 4-dehydrodihydromelleolide (**132**) and 14-hydroxydihydromelleolide (**133**) (Figure 20) [88]. Assignment of the relative configuration of all four natural products was achieved utilising NOESY spectroscopic data [88].

**Figure 21:**
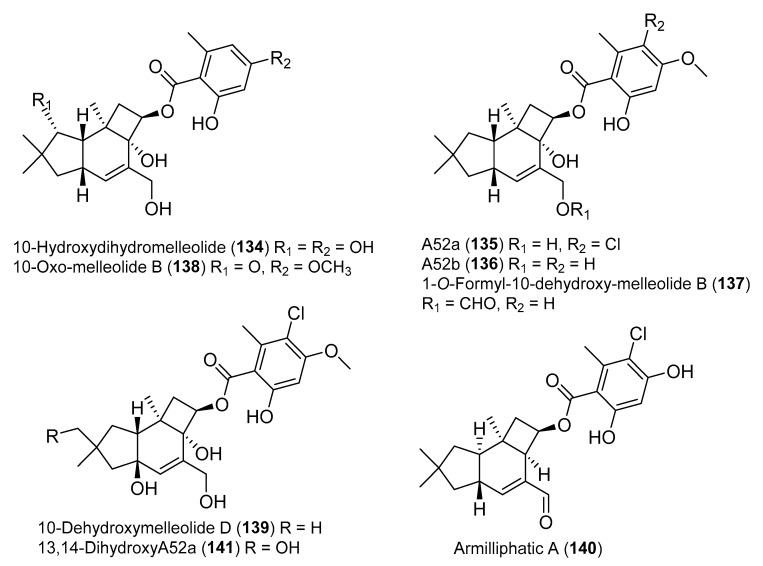
Structures of Δ^2,3^-protoilludene-containing aryl sesquiterpenoids **134**–**141** isolated from the *Armillaria* genus of fungi.

More examples of Δ^2,3^-protoilludene-type sesquiterpenoid aryl esters were isolated from several undetermined *Armillaria* spp. (Figure 21). These included 10-hydroxydihydromelleolide (**134**) [86], A52a (**135**) [99], A52b (**136**) [99], 1-*O*-formyl-10-dehydroxy-melleolide B (**137**) [85], 10-oxo-melleolide B (**138**) [85], 10-dehydroxymelleolide D (**139**) [83] and armilliphatic A (**140**). Structure elucidation of all seven natural products was achieved by NMR spectroscopic analysis [51,83,85,86,99]. The relative configurations of A52b (**136**) (also named 10-dehydroxy-melleolide B) and armilliphatic A (**140**) were determined by analysis of ROESY data, while those of 1-*O*-formyl-10-dehydroxy-melleolide B (**137**) and 10-oxo-melleolide B (**138**) were assigned by biogenetic consideration [51,85,99]. The absolute configurations of **139** and **140** were established by comparison of experimental ECD spectra with those of melleolide D (**81**) and TDDFT calculated data, respectively [51,83]. A52a (**135**), A52b (**136**), 1-*O*-formyl-10-dehydroxy-melleolide B (**137**), 10-oxo-melleolide B (**138**) and armilliphatic A (**140**) were investigated for cytotoxicity against several cell lines where **138** was found to be non-cytotoxic (IC_50_ > 40 µM) [51,69,85]. Aryl esters **135**–**137** and **140** exhibited moderate cytotoxicity against HL-60 (IC_50_ 17.06, 17.79, 14.50, 15.80 µM), SMMC-7721 (IC_50_ 17.77, 20.90, 23.16, 19.42 µM), A-549 (IC_50_ 15.89, 16.79, 18.41, 15.93 µM), MCF7 (IC_50_ 14.10, 16.49, 5.34, 19.22 µM) and SW480 (IC_50_ 15.70, 17.44, 10.77, 23.03 µM) cell lines [51,69,85]. In addition, **135** was also investigated for cytotoxicity against Jurkat (IC_50_ 10.4 µM), HeLa (IC_50_ 40.0 µM) and K-562 (IC_50_ 38.9 µM) cell lines [69]. The antimicrobial activities of **135** and **136** were investigated against *Heterobasidion annosum*, *Gloeophyllum abietinum*, *E. coli* and *S. aureus* on disks. However, neither were found to be active [99]. Armilliphatic A (**140**) [51], which was isolated from a co-culture with *Epicoccum* sp., was found to exhibit weak acetylcholinesterase inhibitory activity (IC_50_ 23.85 ± 0.20 µM) [51]. Another aryl ester, the 13,14-dihydroxylated derivative of A52a (**141**), was isolated from *A. mellea* and was found to be non-cytotoxic against MCF7, Jurkat, HeLa and K-562 cell lines (IC_50_ > 100 µM) [69]. 

**Figure 22:**
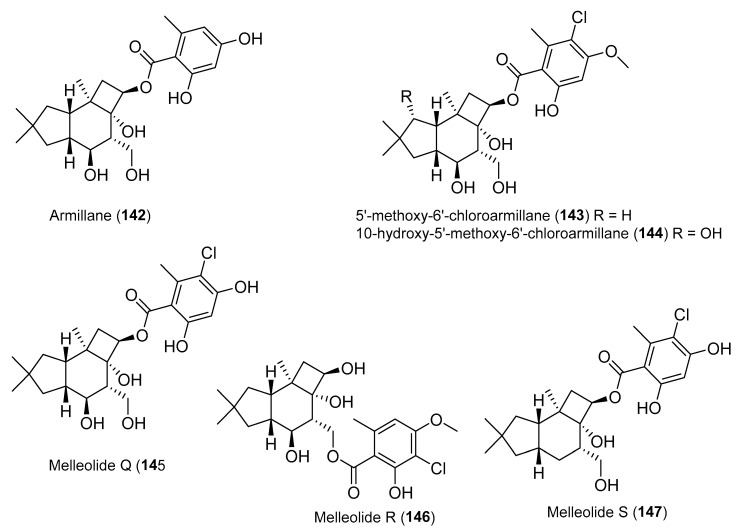
Structures of saturated protoilludane-containing aryl sesquiterpenoids **142**–**147** isolated from *Armillaria mellea.*

The last of the three types of aryl ester protoilludane structures can be identified by absence of double bonds in the 5/6/4-ring system. The first example of this type of aryl ester sesquiterpenoid, armillane (**142**) also known as armillarizin, was isolated from *A. mellea* (Figure 22) [97]. Other examples of aryl esters with saturated protoilludane skeletons from *A. mellea* include 5’-methoxy-6’-chloroarmillane (**143**) [62], 10-hydroxy-5’-methoxy-6’-chloroarmillane (**144**) [70] and melleolides Q–S (**145**–**147**) [74,79]. The relative configurations of **142**, **145** and **146** were determined by NOESY spectroscopic analysis, while those of **143** and **144** were assigned by consideration of common biogenetic origins [69,70,74]. Melleolide R (**146**) is another example of an aryl ester sesquiterpenoid with esterification at the hydroxyl of C-1 [74]. Both **145** and **146** were shown to exhibit moderate cytotoxicity against several human cancer cell lines including MCF7 (IC_50_ 1.5 and 3.7 µM, respectively) and CEM (IC_50_ 10.3 and 3.4 µM, respectively) [74]. In addition, **143** and **144** were also found to be cytotoxic albeit weakly (IC_50_ > 20 µM and > 40 µM, respectively) against HUVEC, K-562 and MCF7 cell lines [69,70].

## 3. Ascomycota

Ascomycota, which include cup and flask fungi, moulds and most yeasts, account for approximately 60% of the described species of higher fungi and are well known for producing polyketides, non-ribosomal peptides and indole-derived natural products [12]. Fewer sesquiterpenoids have been reported from Ascomycota in comparison to Basidiomycota with only a few species of fungi to date found to have produced protoilludanes bearing the annulated 5/6/4-ring system. The first protoilludane to be isolated from an Ascomycete, *Ceratocystis piceae*, was Δ^6^-protoilludene (**7**) which is a known natural product previously reported from two strains of Basidiomycota [100]. 

**Figure 23:**
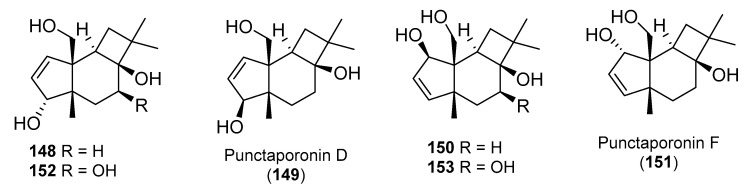
Structures of punctaporonins **148**–**153** isolated from the *Poronia* and *Pestalotiopsis* genera of fungi.

Isomeric allylic alcohols, punctaporonins A (**148**), D–F (**149**–**151**) (also known as punctatins), were isolated from the dung fungus *Poronia punctata* (Figure 23) [101,102,103]. The structures and absolute configurations of punctaporonins A, D and E (**148**–**150**) were solved by X-ray crystallographic analyses of the natural products or in the case of **150**, on an acetonide derivative. For punctaporonin F (**151**), a combination of derivatisation and NMR spectroscopic data comparison with the other punctaporonins was required to assign the structure, while the relative stereochemistry was assigned by biogenetic consideration. Only punctaporonins D (**149**) and E (**150**) have been shown to exhibit any bioactivity. In addition, **149** was shown to inhibit a mycelial form of *Candida albicans* at 1 ppm and **150** inhibited *Trichomonas vaginalis* at 100 ppm *in vitro* [101,102,103]. The enantiospecific syntheses of both punctaporonins A (**148**) and D (**149**) have been reported [104].

Two protoilludanes, 6-hydroxypunctaporonin A (**152**) and E (**153**) (Figure 23), were isolated from the plant pathogen *Pestalotiopsis disseminate* [105]. The absolute configuration of **153** was resolved by X-ray crystallographic analysis of a mono-bromobenzoate derivative, while that of **152** was assigned by consideration of shared biogenetic origins. Both **152** and **153** were investigated for their antimicrobial activities against *B. subtilis* ATCC6051, *S. aureus* ATCC29213, *E. coli* ATCC25922 and *C. albicans* ATCC14053 and **153** was found to exhibit weak activity against the two Gram-positive bacterial strains in disk diffusion assays at a loading of 100 µg per disk [105]. In addition, both compounds **152** and **153** were found to be non-cytotoxic against HeLa cells with IC_50_ >100 µM [106].

**Figure 24:**
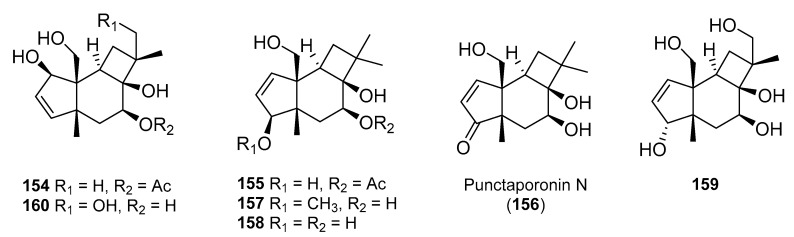
Structures of punctaporonins **154**–**160** isolated from the *Hansfordia* and *Pestalotiopsis* genera of fungi.

Punctaporonins L (**154**) and M (**155**) (Figure 24) were isolated from the sponge-derived fungus, *Hansfordia sinuosae* [107]. The relative configurations of both natural products were determined by analysis of NOESY spectroscopic data with the absolute stereochemistry of **154** assigned by comparison of optical rotations with **153**. The cytotoxicity and antibacterial activity of punctaporonins L (**154**) and M (**155**) were investigated. Both **154** and **155** exhibited no antibacterial activity (MIC >125 µM) against *E. coli*, *S. aureus*, *Bacillus thuringensis* or *B. subtilis* and were weakly cytotoxic (IC_50_ >10 µM) against HCT-8, Bel7402, BGC823, A549, and A2780 cell lines [107].

Five protoilludanes, punctaporonins N (**156**) and O (**157**), 6-hydroxypunctaporonin D (**158**), 6,13-dihydroxypunctaporonin A (**159**) and 6,13-dihydroxypunctaporonin E (**160**) (Figure 24) were isolated from another species in the *Pestalotiopsis* genus, *Pestalotiopsis* sp. [108]. NOESY spectroscopic analysis led to assignment of relative configurations of all the natural products except for punctaporonin N (**156**). The relative and absolute configurations of **156** were assigned based on biosynthetic consideration upon successful conversion of 6-hydroxypunctaporonin A (**152**) to **156**. The absolute configurations of **158** and **159** were ascertained by consideration of shared biogenetic origins with **152**. Subsequently, the absolute configurations of **157** and **160** were assigned on biogenetic consideration of **158** and **153**, respectively. All five compounds **156**–**160** were investigated for antimicrobial activity against *A. flavus, Fusarium verticillioides, S. aureus*, *B. subtilis*, *E. coli* and *C. albicans* at a loading of 100 µg per disk. However, none of the compounds were found active [108].

**Figure 25:**
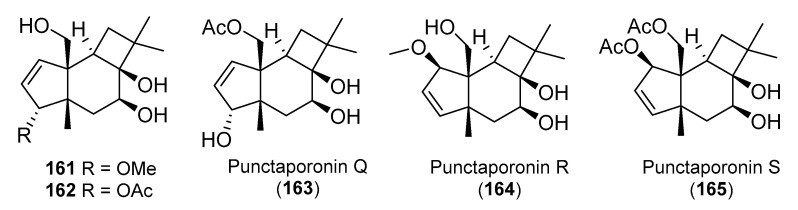
Structures of punctaporonins O–S (**161**–**165**) isolated from *Cytospora* sp.

A further five protoilludanes, punctaporonins O–S (**161**–**165**), were isolated from the plant pathogen, *Cytospora* sp. (Figure 25) [106]. Determination of the absolute configuration of punctaporonin O (**161**) was achieved by comparison of optical rotations with 6-hydroxypunctaporonin A (**152**) which led to assignment of the remaining natural products by consideration of shared biogenetic origins. The cytotoxicity of puctaporonins O–S (**161**–**165**) were investigated against HeLa cells, where **161**, **164** and **165** exhibited moderate cytotoxicity (IC_50_ 16.6, 10.4 and 47.4 µM, respectively), while **162** and **163** were non-cytotoxic (IC_50_ >100 µM) [106].

**Figure 26:**
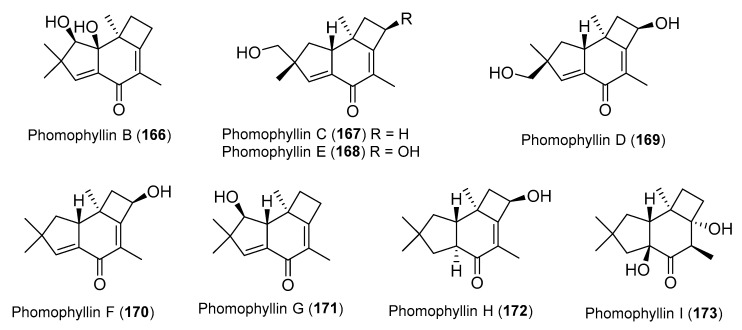
Structures of phomophyllins B–I (**166**–**173**) isolated from *Phomopsis* sp.

The plant-derived fungus, *Phomopsis* sp., afforded eight protoilludanes named phomophyllin B–I (**166**–**173**) (Figure 26) [109]. Assignment of the relative configurations of all phomophyllins **166**–**173** was achieved by NOESY spectroscopic analysis. X-ray crystallographic analysis of **166** and **173** allowed assignment of the absolute configurations of these two natural products. A modified Mosher’s method was utilised to establish the absolute configurations of **170** and **171**, while the absolute configurations of the remaining phomophyllins (**167**–**169**, **172**) were assigned by comparison of experimental ECD spectra with TDDFT calculated ECD spectra. All the phomophyllins (**166**–**173**), except for **172**, were investigated for inhibition of β-site amyloid precursor protein-cleaving enzyme 1 (BACE1) which is a target for Alzheimer’s disease. They exhibited inhibition rates ranging between 19–40% at a concentration of 40 µM, while exhibiting no hepatoxicity to L-02 liver cells at the same concentration [109].

## 4. Protoilludanes from Other Sources

**Figure 27:**
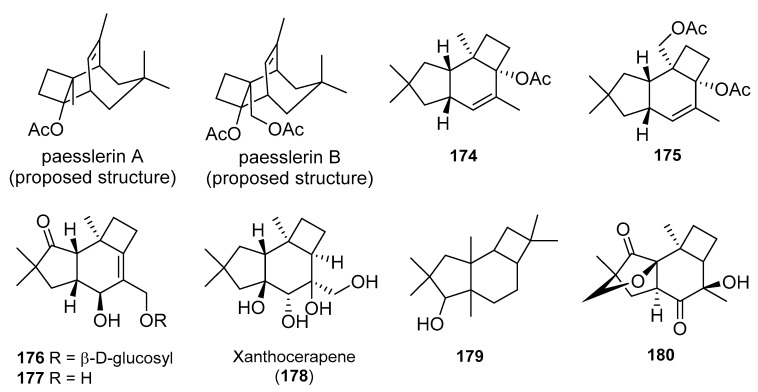
Structures of protoilludanes **174**–**180** isolated from marine and plant sources.

A few examples of protoilludanes have been isolated from sources other than fungi (Figure 27). Two marine natural products, paesslerins A (**174**) and B (**175**) were isolated from the soft coral *Alcyonium paessleri* [110]. The relative configurations of **174** and **175** were assigned by NOESY spectroscopic analysis [110]. Both natural products exhibited moderate cytotoxicity in preliminary studies [110]. A stereoselective synthesis for the proposed structure of **174** was reported a few years after its isolation. However, the spectroscopic data was not in agreement resulting in the conclusion that a structure correction was required [111]. More recently, another total synthesis of the suspected structure of **174** was reported for which the spectroscopic data was in agreement with the natural product resulting in revision of the natural product structure [112].

The first example of a protoilludane sesquiterpene glucoside, pteridanoside (**176**), was isolated from the bracken fern *Pteridium aquilinum* var. *caudatum* along with its aglycon pteridanone (**177**) [113]. Determination of the relative configuration of **177** was achieved utilising NOESY spectroscopic data. The absolute configuration of pteridanone was determined by a combination of ECD spectral analysis and conformational calculations upon conversion of **177** to **176**. Pteridanoside (**176**), unlike its aglycon **177**, exhibited weak toxicity against brine shrimp, *Artemia salina* (LC_50_ = 250 and 62.5 µg/mL, 24 h and 48 h) [113]. 

Xanthocerapene (**178**) was isolated from the wood of *Xanthoceras sorbifolia* with assignment of the relative stereochemistry of the natural product achieved utilising NOESY spectroscopic data [114]. Another protoilludane from a plant source, 2,2,4a,7a-tetramethyldecahydro-1*H*-cyclobuta[*e*]inden-5-ol (**179**), was first detected by gas chromatography mass spectrometry (GC–MS) from 4-year-old *Radix et Rhizoma ginseng* [115]. More recently, it has also been detected by GC–MS from other species of ginseng. However, the relative stereochemistry of the natural product remains unknown [116,117,118]. Lastly, (3*S*,4a*S*,6*R*,8a*S*,8b*R*)-6-hydroxy-3,6,8a-trimethyloctahydro-3,8*b*-methanocyclobuta[*h*]chromene-5,9(6*H*)-dione (**180**) was isolated from the roots of *Lindera strychnifolia* [119]. The relative stereochemistry of **180** was assigned by analysis of NOESY data, while the absolute configuration was assigned upon comparison of experimental ECD data with calculated data. The natural product has been reported to improve the cell viability of human umbilical vein endothelial cells injured by oxidised low-density lipoprotein [119].

**Figure 28:**
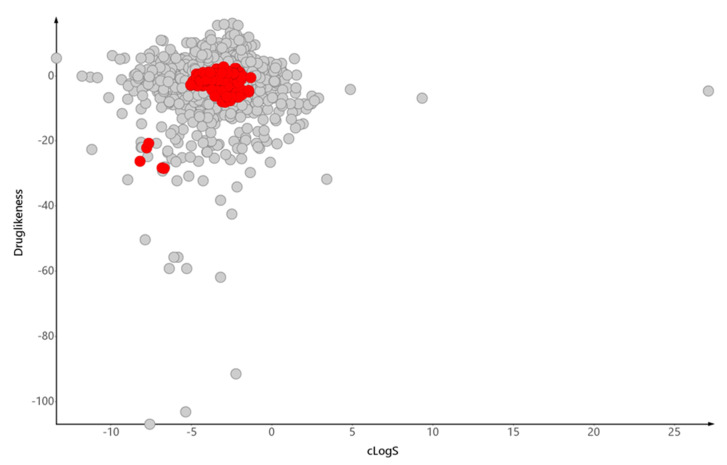
Comparison of calculated descriptors from Datawarrior for protoilludanes (red) with current FDA-approved drugs (grey).

All 180 protoilludanes were analysed on Datawarrior [120] to calculate descriptors such as lipophilicity (cLogP), aqueous solubility (cLogS) and druglikeness. A total of 173 compounds showed cLogP values of less than 5, which is considered ideal for good absorption across cell membranes, while 139 were considered to have good solubility with cLogS values greater than -4, which are values that more than 80% of the current drugs on the market have (Figure 28). Lastly the compounds were evaluated for their druglikeness which is a descriptor indicating the frequency of occurrence of fragments found in commercial drugs where a positive value states higher amounts of these fragments in the compound. Only 64 protoilludanes showed positive values for the druglikeness ranging from 0 to 2.8. Overall, 51 protoilludanes were found to have good lipophilicity, aqueous solubility and druglikeness properties. 

## 5. Biosynthetic Studies

**Figure 29:**
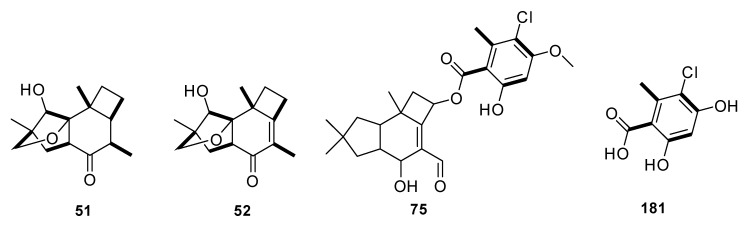
Structures ^13^C labelled protoilludanes, illustrating the incorporation of intact acetate units (bold).

The biosynthesis of two protoilludane natural products, coprinolone (**51**) and Δ^6^-coprinolone (**52**), has been investigated using sodium [1,2–^13^C] acetate labelling experiments [56,57]. In both instances, successful incorporation of [1,2–^13^C] acetate was confirmed by the presence of enriched carbon signals in the ^13^C NMR spectrum. Subsequently, ^13^C–^13^C connectivities from a ^13^C-COSY experiment identified the intact acetate units (Figure 29) which were in accordance with the labelling pattern expected for compounds derived from farnesyl diphosphate (Scheme 1) [56,57]. A similar feeding experiment was conducted on *Armillaria mellea*, to investigate the origin of the orsellinic acid moiety in arnamial (**75**) using sodium [1,2–^13^C] acetate [68]. Successful incorporation of [1,2–^13^C] acetate was observed for orsellinic acid (**181**), a common biosynthetic intermediate in *Armillaria*, with ^13^C-COSY couplings identifying intact acetate units. This experiment supported the hypothesis that orsellinic acid (**181**) and in turn arnamial (**75**) were of polyketide origin [68].

## 6. Conclusions

**Figure 30:**
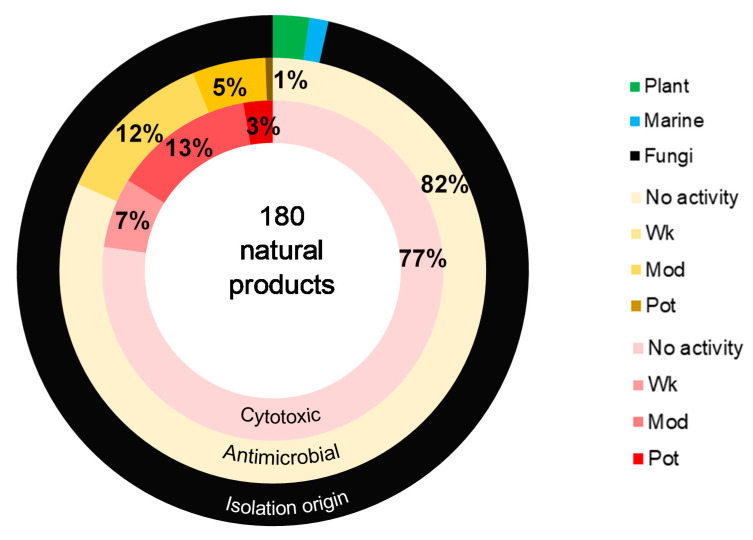
Summary of protoilludanes isolated to date with their bioactivity.

Fungi are a prolific source of sesquiterpenoid protoilludane natural products, which bear a unique 5/6/4 fused-ring system, with 96% of described compounds isolated from higher fungi of which Basidiomycota was the major producer. The total synthesis of only 16 protoilludanes has been undertaken so far, where two were of the non-natural enantiomer, due to the structural complexity of the tricyclic core. Overall, 78% of the isolated protoilludanes have been investigated for their bioactivity, predominantly for antimicrobial and/or cytotoxic properties (89%). Of the 180 examples reported so far (Table S1), 18% were identified as antimicrobials, of which 6% exhibited modest to potent activity, against a range of bacterial and fungal pathogens, while 23% were identified as cytotoxic, with 16% exhibiting modest to potent cytotoxicity, against human cancer cell lines (Figure 30). The diversity of chemical structures in the protoilludane class of natural products combined with their interesting bioactivities make this scaffold worthy of further exploration.

## Supplementary Materials

The following are available online at https://www.mdpi.com/2079-6382/9/12/928/s1, Table S1: A review of fungal protoilludane sesquiterpenoid natural products.

## References

[1] Blackwell M. (2011). The Fungi: 1, 2, 3 … 5.1 million species?. Am. J. Bot..

[2] Zhong J.-J., Bai F.-W., Zhang W. (2009). Biotechnology in China I.

[3] Schrader J., Bohlmann J. (2015). Biotechnology of Isoprenoids.

[4] Alves M.J., Ferreira I.C.F.R., Dias J., Teixeira V., Martins A., Pintado M. (2012). A Review on Antimicrobial Activity of Mushroom (Basidiomycetes) Extracts and Isolated Compounds. Planta Med..

[5] Hibbett D.S., Binder M., Bischoff J.F., Blackwell M., Cannon P.F., Eriksson O.E., Huhndorf S., James T., Kirk P.M., Lücking R. (2007). A higher-level phylogenetic classification of the Fungi. Mycol. Res..

[6] Bass D., Richards T.A. (2011). Three reasons to re-evaluate fungal diversity ‘on Earth and in the ocean’. Fungal Biol. Rev..

[7] Hans J.R. (2003). Natural Products: The Secondary Metabolites.

[8] Mann J. (1994). Chemical Aspects of Biosynthesis; Oxford Chemistry Primers.

[9] Dewick P.M. (2002). Medicinal Natural Products: A Biosynthetic Approach.

[10] Dewick P.M. (2002). The biosynthesis of C5–C25 terpenoid compounds. Nat. Prod. Rep..

[11] Tibrewal N., Tang Y. (2014). Biocatalysts for Natural Product Biosynthesis. Annu. Rev. Chem. Biomol. Eng..

[12] Quin M.B., Flynn C.M., Schmidt-Dannert C. (2014). Traversing the fungal terpenome. Nat. Prod. Rep..

[13] Nord C.L., Menkis A., Broberg A. (2015). Cytotoxic Illudane Sesquiterpenes from the Fungus *Granulobasidium vellereum* (Ellis and Cragin) Jülich. J. Nat. Prod..

[14] Siengalewicz P., Mulzer J., Rinner U. (2011). Synthesis of Protoilludanes and Related Sesquiterpenes. Eur. J. Org. Chem..

[15] Burgess M.L., Barrow K.D. (1999). Biosynthesis of illudosin, a fomannosane-type sesquiterpene, by the Basidiomycete *Omphalotus nidiformis*. J. Chem. Soc. Perkin Trans. 1.

[16] Anchel M., Hervey A., Robbins W.J. (1950). Antibiotic Substances from Basidiomycetes: VII. *Clitocybe Illudens*. Proc. Natl. Acad. Sci. USA.

[17] McMorris T.C., Nair M.S.R., Anchel M. (1967). Structure of illudol, a sesquiterpenoid triol from *Clitocybe illudens*. J. Am. Chem. Soc..

[18] McMorris T.C., Nair M.S.R., Singh P., Anchel M. (1971). The Structure of Illudol. Phytochemistry.

[19] Matsumoto T., Miyano K., Kagawa S., Yi S., Ogawa J., Ichihara A. (1971). Total Synthesis of dl-illudol. Tet. Lett..

[20] Nair M.S.R., Anchel M. (1975). Metabolic Products of *Clitocybe Illudens* XI. The Structure of Neoilludol. Tet. Lett..

[21] Arnone A., Cardillo R., Modugno V.D., Nasini G. (1989). Secondary Mould Metabolites. Part 29.’ Isolation and Structure Elucidation of Candicansol, 3-epi-Illudol and 1-O-Acetyl-3-epi-illudol, Novel Sesquiterpenoids from *Clitocybe candicans*, and Absolute Configuration of 3-epi-llludol. J. Chem. Soc. Perkin Trans. 1.

[22] Suzuki S., Murayama T., Shiono Y. (2006). Echinolactones C and D: Two Illudalane Sesquiterpenoids Isolated from the Cultured Mycelia of the Fungus *Echinodontium japonicum*. Z. Naturforsch..

[23] Suzuki S., Murayama T., Shiono Y. (2005). Illudalane sesquiterpenoids, echinolactones A and B, from a mycelial culture of *Echinodontium japonicum*. Phytochemistry.

[24] Elliott M.R., Dhimane A.-L., Malacria M. (1997). Biomimetic Diastereoselective Total Synthesis of *epi*-Illudol *via* a Transannular Radical Cyclizations Strategy. J. Am. Chem. Soc..

[25] McMorris T.C., Kashinatham A., Lira R., Rundgren H., Gantzel P.K., Kelner M.J., Dawe R. (2002). Sesquiterpenes from *Omphalotus illudens*. Phytochemistry.

[26] Yang X.-Y., Li Z.-H., Dong Z.-J., Feng T., Liu J.-K. (2014). Three new sesquiterpenoids from cultures of the basidiomycete *Conocybe siliginea*. J. Asian Nat. Prod. Res..

[27] Nozoe S., Kobayashi H., Urano S., Furukawa J. (1977). Isolation of Δ6-protoilludene and the related alcohols. Tetrahedron Lett..

[28] Furukawa J., Morisaki N., Kobayashi H., Iwasaki S., Nozoe S., Okuda S. (1985). Synthesis of dl-6-protoilludene. Chem. Pharm. Bull..

[29] Takeshita H., Iwabuchi H., Kouno I., Iino M., Nomura D. (1979). Photochemical construction of the protoilludane skeleton: A total synthesis of protoillud-7-ene and some oxygenated derivatives. Chem. Lett..

[30] Rabe P., Rinkel J., Nubbemeyer B., Köllner T.G., Chen F., Dickschat J.S. (2016). Terpene Cyclases from Social Amoebae. Angew. Chem. Int. Ed..

[31] Sasaki H., Kubohara Y., Ishigaki H., Takahashi K., Eguchi H., Sugawara A., Oshima Y., Kikuchi H. (2020). Two New Terpenes Isolated from *Dictyostelium* Cellular Slime Molds. Molecules.

[32] Arnone A., Nasini G., Assante G., Roeijmans H.J., Van Euk G.W. (1987). Sulcatine, a norsesquiterpene from the fungus *Laurilia sulcata*. Phytochemistry.

[33] Arnone A., Nasini G., Assante G., Eijk G.W. (1992). Three sesquiterpenes produced by the fungus *Laurilia sulcata*. Phytochemistry.

[34] Assante G., Dallavalle S., Martino P.A. (2012). Protoilludane sesquiterpenoids as scaffold structures for new antimicrobials against *Mannheimia haemolytica*. J. Antibiot..

[35] Arnone A., Brambilla U., Nasini G., De Pava O.V. (1995). Isolation and structure elucidation of tsugicolines A-D, novel protoilludane sesquiterpenes from *Laurilia tsugicola*. Tetrahedron.

[36] Arnone A., De Gregorio C., Meille S.V., Nasini G., Sidoti G. (1999). Tsugicoline E, a New Polyoxygenated Protoilludane Sesquiterpene from the Fungus *Laurilia tsugicola*. J. Nat. Prod..

[37] Zheng Y., Shen Y. (2009). Clavicorolides A and B, Sesquiterpenoids from the Fermentation Products of Edible Fungus *Clavicorona pyxidata*. Org. Lett..

[38] Zheng Y.-B., Lu C.-H., Zheng Z.-H., Lin X.-J., Su W.-J., Shen Y.-M. (2008). New Sesquiterpenes from Edible Fungus *Clavicorona pyxidata*. Helv. Chim. Acta.

[39] Clericuzio M., Fu J., Pan F., Pang Z., Sterner O. (1997). Structure and absolute configuration of protoilludane sesquiterpenes from *Russula delica*. Tetrahedron.

[40] Clericuzio M., Pan F., Han F., Pang Z., Sterner O. (1997). Stearoyldelicone, an unstable protoilludane sesquiterpenoid from intact fruit bodies of *Russula delica*. Tetrahedron Lett..

[41] Clericuzio M., Han F., Pan F., Pang Z., Sterner O. (1998). The Sesquiterpenoid Contents of Fruit Bodies of *Russula delica*. Acta Chem. Scand..

[42] Yoshikawa K., Kaneko A., Matsumoto Y., Hama H., Arihara S. (2006). Russujaponols A−F, Illudoid Sesquiterpenes from the Fruiting Body of *Russula japonica*. J. Nat. Prod..

[43] Clericuzio M., Mella M., Toma L., Finzi P.V., Vidari G. (2002). Atlanticones, New Protoilludane Sesquiterpenes from the Mushroom *Lactarius atlanticus* (Basidiomycetes). Eur. J. Org. Chem..

[44] Zech M.S.A., Jandl C., Bach T. (2019). Concise Access to the Skeleton of Protoilludane Sesquiterpenes through a Photochemical Reaction Cascade: Total Synthesis of Atlanticone C. Angew. Chem. Int. Ed..

[45] Bach T., Proessdorf J., Zech A., Jandl C. (2020). Concise Total Synthesis of (+)-Atlanticone C. Synlett.

[46] Hirota M., Shimizu Y., Kamo T., Makabe H., Shibata H. (2003). New Plant Growth Promoters, Repraesentins A, B and C, from *Lactarius repraesentaneus*. Biosci. Biotechnol. Biochem..

[47] Takeuchi T., Iinuma H., Momose I., Matsui S. (2002). Antibiotic Pasteurestin A and B and their Manufacture with *Agrocybe cylindracea*. JP Patent.

[48] Surup F., Hennicke F., Sella N., Stroot M., Bernecker S., Pfütze S., Stadler M., Rühl M. (2019). New terpenoids from the fermentation broth of the edible mushroom Cyclocybe aegerita. Beilstein J. Org. Chem..

[49] Kögl M., Brecker L., Warrass R., Mulzer J. (2007). Total Synthesis and Configurational Assignment of Pasteurestin A and B. Angew. Chem. Int. Ed..

[50] Kögl M., Brecker L., Warrass R., Mulzer J. (2008). Novel Protoilludane Lead Structure for Veterinary Antibiotics: Total Synthesis of Pasteurestins A and B and Assignment of Their Configurations. Eur. J. Org. Chem..

[51] Li H.-T., Tang L.-H., Liu T., Yang R.-N., Yang Y.-B., Zhou H., Ding Z.-T. (2020). Protoilludane-type sesquiterpenoids from *Armillaria* sp. by co-culture with the endophytic fungus *Epicoccum* sp. associated with *Gastrodia elata*. Bioorganic Chem..

[52] Stärk A., Anke T., Mocek U., Steglich W., Kirfel A., Will G. (1988). Lentinellic Acid, a Biologically Active Protoilludane Derivative from *Lentinellus* Species (Basidiomycetes). Z. Naturforsch. C.

[53] Wunder A., Anke T., Klostermeyer D., Steglich W. (1996). Lactarane Type Sesquiterpenoids as Inhibitors of Leukotriene Biosynthesis and Other, New Metabolites from Submerged Cultures of *Lentinellus cochleatus* (Pers. ex Fr.) Karst. Z. Naturforsch. C.

[54] Fabian K., Lorenzen K., Anke T., Johansson M., Sterner O. (1999). Five new bioactive sesquiterpenes from the fungus *Radulomyces confluens* (Fr.) Christ. Z. Naturforsch. C.

[55] Chang E.L., Bolte B., Lan P., Willis A.C., Banwell M.G. (2016). Chemoenzymatic Total Syntheses of the Enantiomers of the Protoilludanes 8-Deoxydihydrotsugicoline and Radudiol. J. Org. Chem..

[56] Starratt A.N., Stothers J.B., Ward E.W.B. (1988). Coprinolone, an oxygen-bridged protoilludane from the fungus *Coprinus psychromorbidus*: Structure determination by chemical and n.m.r. studies aided by biosynthetic incorporation of [1,2-13C2]acetate. J. Chem. Soc. Chem. Commun..

[57] Starratt A.N., Ward E.W.B., Stothers J.B. (1989). Coprinolone and Δ6-coprinolone: New sesquiterpenes from *Coprinus psychromorbidus*. Can. J. Chem..

[58] Nord C.L., Menkis A., Vasaitis R., Broberg A. (2013). Protoilludane sesquiterpenes from the wood decomposing fungus *Granulobasidium vellereum* (Ellis & Cragin) Jülich. Phytochemistry.

[59] Nord C.L., Menkis A., Lendel C., Vasaitis R., Broberg A. (2014). Sesquiterpenes from the saprotrophic fungus *Granulobasidium vellereum* (Ellis & Cragin) Jülich. Phytochemistry.

[60] Shiono Y., Seto T., Kamata M., Takita C., Suzuki S., Murayama T., Ikeda M. (2004). Protoilludane-Type Sesquiterpenes, Echinocidins A and B, from a Mycelial Culture of *Echinodontium tsugicola*. Z. Naturforsch. B.

[61] Shiono Y., Suzuki S., Murayama T., Ikeda M., Abe Y. (2005). Protoilludane Sequiterpenoids, Echinocidins C and D Produced by a Decay Causing Fungal Strain *Echinodontium tsugicola*. Z. Naturforsch. B.

[62] Shimoda K., Yamaoka Y., Yoo D., Yamada K.-I., Takikawa H., Takasu K. (2019). Total Syntheses of Allelopathic 4-Oxyprotoilludanes, Melleolides, and Echinocidins. J. Org. Chem..

[63] Donnelly D., Sanada S., O’Reilly J., Polonky J., Prangé T., Pascard C. (1982). Isolation and structure (X-ray analysis) of the orsellinate of armillol, a new antibacterial metabolite from *Armillaria mellea*. J. Chem. Soc. Chem. Commun..

[64] Donnelly D.M.X., Abe F., Coveney D., Fukuda N., O’Reilly J., Polonsky J., Prangé T. (1985). Antibacterial Sesquiterpene Aryl Esters from *Armillaria mellea*. J. Nat. Prod..

[65] Donnelly D.M.X., Coveney D.J., Fukuda N., Polonsky J. (1986). New Sesquiterpene Aryl Esters from *Armillaria mellea*. J. Nat. Prod..

[66] Yang J.-S., Cong P.-Z. (1988). Mass Spectrometric Studies on the Sesquiterpenol Aromatic Esters from Mycelium of *Armillaria Mellea*. Huaxue Xuebao.

[67] Yang J.S., Chen Y.W., Feng X.Z., Yu D.Q., He C.H., Zheng Q.T., Liang X.T. (2007). Isolation and Structure Elucidation of Armillaricin. Planta Med..

[68] Misiek M., Williams J., Schmich K., Hüttel W., Merfort I., Salomon C.E., Aldrich C.C., Hoffmeister D. (2009). Structure and Cytotoxicity of Arnamial and Related Fungal Sesquiterpene Aryl Esters. J. Nat. Prod..

[69] Bohnert M., Miethbauer S., Dahse H.-M., Ziemen J., Nett M., Hoffmeister D. (2011). In vitro cytotoxicity of melleolide antibiotics: Structural and mechanistic aspects. Bioorganic Med. Chem. Lett..

[70] Bohnert M., Nuetzmann H.-W., Eschroeckh V., Horn F., Dahse H.-M., Brakhage A.A., Hoffmeister D. (2014). Cytotoxic and antifungal activities of melleolide antibiotics follow dissimilar structure–activity relationships. Phytochemistry.

[71] Donnelly D.M.X., Polonsky J., Prangé T., Snatzke G., Wagner U. (1984). The absolute configuration of the orsellinate of armillol; application of the coupled oscillator theory. J. Chem. Soc. Chem. Commun..

[72] Cremin P., Donnelly D.M.X., Guiry P.J., Wolfender J.-L., Hostettmann K. (2000). A liquid chromatography–thermospray ionisation–mass spectrometry guided isolation of a new sesquiterpene aryl ester from *Armillaria novae-zelandiae*. J. Chem. Soc. Perkin Trans. 1.

[73] Misiek M., Hoffmeister D. (2010). Sesquiterpene aryl ester natural products in North American *Armillaria* species. Mycol. Prog..

[74] Chen C.-C., Kuo Y.-H., Cheng J.-J., Sung P.-J., Ni C.-L., Chen C.-C., Shen C.-C. (2015). Three New Sesquiterpene Aryl Esters from the Mycelium of *Armillaria mellea*. Molecules.

[75] Midland S.L., Izac R.R., Wing R.M., Zaki A.I., Munnecke D.E., Sims J.J. (1982). Melleolide, A new Antibiotic from *Armillaria mellea*. Tet. Lett..

[76] Arnone A., Cardillo R., Nasini G. (1986). Structures of melleolides B-D, three antibacterial sesquiterpenoids from *Armillaria mellea*. Phytochemistry.

[77] Arnone A., Cardillo R., Di Modugno V., Nasini G. (1988). Secondary mould metabolites. XXII. Isolation and structure elucidation of melledonals D and E and melleolides E-H, novel sesquiterpenoid aryl esters from *Clitocybe elegans* and *Armillaria mellea*. Gazz. Chim. Ital..

[78] Momose I., Sekizawa R., Hosokawa N., Iinuma H., Maisui S., Nakamura H., Naganawa H., Hamada M., Takeuchi T. (2000). Melleolides K, L and M, New Melleolides from *Armillariella mellea*. J. Antibiot..

[79] Chen C.-C., Cheng J.-J., Shen C.-C. (2011). Protoilludane Norsesquiterpenoid Esters and Uses Thereof. U.S. Patent.

[80] Arnone A., Cardillo R., Nasini G. (1988). Secondary mould metabolites. XXIII. Isolation and structure elucidation of melleolides I and J and armellides A and B, novel sesquiterpenoid aryl esters from *Armillaria novae-zelandiae*. Gazz. Chim. Ital..

[81] Yang J., Su Y., Wang Y., Feng X., Yu D., Cong P., Tamai M., Obuchi T., Kondoh H., Liang X. (1989). Isolation and Structures of Two New Sesquiterpenoid Aromatic Esters: Armillarigin and Armillarikin1. Planta Medica.

[82] Arnone A., Cardillo R., Nasini G., Meille S.V. (1988). Secondary mould metabolites. Part 19. Structure elucidation and absolute configuration of melledonals B and C, novel antibacterial sesquiterpenoids from *Armillaria mellea*. X-Ray molecular structure of melledonal C. J. Chem. Soc. Perkin Trans. 1.

[83] Kobori H., Sekiya A., Suzuki T., Choi J.-H., Hirai H., Kawagishi H. (2015). Bioactive Sesquiterpene Aryl Esters from the Culture Broth of *Armillaria* sp.. J. Nat. Prod..

[84] Li Z., Wang Y., Jiang B., Li W., Zheng L., Yang X., Bao Y., Sun L., Huang Y.-X., Li Y. (2016). Structure, cytotoxic activity and mechanism of protoilludane sesquiterpene aryl esters from the mycelium of *Armillaria mellea*. J. Ethnopharmacol..

[85] Yin X., Feng T., Liu J.-K. (2012). Structures and cytotoxicities of three new sesquiterpenes from cultures of *Armillaria* sp.. Nat. Prod. Bioprospect..

[86] Cremin P., Donnelly D.M., Wolfender J.-L., Hostettmann K. (1995). Liquid chromatographic-thermospray mass spectrometric analysis of sesquiterpenes of *Armillaria* (Eumycota: Basidiomycotina) species. J. Chromatogr. A.

[87] Donnelly D.M., Hutchinson R.M., Coveney D., Yonemitsu M. (1990). Sesquiterpene aryl esters from *Armillaria mellea*. Phytochemistry.

[88] Donnelly D.M., Konishi T., Dunne O., Cremin P. (1997). Sesquiterpene aryl esters from *Armillaria tabescens*. Phytochemistry.

[89] Donnelly D.M., Coveney D.J., Polonsky J. (1985). Melledonal and melledonol, sesquiterpene esters from *Armillaria mellea*. Tetrahedron Lett..

[90] Donnelly D.M., Quigley P.F., Coveney D.J., Polonsky J. (1987). Two new sesquiterpene esters from *Armillaria mellea*. Phytochemistry.

[91] Yang J., Yuwu C., Xiaozhang F., Dequan Y., XiaoTian L. (1984). Chemical Constituents of *Armillaria mellea* Mycelium I. Isolation and Characterization of Armillarin and Armillaridin. Planta Medica.

[92] Yang J.S., Su Y.L., Wang Y.L., Feng X.Z., Yu D.Q., Liang X.T. (1990). Studies on the chemical constituents of *Armillaria mellea* mycelium. V. Isolation and characterization of armillarilin and armillarinin. Yao Xue Xue Bao.

[93] Yang J.S., Su Y.L., Wang Y.L., Feng X.Z., Yu D.Q., Liang X.T., He C.H., Zheng Q.T. (1990). Chemical constituents of *Armillaria mellea* mycelium. VI. Isolation and structure of armillaripin. Yao Xue Xue Bao.

[94] Yang J.S., Su Y.L., Wang Y.L., Feng X.Z., Yu D.Q., Liang X.T. (1991). Chemical constituents of *Armillaria mellea* mycelium. VII. Isolation and characterization of chemical constituents of the acetone extract. Yao Xue Xue Bao.

[95] Yang J., Su Y., Wang Y., Feng X., Yu D., Liang X. (1991). Two Novel Protoilludane Norsesquiterpenoid Esters, Armillasin and Armillatin, from *Armillaria mellea*. Planta Med..

[96] Yang J.S., Watube S. (1989). Antibiotic Armillaric Acid and Its Manufacture with *Armillaria mellea*. CN Patent.

[97] Donnelly D.M., Hutchinson R.M. (1990). Armillane, a saturated sesquiterpene ester from *Armillaria mellea*. Phytochemistry.

[98] Obuchi T., Kondoh H., Watanabe N., Tamai M., Imura S., Jun-Shan Y., Xiao-Tian L. (1990). Armillaric Acid, A New Antibiotic Produced by *Armillaria mellea*. Planta Med..

[99] Sonnenbichler J., Guillaumin J.-J., Peipp H., Schwarz D. (1997). Secondary metabolites from dual cultures of genetically different **Armillaria** isolates. For. Pathol..

[100] Hanssen H.-P., Sprecher E., Abraham W.-R. (1986). 6-Protoilludene, the major volatile metabolite from *ceratocystis piceae* liquid cultures. Phytochemistry.

[101] Anderson J.R., Briant C.E., Edwards R.L., Mabelis R.P., Poyser J.P., Spencer H., Whalley A.J.S. (1984). Punctatin A (antibiotic M95464): X-ray crystal structure of a sesquiterpene alcohol with a new carbon skeleton from the fungus, *Paronia punctata*. J. Chem. Soc., Chem. Commun..

[102] Poyser J.P., Edwards R.L., Anderson J.R., Hursthouse M.B., Walker N.P.C., Sheldrick G.M., Whalley A.J.S. (1986). Punctatins A,D,E, and F (antibiotics M95464, M167906, M171950, and M189122), isomeric allylic alcohols from the fungus *Poronia punctata*: X-ray crystal structures of D and of E acetonide. J. Antibiot..

[103] Anderson J.R., Edwards R.L., Poyser J.P., Whalley A.J.S. (1988). Metabolites of the higher fungi. Part 23. The punctaporonins. Novel bi-, tri-, and tetra-cyclic sesquiterpenes related to caryophyllene, from the fungus *Poronia punctate* (Linnaeus:Fries) Fries. J. Chem. Soc. Perkin Trans. 1.

[104] Sugimura T., Paquette L.A. (1987). Enantiospecific total synthesis of the sesquiterpene antibiotics (-)-punctatin A and (+)-punctatin D. J. Am. Chem. Soc..

[105] Deyrup S.T., Swenson D.C., Gloer J.B., Wicklow D.T. (2006). Caryophyllene Sesquiterpenoids from a Fungicolous Isolate of *Pestalotiopsis disseminata*. J. Nat. Prod..

[106] Li Y., Wang Q., Liu X., Che Y. (2017). Punctaporonins N–S, New Caryophyllene Sesquiterpenoids from *Cytospora sp*. BioMed Res. Int..

[107] Wu Z.-H., Liu D., Proksch P., Guo P., Lin W. (2014). Punctaporonins H–M: Caryophyllene-Type Sesquiterpenoids from the Sponge-Associated Fungus *Hansfordia sinuosae*. Mar. Drugs.

[108] Hwang I.H., Wicklow D.T., Gloer J.B. (2016). New punctaporonins from two fungicolous isolates of *Pestalotiopsis* sp.. Phytochem. Lett..

[109] Xie S., Wu Y., Qiao Y., Guo Y., Wang J., Hu Z., Zhang Q., Li X., Huang J., Zhou Q. (2018). Protoilludane, Illudalane, and Botryane Sesquiterpenoids from the Endophytic Fungus *Phomopsis* sp. TJ507A. J. Nat. Prod..

[110] Brasco M.F.R., Seldes A.M., Palermo J.A. (2001). Paesslerins A and B: Novel Tricyclic Sesquiterpenoids from the Soft Coral *Alcyonium aessleri*. Org. Lett..

[111] Inanaga K., Takasu K., Ihara M. (2004). Rapid Assembly of Polycyclic Substances by a Multicomponent Cascade (4 + 2)−(2 + 2) Cycloadditions: Total Synthesis of the Proposed Structure of Paesslerin A. J. Am. Chem. Soc..

[112] Mogi Y., Inanaga K., Tokuyama H., Ihara M., Yamaoka Y., Yamada K.-I., Takasu K. (2019). Rapid Assembly of Protoilludane Skeleton through Tandem Catalysis: Total Synthesis of Paesslerin A and Its Structural Revision. Org. Lett..

[113] Castillo U.F., Sakagami Y., Alonso-Amelot M., Ojika M. (1999). Pteridanoside, the first protoilludane sesquiterpene glucoside as a toxic component of the neotropical bracken fern *Pteridium aquilinum var. caudatum*. Tetrahedron.

[114] Ma C.M., Nakamura N., Nawawi A., Hattori M., Cai S.Q. (2004). A Novel Protoilludane Sesquiterpene from the Wood of *Xanthoceras sorbifolia*. Chin. Chem. Lett..

[115] Wang J., Zhang L.-X., Zhao Y., Chen W.-X., Yang Q., Wang Y.-X. (2011). Volatile Oil Contents and Their Variation Law of RADIX ET RHIZOMA GINSENG in Changbai Mountain Areas. Med. Plant.

[116] Jiao H.-Y., Wang Y.-S., Mo X.-L., Zhang X.-T., Zeng Q.-Q. (2013). Analysis of volatile components of Pogostemon cablin from Indonesia and China. Jinri Yaoxue.

[117] Tong H.-F., Xue J., Tong Y.-L. (2013). Analysis of volatile components from ginseng and American ginseng for identification by GC-MS. Zhongyiyao Xuebao.

[118] Wang W., Liu X., Liu J., Cai E., Zhao Y., Li H., Zhang L., Li P., Gao Y. (2017). Sesquiterpenoids from the Root of Panax ginseng Attenuates Lipopolysaccharide-Induced Depressive-Like Behavior through the Brain-Derived Neurotrophic Factor/Tropomyosin-Related Kinase B and Sirtuin Type 1/Nuclear Factor-κB Signaling Pathways. J. Agric. Food Chem..

[119] Wu J. (2016). Protoilludane Sesquiterpenoid Compound, Preparation Method and Medical Applications Thereof. CN Patent.

[120] Sander T., Freyss J., Von Korff M., Rufener C. (2015). DataWarrior: An Open-Source Program for Chemistry Aware Data Visualization and Analysis. J. Chem. Inf. Model..

