# Supplementary material for: A Review of Fungal Protoilludane Sesquiterpenoid Natural Products
Source: Antibiotics (Basel). 2020 Dec 19;9(12):928. doi: 10.3390/antibiotics9120928 (PMC7765842; doi:10.3390/antibiotics9120928)
Supplement: Supplementary file 1 [file antibiotics-09-00928-s001.pdf]

Supporting Information

A review of fungal protoilludane sesquiterpenoid natural products.

Melissa M. Cadelis[1,2*], Brent R. Copp[1], Siouxsie Wiles[2]

[1] School of Chemical Sciences, University of Auckland, 23 Symonds Street, 1010 Auckland, New Zealand; m.cadelis@auckland.ac.nz, b.copp@auckland.ac.nz
[2] Bioluminescent Superbugs Lab, School of Medical Sciences, University of Auckland, 85 Park Road, Grafton, 1023 Auckland, New Zealand; s.wiles@auckland.ac.nz

* Correspondence: m.cadelis@auckland.ac.nz

Name	Structure	Isolation origin	Bioactivity	Stereochemistry	Synthesis
			**Basidiomycota**		
Illudol **(1)**	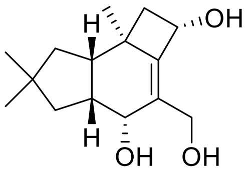	*Clitocybe illudens*, **1950** [1,2]	No antimicrobial activity against *B. mycoides*, *B. subtilis*, *E. coli*, *K. pneumoniae*, *M. smegmatis*, *M. tuberculosis*, *P. aeruginosa* and *S. aureus* [1].	**1971**- Absolute established on basis of X-ray on illudol derivative [3].	**1971** – racemic [4].
Neoilludol **(2)**	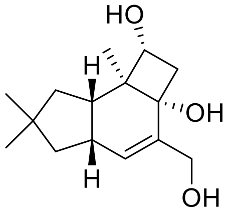	*Clitocybe illudens*, **1975** [5]	No antimicrobial activity against *S. aureus* NBRC 13276 and *P. aeruginosa* ATCC 15442 at 100 µg [5].	**2006**- Relative by NOESY [6].	
3-*epi*-Illudol **(3)**	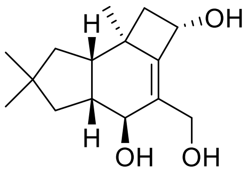	*Clitocybe candicans*, **1989** [7]	Antimicrobial activity against *E. coli* ATCC25922 MIC 32 µg/mL [8]. Inactive against *P. multocida* ATCC15743 and *M. haemolytica* ATCC14003 [9].	**1989**- Relative by NOESY. Absolute established by partial resolution method of Horeau on an acetonide derivative and ECD on 1-*O*-pivaloyl-3,6-dinitrobenzoyl derivative [7].	**1997** – racemic [10]
1-*O*-Acetyl-3-*epi*-illudol **(4)**	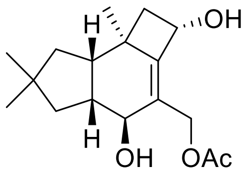	*Clitocybe candicans*, **1989** [7]		**1989**- Relative established by biogenetic consideration with 3-*epi*-illudol [7].	

Illudiolone **(5)**	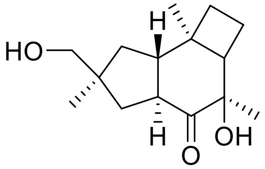	*Omphalotus illudens*, **2002** [11]		**2002**- X-ray [11].	
5-*O*-Acetyl-7,14-dihydroxy-protoilludanol **(6)**	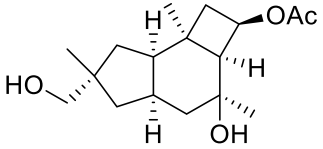	*Conocybe siliginea*, **2015** [12]	No cytotoxicity against human cancer cell lines SK-BR-3, SMMC-7721, HL-60, PANC-1 and A-549 (IC_{50} >40 μM) [12].	**2015**- Relative by ROESY [12].	
Δ^6-Protoilludene **(7)**	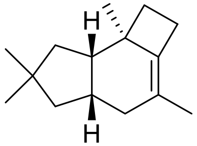	*Fomitopsis insularis*, **1977** [13]		**1985**- Relative established by synthesis [14].	**1985** – racemic [14]
Δ^7-Protoilludene-6-ol **(8)**	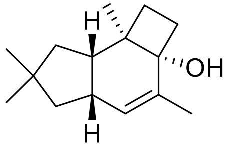	*Fomitopsis insularis*, **1977** [13]			
Δ^7-Protoilludene **(9)**	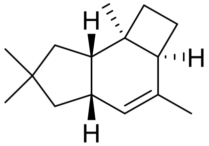	*Dictyostelium discoideum*, **2016** [15]		**2016**- Relative established by NOESY. Absolute established by conversion of synthetic (*R*)-1-^{2}H)FPP to Δ^7-protoilludene [15].	**1979** – racemic [16]
Mucoroidiol (**10**)	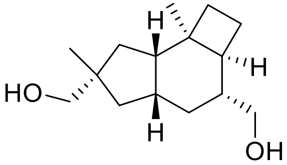	*Dictyostelium mucoroides*, **2020** [17]	No cytotoxicity against HeLa cells (IC_{50} >40 μM). No antibacterial activity against *S. aureus* or *E. coli* [17].	**2020-** Relative established by NOESY. Absolute by biogenetic consideration with Δ^7-protoilludene [17].	

Sulcatine A **(11)**	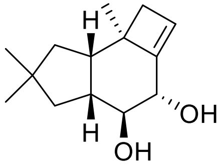	*Laurilia sulcate*, **1987** [18]	No antifungal activity against *C. cucumerinum* (200 µg) and *C. albicans* (200 µg). Antimicrobial activity (MIC 32 µg/mL) against *E. coli* ATCC25922. Inactive against *P. multocida* ATCC15743 and *M. haemolytica* ATCC14003 [9].	**1987**- Relative by NOESY. Absolute established by ECD on dibenzoate derivative [18].	
Sulcatine B **(12)**	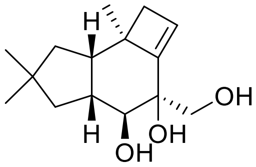	*Laurilia sulcate*, **1992** [19]	Antifungal activity against *C. cucumerinum* (50 µg) and *C. albicans* (50 µg) [19]. Antimicrobial activity (MIC 32 µg/mL) against *E. coli* ATCC25922 and *M. haemolytica* ATCC14003. Inactive against *P. multocida* ATCC15743 [9].	**1992**- Relative by NOESY of sulcatine B and its hydroxy diacetate derivative. Absolute established by biogenetic consideration with sulcatine A [19].	
Armillol **(13)**	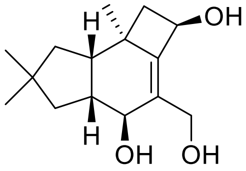	*Laurilia sulcate*, **1992** [19]		**1992**- Relative by NOESY. Absolute established by biogenetic consideration with sulcatine A [19].	
5-*epi*-Armillol **(14)**	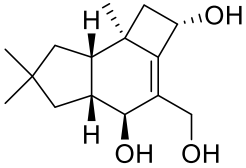	*Laurilia sulcate*, **1992** [19]	Antifungal activity against *C. cucumerinum* (50 µg) and *C. albicans* (50 µg) [19].	**1992**- Relative established by biogenetic consideration with sulcatine A and B	

				and 3-*epi*-illudol [19].	
Tsugicoline A **(15)**	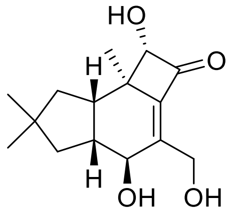	*Laurilia tsugicola*, **1995** [20]	Allelopathic activity against *L. sativum*. Inactive against *B. subtilis*, *B. cereus*, *S. lutea*, *C. cladosporioides* and *S. cerevisiae* at 100 μg [20]. Antimicrobial activity (MIC 32 μg/mL) against *E. coli* ATCC25922 and *M. haemolytica* ATCC14003 but inactive against *P. multocida* ATCC15743 [9].	**1995**- Relative established by NOESY. Absolute established by partial resolution method of Horeau [20].	
Tsugicoline B **(16)**	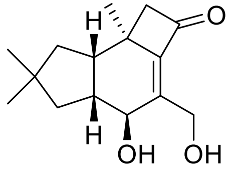	*Laurilia tsugicola*, **1995** [20]		**1995**- Relative established by biogenetic consideration with A [20].	
Tsugicoline C **(17)**	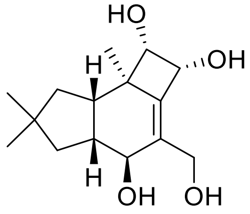	*Laurilia tsugicola*, **1995** [20]	Antimicrobial activity (MIC 32 μg/mL) against *E. coli* ATCC25922 and *M. haemolytica* ATCC14003 but inactive against *P. multocida* ATCC15743 [9].	**1995**- Relative established by NOESY [20].	

Tsugicoline D **(18)**	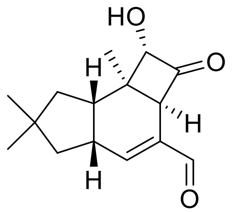	*Laurilia tsugicola*, **1995** [20]		**1995**- Relative established by NOESY [20].	
Tsugicoline E **(19)**	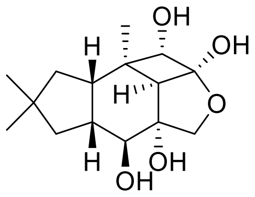	*Laurilia tsugicola*, **1999** [21]	Antimicrobial activity against *E. coli* ATCC25922 MIC 32 µg/mL but inactive against *P. multocida* ATCC15743 and *M. haemolytica* ATCC14003 [9]. Inactive against *B. subtilis*, *B. cereus* and *S. cerevisiae* at 100 µg [21].	**1999**- Absolute established by X-ray [21].	
Tsugicoline M **(20)**	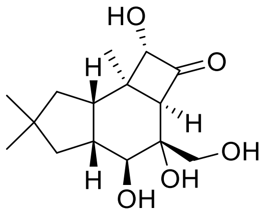	*Clavicorona pyxidata* YB2005, **2009** [22]	No antimicrobial activity against *E. coli*, *B. subtilis*, *S. aureus* and *C. albicans* at 50 µg/mL. No cytotoxicity against HeLa cells at 20 µg/mL [22].	**2009**- Relative by NOESY [22]	
Pyxidatol A **(21)**	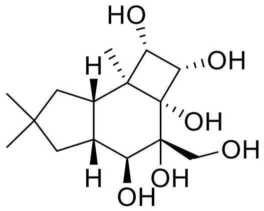	*Clavicorona pyxidata*, **2008** [23]		**2008**- Relative by ROESY. Absolute established by biogenetic consideration with tsugicoline E [23].	

Pyxidatol B (**22**)	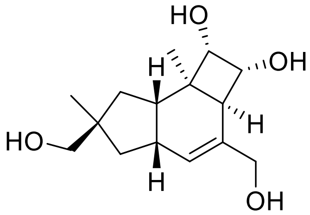	*Clavicorona pyxidata*, **2008** [23]		**2008**- Relative by ROESY. Absolute established by biogenetic consideration with tsugicoline E [23].	
Plorantinone A (**23**)	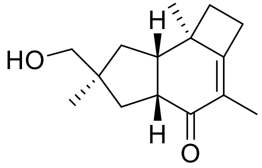	*Russula delica*, **1997** [24]		**1997** – Relative by NOESY. Absolute established by biogenetic consideration to plorantinone B [24].	
Plorantinone B (**24**)	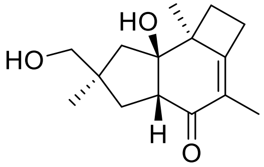	*Russula delica*, **1997** [24]		**1997** – Relative by NOESY. Absolute ECD and conformational analysis and molecular mechanics calculations [24].	
Plorantinone C (**25**)	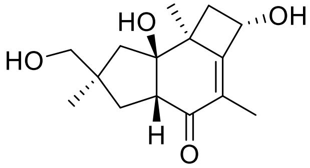	*Russula delica*, **1997** [24]		**1997** – Relative by NOESY. Absolute established by biogenetic consideration with plorantinone B [24].	
Plorantinone D (**26**)	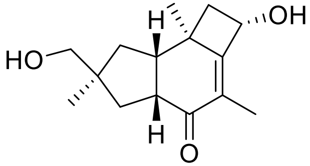	*Russula delica*, **1998** [25]			

epi-Plorantinone B (**27**)	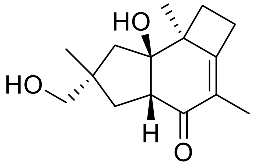	*Russula delica*, **1998** [25]			
Stearoyl plorantinone B (**28**)	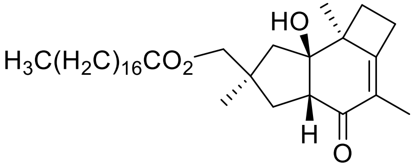	*Russula delica*, **1997** [24]		**1997 –** Relative by NOESY. Absolute established by biogenetic consideration with plorantinone B [24].	
Stearoyl delicone (**29**)	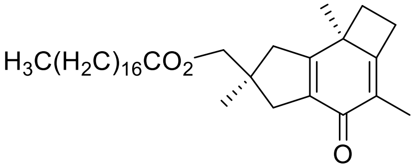	*Russula delica*, **1997** [26]		**1997** - Relative NOESY. Absolute established by biogenetic consideration with plorantinone B [24,26].	
Russujaponol A (**30**)	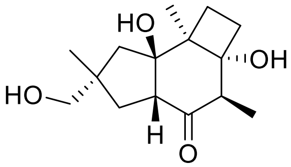	*Russula japonica*, **2006** [27]	Suppressed invasion of human fibrosarcoma (HT1080) cells into Matrigel, 63% inhibition at 3.73 μM.<br>No cytotoxicity against 39 human cancer cell lines [27].	**2006**- Relative by NOESY. Absolute by X-ray of benzoate derivative [27].	
Russujaponol B (**31**)	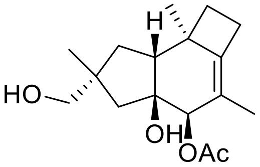	*Russula japonica*, **2006** [27]	No cytotoxicity against 39 human cancer cell lines [27].	**2006**- Relative by NOESY and biogenetic consideration with russujaponol A [27].	

Russujaponol C (**32**)	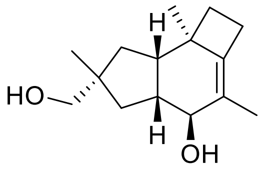	*Russula japonica*, **2006** [27]		**2006**- Relative by NOESY and biogenetic consideration with russujaponol A [27].	
Russujaponol D (**33**)	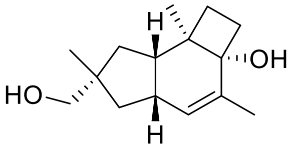	*Russula japonica*, **2006** [27]	No cytotoxicity against 39 human cancer cell lines [27].	**2006**- Relative by NOESY and biogenetic consideration with russujaponol A [27].	
Atlanticone A (**34**)	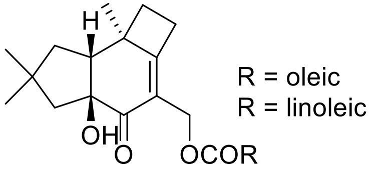	*Lactarius atlanticus*, **2002** [28]	Inactive against *S. aureus* and *E. coli* [28].	**2002** – Relative by NOESY. Absolute by PM3 calculation and ECD comparison with plorantinone B [28].	
Atlanticone B (**35**)	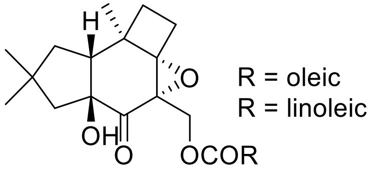	*Lactarius atlanticus*, **2002** [28]	Inactive against *S. aureus* and *E. coli* [28].	**2002**- Relative by NOESY [28].	
Atlanticone C (**36**)	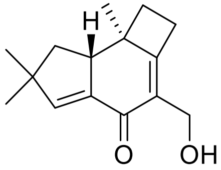	*Lactarius atlanticus*, **2002** [28]		**2002**- Relative by NOESY [28].	**2019 –** Racemic [29] **2020 –** Enantioselective synthesis [30]
Atlanticone D (**37**)	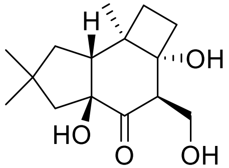	*Lactarius atlanticus*, **2002** [28]		**2002**- Relative by NOESY [28].	

Repraesentin A (**38**)	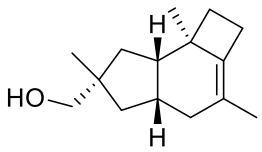	*Lactarius repraesentaneus*, **2003** [31]	Promotion of radicle elongation of lettuce seedlings [31].	**2003**- Relative by NOESY. Absolute by biogenetic consideration with Δ^6-protoilludene [31].	
Pasteurestin A (**39**)	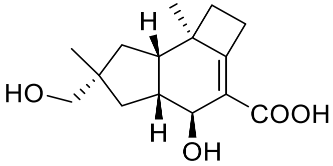	*Agrocybe aegerita*, **2002** [32]	Antimicrobial activity against *P. multocida* (MIC 8 μg/mL) and *M. haemolytica* [9].	**2008**- Absolute established by stereoselective synthesis [33,34].	**2008**- stereoselective synthesis [33,34].
Pasteurestin B (**40**)	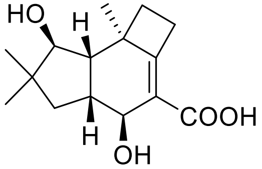	*Agrocybe aegerita*, **2002** [32]	Antimicrobial activity against *P. multocida* (MIC 1 μg/mL) and *M. haemolytica* [9].	**2008**- Absolute established by stereoselective synthesis [33,34].	**2008**- stereoselective synthesis [33,34].
Pasteurestin C (**41**)	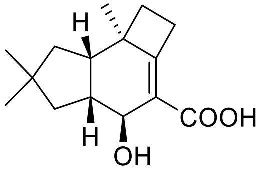	*Agrocybe aegerita*, **2019** [35]		**2019**- Relative by ROESY. Absolute by biogenetic consideration with Pasteurestins A and B [35].	
Epicoterpene A (**42**)	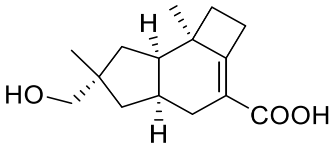	*Armillaria* sp. *and Epicoccum* sp., **2020** [36]	No cytotoxicity against HL-60, A-549, SMMC-7721, MCF-7, SW480 (IC_{50} >40 μM). No acetylcholinesterase inhibitory activity at 50 μM [36].	**2020-** Relative by ROESY. Absolute by ECD [36].	

Epicoterpene B (**43**)	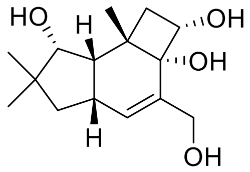	*Armillaria* sp. *and Epicoccum* sp., **2020** [36]	No cytotoxicity against HL-60, A-549, SMMC-7721, MCF-7, SW480 (IC_{50} >40 μM). No acetylcholinesterase inhibitory activity at 50 μM [36].	**2020-** Relative by ROESY. Absolute by ECD [36].	
Epicoterpene C (**44**)	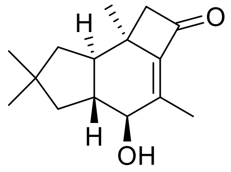	*Armillaria* sp. *and Epicoccum* sp., **2020** [36]	No cytotoxicity against HL-60, A-549, SMMC-7721, MCF-7, SW480 (IC_{50} >40 μM). No acetylcholinesterase inhibitory activity at 50 μM [36].	**2020-** Relative by ROESY. Absolute by ECD [36].	
Epicoterpene E (**45**)	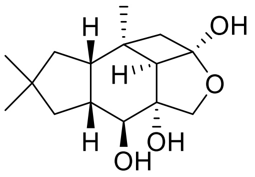	*Armillaria* sp. *and Epicoccum* sp., **2020** [36]	No cytotoxicity against HL-60, A-549, SMMC-7721, MCF-7, SW480 (IC_{50} >40 μM). No acetylcholinesterase inhibitory activity at 50 μM [36].	**2020-** Relative by ROESY. Absolute by ECD [36].	
Lentinellic acid (**46**)	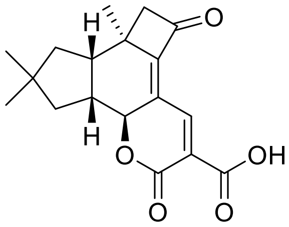	*Lentinellus ursinus* and *L. omphalodes*, **1988** [37]	Antifungal activity against *Absidia glauca* and *Nematospora coryli* (100 μg/disk). Antibacterial activity against *Acinetobacter aerogenes* (MIC 20–50 μg/mL), *B. subtilis* (MIC 20–50 μg/mL), *Micrococcus luteus* (MIC 10–20 μg/mL), *Proteus vulgaris* (MIC 20–50 μg/mL), *S. aureus* (MIC	**1988-** Relative by X-Ray [37].	

			20–50 μg/mL), *Streptomyces* sp. (MIC 10–20 μg/mL), *Aerobacter aerogenes* (MIC 1–5 μg/mL), *B. brevis* (MIC 1–5 μg/mL), and *Corynebacterium insidiosum* (MIC 1–5 μg/mL) [37].		
Lentinellone (**47**)	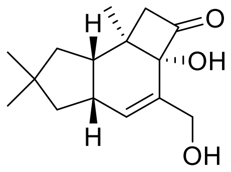	*Lentinellus cochleatus*, **1996** [38]	Moderate cytotoxicity against HL-60 (IC_{50} 20 μg/mL). No cytotoxicity against HeLa, BHK, L1210 (IC_{50} >100 μg/mL). No antimicrobial activity against *A. calcoaceticus, A. citreus, B. brevis, B. subtilis, Corynebacterium insidiosum, E. coli, M. luteus, M. phlei, S. thyphimurium, F. oxysporum, M. miehei, N. fulvescens. N. coryli, P. variotii, R. glutinis, S. cerevisiae* and *U. nuda* [38].	**1996-** Relative by NOESY. Absolute by biogenetic consideration [38].	
Radudiol (**48**)	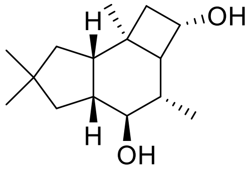	*Radulomyces confluens*, **1988** [39]	Cytotoxicity against HeLa S3 (IC_{50} >400 μM), HL-60 (IC_{50} >400 μM) and L-1210 (IC_{50} 188 μM) [39].	**1988**- Relative by NOESY [39].	**2016**- Stereoselective synthesis of non-natural enantiomer [40].

Radulone A (**49**)	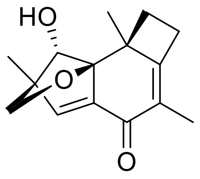	*Radulomyces confluens*, **1988** [39]	Potent inhibitor of human and bovine platelet aggregation [39]. No haemolytic effects on bovine erythrocytes at concentration of 400 μM [39]. Cytotoxicity against HeLa S3 (IC_{50} 16 μM), COS-7 (IC_{50} 20 μM), HL-60 (IC_{50} 2 μM) and L-1210 (IC_{50} 1 μM) [39]. Antimicrobial activity against *C. insiduodum* (MIC 20 μM), *M. luteus* (MIC 20 μM), *S. bikiniensis* (MIC 40 μM), *N. coryli* (MIC 20 μM), *S. cerevisiae* (MIC 41 μM), *M. miehei* (MIC 41 μM) and *U. nuda* (MIC 20 μM) [39].	**1988**- Relative by NOESY [39].	
Radulone B (**50**)	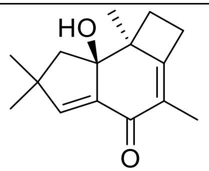	*Radulomyces confluens*, **1988** [39]	Cytotoxicity against HeLa S3 (IC_{50} >400 μM), HL-60 (IC_{50} 431 μM) and L-1210 (IC_{50} 431 μM) [39].	**1988**- Relative by NOESY [39].	
Coprinolone (**51**)	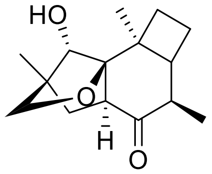	*Coprinus psychromorbidus*, **1988** [41]		**1989**- Relative by NOESY [42].	

Δ^6-Coprinolone (**52**)	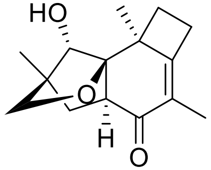	*Coprinus psychromorbidus*, **1989** [42]			
2-Hydroxycoprinolone (**53**)	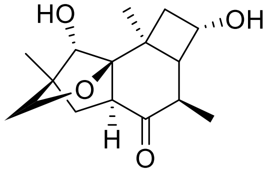	*Granulobasidium vellereum*, **2013** [43]	No antifungal activity against *P. canescens*, *F. oxysporum*, *H. occidentale*, *C. sporulosum*, *C. puteana* and *B. adusta* [43].	**2013**- Absolute by ROESY and ECD [43].	
2a-Hydroxycoprinolone (**54**)	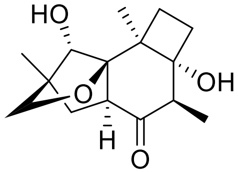	*Granulobasidium vellereum*, **2014** [44]	No antifungal activity against *P. canescens*, *F. oxysporum* and *H. occidentale* at 100 µg/mL [44].	**2014**- Relative by ROESY. Absolute by ECD. [44]	
3-Hydroxycoprinolone (**55**)	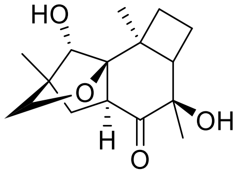	*Granulobasidium vellereum*, **2014** [44]	No antifungal activity against *P. canescens*, *F. oxysporum* and *H. occidentale* at 100 µg/mL [44].	**2014**- Relative by ROESY. Absolute by ECD [44].	
Coprinolone diol B (**56**)	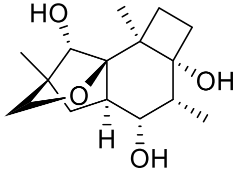	*Granulobasidium vellereum*, **2014** [44]	No antifungal activity against *P. canescens*, *F. oxysporum* and *H. occidentale* at 100 µg/mL [44].	**2014**- Relative by ROESY. Absolute by biogenetic consideration with coprinolone diol [44].	
8-Deoxy-4a-hydroxytsugicoline A (**57**)	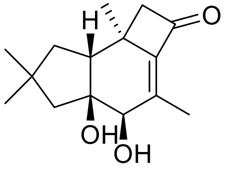	*Granulobasidium vellereum*, **2013** [43]	No antifungal activity against *P. canescens*, *F. oxysporum*, *H. occidentale*, *C. sporulosum*, *C. puteana* and *B. adusta* [43].	**2013**- Relative by ROESY. Absolute by biogenetic consideration with 2-hydroxycoprinolone [43].	

8-Deoxy-4a-hydroxytsugicoline B (**58**)	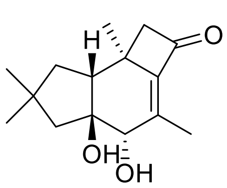	*Granulobasidium vellereum*, **2014** [44]	No antifungal activity against *P. canescens*, *F. oxysporum* and *H. occidentale* at 100 µg/mL [44].	**2014**- Relative by ROESY. Absolute by biogenetic consideration with 8-deoxy-4a-hydroxytsugicoline [44].	
8-Deoxydihydrotsugicoline (**59**)	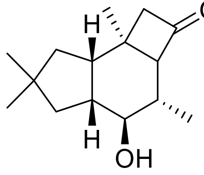	*Granulobasidium vellereum*, **2013** [43]	No antifungal activity against *P. canescens*, *F. oxysporum*, *H. occidentale*, *C. sporulosum*, *C. puteana* and *B. adusta* [43].	**2013**- Relative by ROESY. Absolute by biogenetic consideration with 2-hydroxycoprinolone and ECD [43].	**2016**- Stereoselective synthesis of non-natural enantiomer [40].
Granulodiene A (**60**)	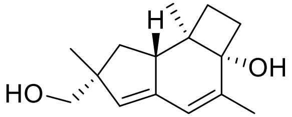	*Granulobasidium vellereum*, **2014** [44]	No antifungal activity against *P. canescens*, *F. oxysporum* and *H. occidentale* at 100 µg/mL [44].	**2014**- Relative by ROESY. Absolute by ECD [44].	
Granulodiene B (**61**)	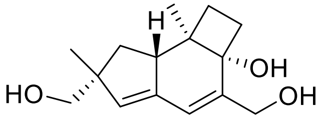	*Granulobasidium vellereum*, **2014** [44]	No antifungal activity against *P. canescens*, *F. oxysporum* and *H. occidentale* at 100 µg/mL [44].	**2014**- Relative by ROESY. Absolute by ECD [44].	
Granulone A (**62**)	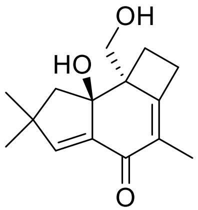	*Granulobasidium vellereum*, **2014** [44]	No antifungal activity against *P. canescens*, *F. oxysporum* and *H. occidentale* at 100 µg/mL [44].	**2014**- Relative by ROESY. Absolute by biogenetic consideration with radulone B [44].	

Granulone B (**63**)	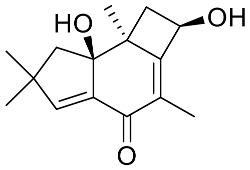	*Granulobasidium vellereum*, **2014** [44]	No antifungal activity against *P. canescens*, *F. oxysporum* and *H. occidentale* at 100 µg/mL [44].	**2014**- Relative by ROESY. Absolute by biogenetic consideration with granulone A [44].	
Demethylgranulone (**64**)	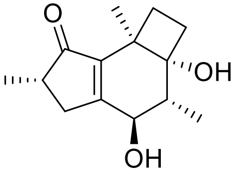	*Granulobasidium vellereum*, **2014** [44]	No antifungal activity against *P. canescens*, *F. oxysporum* and *H. occidentale* at 100 µg/mL [44].	**2014**- Relative by ROESY. Absolute by biogenetic consideration with protoilludanes [44].	
Echinocidin A (**65**)	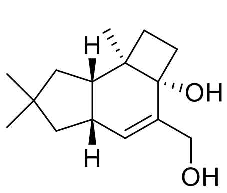	*Echinodontium tsugicola*, **2004** [45]	Inactive against *C. albicans* ATCC2019, *S. aureus* NBRC13276 and *P. aeruginosa* ATCC15442 [45].	**2004**- Relative by NOESY. Absolute established by biogenetic consideration with tsugicoline A and B [45].	
Echinocidin B (**66**)	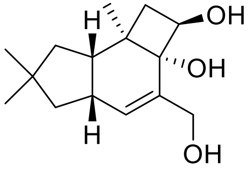	*Echinodontium tsugicola*, **2004** [45]	Inactive against *C. albicans* ATCC2019, *S. aureus* NBRC13276 and *P. aeruginosa* ATCC15442 [45].	**2004**- Relative by NOESY. Absolute established by biogenetic consideration with tsugicoline A and B [45].	**2019 –** Racemic [46]
Echinocidin C (**67**)	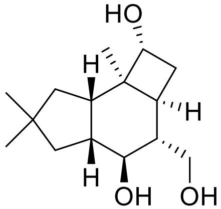	*Echinodontium tsugicola*, **2005** [47]		**2005**- Relative by NOESY. Absolute established by biogenetic consideration with tsugicoline A [47].	

Echinocidin D (**68**)	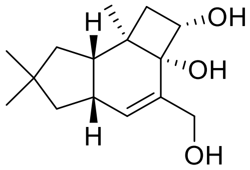	*Echinodontium tsugicola*, **2005** [47]		**2005**- Relative by NOESY. Absolute established by biogenetic consideration with tsugicoline A [47].	**2019** – Racemic [46]
Armillyl orsellinate (**69**)	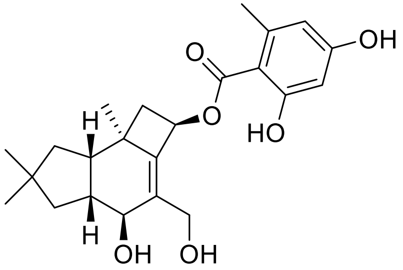	*Armillaria mellea*, **1982** [48]	Antibacterial activity against *B. subtilis* ATCC6633 (5.6γ) and *S. aureus* ATCC53156 (5.6γ) [48]. Antibacterial activity against *B. subtilis* (0.5 µg) and *E. coli* (1.0 µg) [49]. Antifungal activity against *C. cucumerinum* (inactive) and *C. albicans* (1.0 µg) [49].	**1982**- Absolute by ECD and X-ray of hydroxyaldehyde derivative [48,50].	
Judeol (**70**)	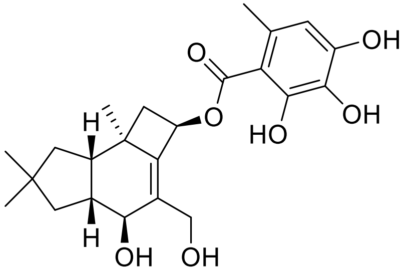	*Armillaria mellea*, **1985** [51]	Antibacterial activity against *B subtilis* ATCC6633 (5.6γ) and *S. aureus* ATCC53156 (8.7γ) [51]. No activity against *P. aeruginosa* or *E. coli* [51].		
Armillyl everninate (**71**)	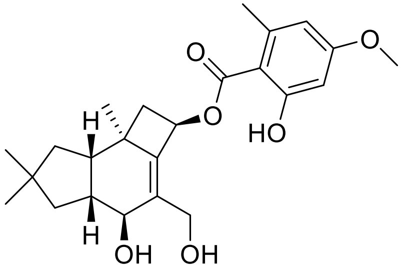	*Armillaria mellea*, **1986** [52]	No antibacterial activity (Gram-positive) [52].		

Arnamiol (**72**)	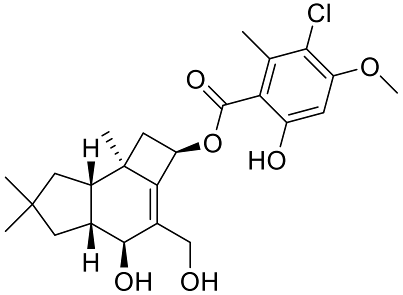	*Armillaria mellea*, **1986** [52]	No antibacterial activity (Gram-positive) [52].		
Armillaribin (**73**)	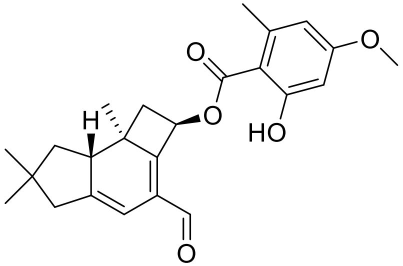	*Armillaria mellea*, **1988** [53]			
Armillaricin (**74**)	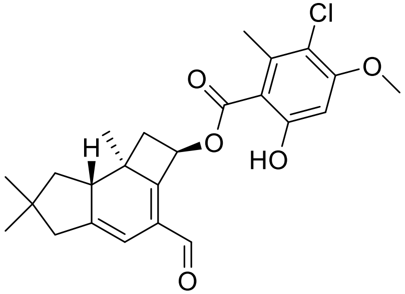	*Armillaria mellea*, **1989** [54]	Cytotoxicity against MCF7 (IC_{50} 4.8±0.4 μM), H460 (IC_{50} 5.5±0.4 μM), HT-29 (IC_{50} 4.6±0.3 μM) and CEM (IC_{50} 5.8±0.6 μM) [55].	**1989**- X-ray [54].	
Arnamial (**75**)	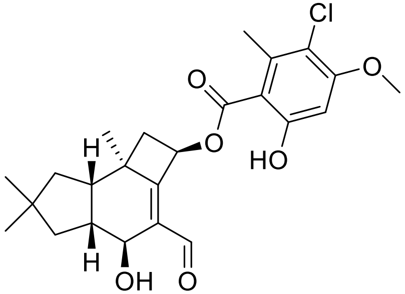	*Armillaria mellea*, **2009** [56]	Cytotoxicity against HCT-116 (IC_{50} 10.69 μM), MCF7 (IC_{50} 15.4±0.3μM), Jurkat (IC_{50} 3.93±0.4 μM) and CCRF-CEM (IC_{50} 8.91 μM) [56].<br>Cytotoxicity against HUVEC (GI_{50} 2.0±0.1μM),	**2009**- Relative by biogenetic consideration with armillylorsellinate [56].	

			K-562 (GI_{50} 2.3±0.02 μM) and HeLa (CC_{50} 4.9±0.2 μM) [57]. Bioactivity against *T. panuoides* (MIC <5 μg/mL), *P. ostreatus* (MIC <5 μg/mL), *O. illudens* (MIC <5 μg/mL), *F. pinicola* (MIC <5 μg/mL), *P. oxalicum* (MIC <5 μg/mL), *Aspergillus flavus* (MIC 10 μg/mL), *T. harzianum* (MIC >100 μg/mL), *M. racemosus* (MIC >100 μg/mL) and *S. scabies* (MIC <5 μg/mL) [58]. Inhibition of growth of *P. notatum*, *A. flavus* and *A. nidulans* (MIC 14.0 μM) [57].		
Dehydroarmillylorsellinate (**76**)	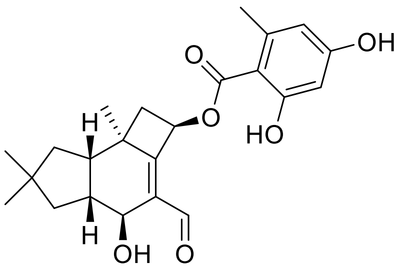	*Armillaria mellea*, **2011** [59]	Cytotoxicity against MCF7 (IC_{50} 8.0±0.5 μM), Jurkat (IC_{50} 16.9±0.1 μM), HeLa (IC_{50} 15.2±2.0 μM) and K-562 (IC_{50} 5.0±0.3 μM) [59]. Cytotoxicity against HUVEC (GI_{50} 5.3±0.5 μM), K-562 (GI_{50} 5.0±0.3 μM), MCF-7 (GI_{50} 8.0±0.5 μM) and HeLa (CC_{50} 15.2±2.0 μM) [57]. Inhibition of growth of *P. notatum*, *A. flavus* and *A.*		

			nidulans (MIC 31.3 μM) [57].		
6′-Dechloroarnamial (**77**)	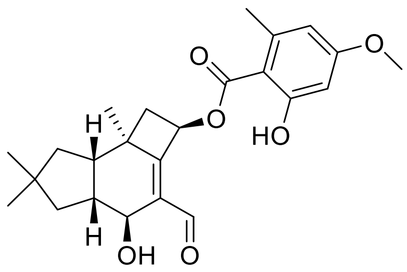	*Armillaria mellea*, **2014** [57]	Inhibited growth of *P. notatum*, *A. flavus* and *A. nidulans* (MIC 15.1 μM) [57]. Cytotoxicity against HUVEC (GI_{50} 5.1±0.1 μM), K-562 (GI_{50} 4.1±0.1 μM), MCF-7 (GI_{50} 4.1±0.3 μM) and HeLa (CC_{50} 12.3±0.3 μM) [57].	**2014**- Relative established by biogenetic consideration with arnamial [57].	
Melleolide A (**78**)	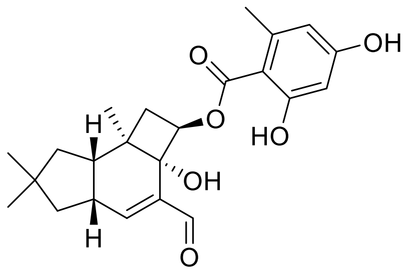	*Armillaria mellea*, **1982** [60]	Antibacterial activity against *B. subtilis* (0.5 μg) and *E. coli* (1.0 μg) [49]. Antifungal activity against *C. cucumerinum* (1.0 μg) and *C. albicans* (1.0 μg) [49]. Cytotoxicity against HepG2 (IC_{50} 4.95±1.79 μM) and L02 (IC_{50} 16.05±2.89 μM) [61]. Inhibited mycelial growth of *C. cinerea* and *F. velutipes*. Antimicrobial activity at 250 μg/mL against *S. aureus* (9.1mm), *M. luteus* (10.0mm) and *C. albicans* (14.5 mm) .	**1982**- Absolute by X-ray [60].	**2019 –** Racemic [46]

Melleolide B (**79**)	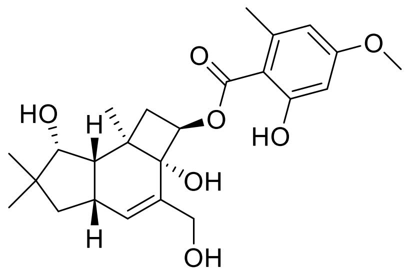	*Armillaria mellea*, **1986** [62]	Antibacterial activity against *B. cereus* (ATCC10702), *B. subtilis* (ATCC6633) and *E. coli* (ATCC10536) [62]. Cytotoxicity against HL-60 (IC_{50} >40 μM), SMMC-7721 (IC_{50} >40 μM), A-549 (IC_{50} >40 μM), MCF-7 (IC_{50} >40 μM) and SW480 (IC_{50} >40 μM) [63].	**1986**- Relative by NOESY [62].	
Melleolide C (**80**)	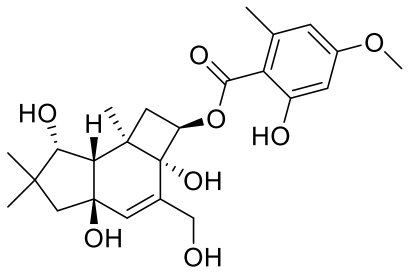	*Armillaria mellea*, **1986** [62]	Antibacterial activity against *B. cereus* (ATCC10702), *B. subtilis* (ATCC6633) and *E. coli* (ATCC10536) [62]. Not cytotoxic against HCT-116, MCF7, Jurkat and CCRF-CEM at IC_{50} 100 μM [56].	**1986**- Relative by NOESY [62].	
Melleolide D (**81**)	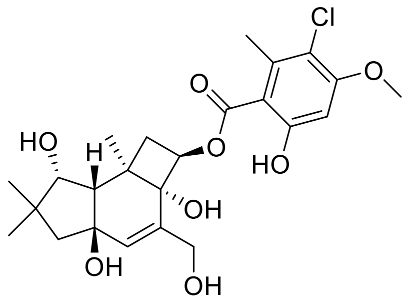	*Armillaria mellea*, **1986** [62]	Antibacterial activity against *B. cereus* (ATCC10702), *B. subtilis* (ATCC6633) and *E. coli* (ATCC10536) [62]. Cytotoxicity against HCT-116 (IC_{50} >100 μM), MCF7 (IC_{50} >100 μM), Jurkat (IC_{50} >100 μM), HeLa (IC_{50} >100 μM), K-562 (IC_{50} >100 μM) and CCRF-CEM (IC_{50} 61.66 μM) [56].	**1986**- Relative by NOESY and biogenetic consideration with melleolide C [62].	

Melleolide E (**82**)	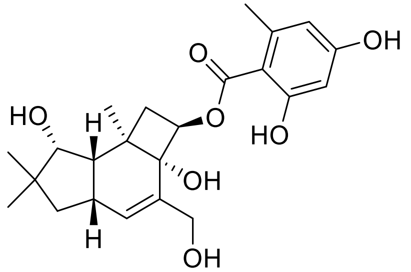	*Armillaria mellea*, **1988** [64]		**1988**- Relative by NOESY [64].	
Melleolide F (**83**)	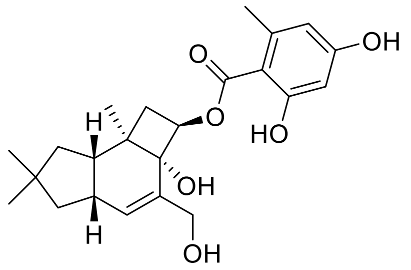	*Armillaria mellea*, **1988** [64]	Cytotoxicity against HL-60 (IC_{50} 20.27 μM), SMMC-7721 (IC_{50} 30.36 μM), A-549 (IC_{50} 16.62 μM), MCF-7 (IC_{50} 16.56 μM) and SW480 (IC_{50} >40 μM) [63]. Cytotoxicity against MCF7 (IC_{50} 8.3±2.2 μM), H460 (IC_{50} 5.1±0.2 μM), HT-29 (IC_{50} 58.4±4.9 μM) and CEM (IC_{50} 41.2±3.4 μM) [55].	**1988**- Relative by NOESY [64].	**2019 –** Racemic [46]
Melleolide G (**84**)	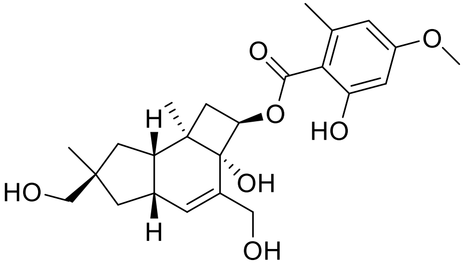	*Armillaria mellea*, **1988** [64]		**1988**- Relative by NOESY [64].	

Melleolide H (**85**)	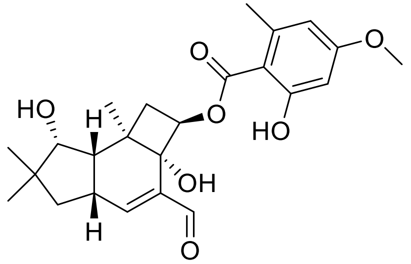	*Armillaria mellea*, **1988** [64]		**1988**- Relative by NOESY [64].	
Melleolide I / 5′-Chloromelleolide (**86**)	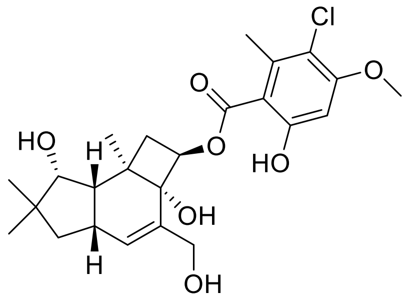	*Armillaria novae-zelandiae*, **1988** [65]	Cytotoxicity against HL-60 (IC_{50} >40 μM), SMMC-7721 (IC_{50} >40 μM), A-549 (IC_{50} 20.11 μM), MCF-7 (IC_{50} 30.06 μM) and SW480 (IC_{50} >40 μM).	**1998**- Relative by NOESY [65].	
Melleolide J / Armillarikin (**87**)	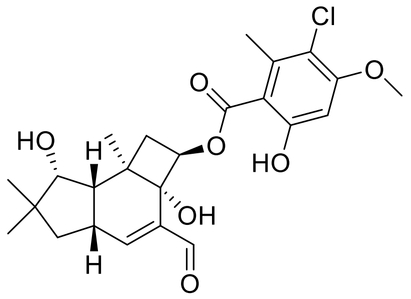	*Armillaria novae-zelandiae*, **1988** [65]	Cytotoxicity against HL-60 (IC_{50} 14.11 μM), SMMC-7721 (IC_{50} 17.41 μM), A-549 (IC_{50} 14.73 μM), MCF-7 (IC_{50} 11.52 μM) and SW480 (IC_{50} 19.89 μM) [63]. Cytotoxicity against MCF7 (IC_{50} 4.4±0.8 μM), H460 (IC_{50} 5.7±0.5 μM), HT-29 (IC_{50} 34.7±4.6 μM) and CEM (IC_{50} 44.6±4.4 μM) [55]. Inhibited mycelial growth of *C. cinerea*.	**1998**- Relative by NOESY [65].	

Melleolide K (**88**)	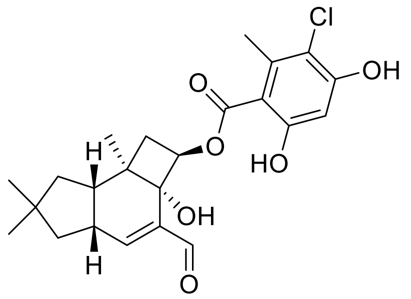	*Armillaria mellea*, **2000** [66]	Antimicrobial activity against *S. aureus* FDA209P (MIC 6.25 μg/mL), *S. aureus* Smith (MIC 12.5 μg/mL), *S. aureus* MS9610 (MIC 12.5 μg/mL), *S. aureus* MC16526 (MRSA) (MIC 12.5 μg/mL), *S. aureus* TY-04282 (MRSA) (MIC 12.5 μg/mL), *M. luteus* IFO3333 (MIC 12.5 μg/mL), *B. subtilis* PCI219 (MIC 6.25 μg/mL), *C. bovis* 1810 (MIC 25 μg/mL), *M. smegmatis* ATCC607 (MIC 50 μg/mL), *C. pseudotropicalis* (MIC 50 μg/mL), *C. neoformans* (MIC 50 μg/mL), *S. cerevisiae* (MIC 25 μg/mL), *T. rubrum* IFO9185 (MIC 25 μg/mL), *T. mentagrophytes* 833 (MIC 50 μg/mL) and *A. fumigatus* TIMM 2905 (MIC 50 μg/mL) [66].	**2000**- Relative by NOESY [66].	

Melleolide L (**89**)	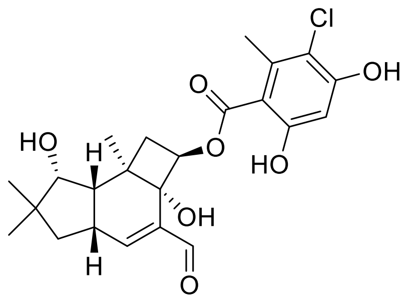	*Armillaria mellea*, **2000** [66]	Antimicrobial activity against *S. aureus* FDA209P (MIC 50 μg/mL), *S. aureus* Smith (MIC 100 μg/mL), *S. aureus* MS9610 (MIC 100 μg/mL), *S. aureus* MC16526 (MRSA) (MIC 100 μg/mL), *M. luteus* IFO3333 (MIC 50 μg/mL), *B. subtilis* PCI219 (MIC 50 μg/mL), *M. smegmatis* ATCC607 (MIC 50 μg/mL), *C. pseudotropicalis* (MIC 100 μg/mL) and *S. cerevisiae* (MIC 100 μg/mL) [66].	**2000**- Absolute established by X-ray of 2,4-dinitrophenylhydrazone derivative [66].	
Melleolide M (**90**)	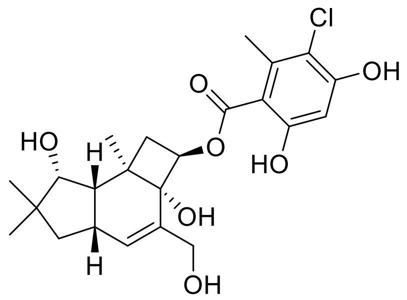	*Armillaria mellea*, **2000** [66]	Antimicrobial activity against *S. aureus* FDA209P (MIC 50 μg/mL), *S. aureus* Smith (MIC 100 μg/mL), *S. aureus* MS9610 (MIC 100 μg/mL), *S. aureus* MC16526 (MRSA) (MIC 100 μg/mL), *S. aureus* TY-04282 (MRSA) (MIC 100 μg/mL), *K. pneumoniae* PCI602 (MIC 50 μg/mL), *C. pseudotropicalis* (MIC 100 μg/mL) and *T. rubrum* IFO9185 (MIC 50 μg/mL) [66].	**2000**- Absolute established by conversion of melleolide L into M [66].	

Melleolide N (**91**)	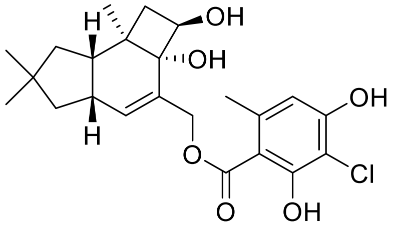	*Armillaria mellea*, **2015** [55]	Cytotoxicity against MCF7 (IC_{50} 56.5±4.2 μM), H460 (IC_{50} 5.5±0.6 μM), HT-29 (IC_{50} 7.1±0.8 μM) and CEM (IC_{50} 5.4±0.3 μM) [55].	**2015**- Relative by NOESY [55].	
6′-Chloromelleolide F/ Melleolide P (**92**)	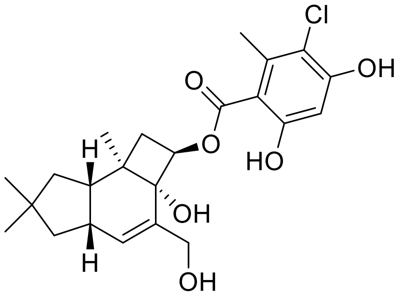	*Armillaria mellea*, **2011** [67]	Cytotoxicity against HUVEC (GI_{50} 62.2±1.7 μM), K-562 (GI_{50} 39.0±2.1 μM), MCF-7 (GI_{50} 46.3±0.5 μM) and HeLa (CC_{50} 50.9±1.1 μM) [57]. Cytotoxicity against MCF7 (IC_{50} 4.8±0.5 μM), H460 (IC_{50} 4.5±0.4 μM), HT-29 (IC_{50} 56.7±4.5 μM) and CEM (IC_{50} 28.8±1.2 μM) [55].	**2014**- Relative established by biogenetic consideration with melleolide F [57].	
13-Hydroxymelleolide K/ Melleolide T (**93**)	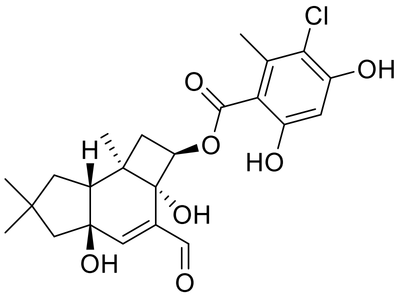	*Armillaria mellea*, **2011** [67]	Cytotoxicity against MCF7 (IC_{50} >100 μM), H460 (IC_{50} >100 μM), HT-29 (IC_{50} 32.1±3.6 μM) and CEM (IC_{50} 5.5±0.6 μM) [55].	**2016-** Relative by biogenetic consideration with melleolide D. Absolute established by ECD [61].	

4-*O*-Methylmelleolide (**94**)	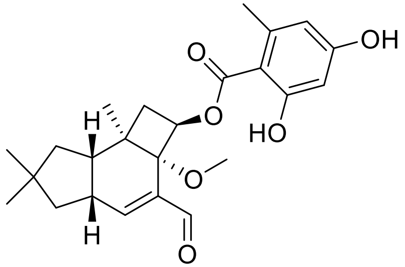	*Armillaria mellea*, **1985** [51]	Antibacterial activity against *B. subtilis* ATCC6633 (5.6γ) and *S. aureus* ATCC53156 (8.7γ). No activity against *P. aeruginosa* or *E. coli* [51].	**1985**- Absolute by X-ray [51].	
Dihydromelleolide (**95**)	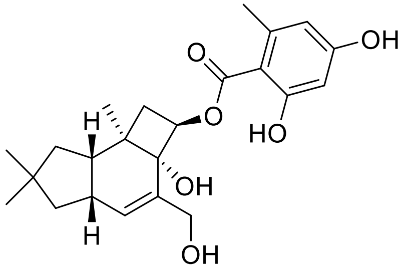	*Armillaria mellea*, **1988** [68]			
13-Hydroxydihydromelleolide (**96**)	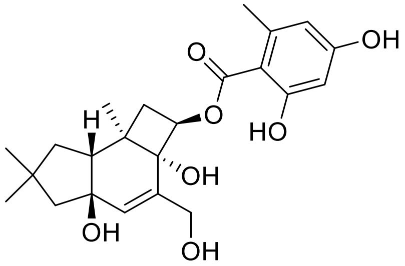	*Armillaria mellea*, **1990** [69]	Cytotoxicity against MCF7 (IC_{50} >100 μM), Jurkat (IC_{50} >100 μM), HeLa (IC_{50} >100 μM) and K-562 (IC_{50} >100 μM).	**1990**- Relative by NOESY [69].	
10α-Hydroxymelleolide (**97**)	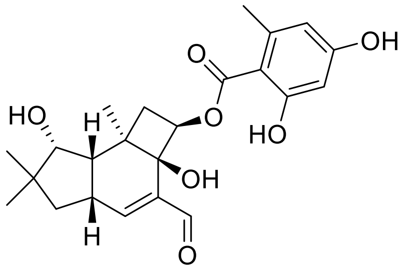	*Armillaria mellea*, **1990** [69]	Cytotoxicity against HCT-116 (IC_{50} >100 μM), MCF7 (IC_{50} >100 μM), Jurkat (IC_{50} 86.17 μM) and CCRF-CEM (IC_{50} 63.23 μM) [56]. Antibacterial activity against *B. subtilis* (0.5 μg) and *E. coli* (1.0 μg) [49]. Antifungal activity against *C. cucumerinum* (1.0 μg)		

			and *C. albicans* (1.0 µg) [49].<br>Bioactivity against *T. panuoides* (MIC 100 µg/mL), *P. ostreatus* (MIC >100 µg/mL), *O. illudens* (MIC 50 µg/mL), *F. pinicola* (MIC >100 µg/mL), *P. oxalicum* (MIC >100 µg/mL), *A. flavus* (MIC >100 µg/mL), *T. harzianum* (MIC >100 µg/mL), *M. racemosus* (MIC >100 µg/mL) and *S. scabies* (MIC >100 µg/mL) [58].		
10*α*-Hydroxydihydromelleolide (**98**)	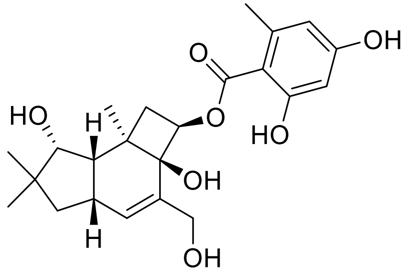	*Armillaria mellea*, **1990** [69]			
13-Hydroxy-4-methoxymelleolide (**99**)	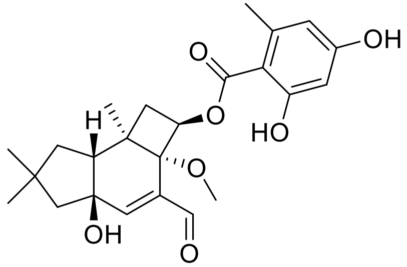	*Armillaria mellea*, **1995** [70]		**1997**- Relative by NOESY [69].	

4-Dehydromelleolide (**100**)	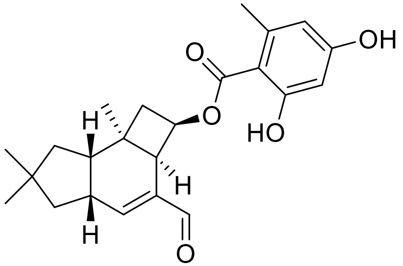	*Armillaria mellea*, **1995** [68]			
6′-Chloro-10α-hydroxymelleolide (**101**)	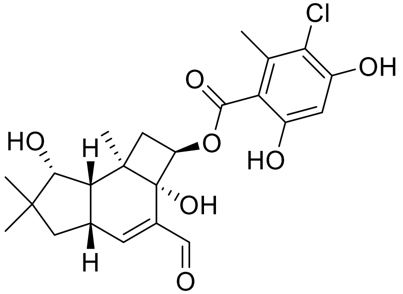	*Armillaria mellea*, **2000** [49]	Antibacterial activity against *B. subtilis* (0.5 μg) and *E. coli* (1.0 μg) [49]. Antifungal activity against *C. cucumerinum* (1.0 μg) and *C. albicans* (1.0 μg) [49].	**2000**- Relative by NOESY [49].	
6′-Chloro-13-hydroxy-dihydromelleolide (**102**)	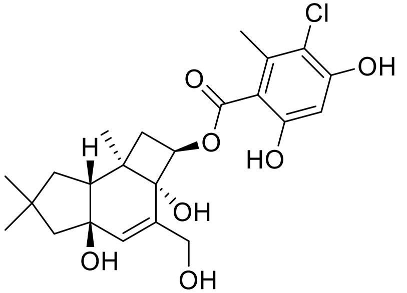	*Armillaria mellea*, **2011** [59]	Cytotoxicity against MCF7 (IC_{50} >100 μM), Jurkat (IC_{50} 46.6±3.1 μM), HeLa (IC_{50} >100 μM) and K-562 (IC_{50} >100 μM) [59].		
Melledonal A (**103**)	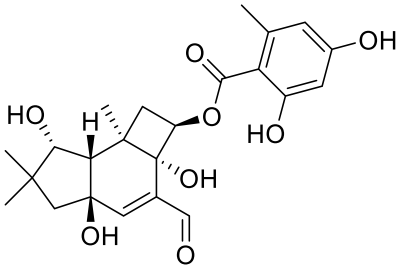	*Armillaria mellea*, **1985** [70]	Not cytotoxic against HCT-116, MCF7, Jurkat and CCRF-CEM at IC_{50} 100 μM [56]. Bioactivity against *T. panuoides* (MIC 50 μg/mL), *P. ostreatus* (MIC 100 μg/mL), *O. illudens* (MIC	**1985**- Relative established by NOESY of diacetate derivative [70].	

			50 μg/mL), *F. pinicola* (MIC 50 μg/mL), *P. oxalicum* (MIC >100 μg/mL), *A. flavus* (MIC >100 μg/mL), *T. harzianum* (MIC >100 μg/mL), *M. racemosus* (MIC >100 μg/mL) and *S. scabies* (MIC >100 μg/mL) [58].		
Melledonal B (**104**)	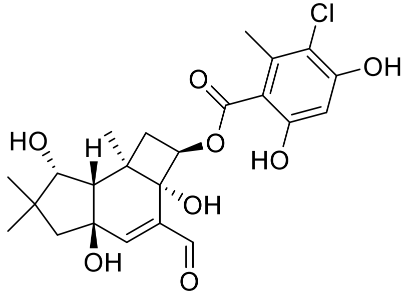	*Armillaria mellea*, **1988** [71]	Cytotoxicity (IC_{50} >100 μM) against MCF7, H460, HT-29 and CEM [55]. Antibacterial activity (100 μg) against *B. cereus* (ATCC10702) and *B. subtilis* (ATCC6633) [71].	**1988**- Absolute by biogenetic consideration with C [71].	
Melledonal C (**105**)	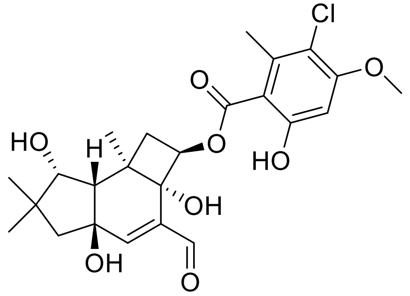	*Armillaria mellea*, **1988** [71]	Cytotoxicity against HCT-116 (IC_{50} >100 μM), MCF7 (IC_{50} >100 μM), Jurkat (IC_{50} 58.75 μM) and CCRF-CEM (IC_{50} 14.75 μM) [56]. Cytotoxicity against MCF7 (IC_{50} >100 μM), H460 (IC_{50} >100 μM), HT-29 (IC_{50} 85.6±9.1 μM) and CEM (IC_{50} 49.6±5.2 μM) [55]. Antibacterial activity against *B. subtilis* (0.5 μg) and *E. coli* (1.0 μg) [49]. Antifungal activity against *C. cucumerinum* (inactive)	**1988**- Absolute by X-ray [71].	

			and *Candida albicans* (3.0 μg) [49]. Antimicrobial activity (MIC 32 μg/mL) against *E. coli* ATCC25922 [71]. Inactive against *P. multocida* ATCC15743 and *M. haemolytica* ATCC14003 [9].		
15-Hydroxy-5′-*O*-methylmelledonal (**106**)	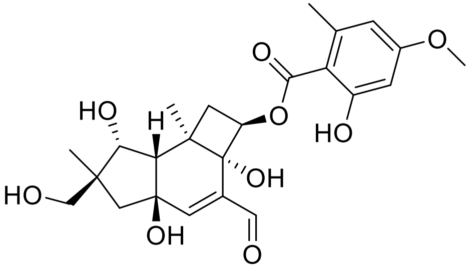	*Armillaria mellea*, **1987** [72]		**1987**- Relative by NOESY on dibenzoate derivative [72].	
5′-*O*-methylmelledonal (**107**)	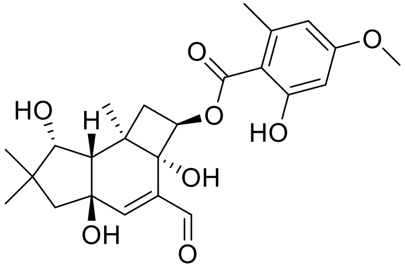	*Armillaria mellea*, **1987** [72]		**1987**- Relative by biogenetic consideration with melleodonal [72].	
Melledonal D (**108**)	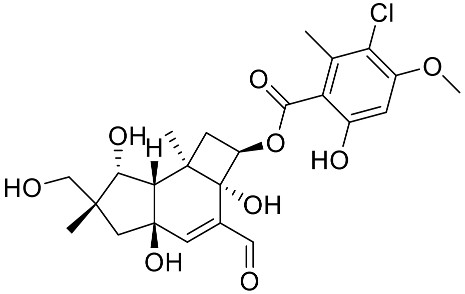	*Clitocybe elegans*, **1988** [64]		**1988**- Relative by NOESY [64].	

Melledonal E (**109**)	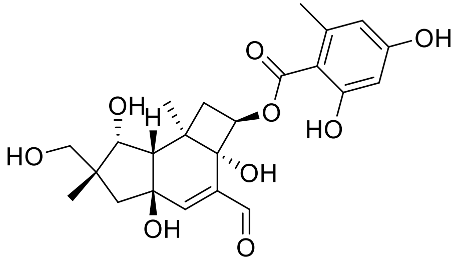	*Clitocybe elegans*, **1988** [64]		**1988**- Relative by NOESY [64].	
Melledonol (**110**)	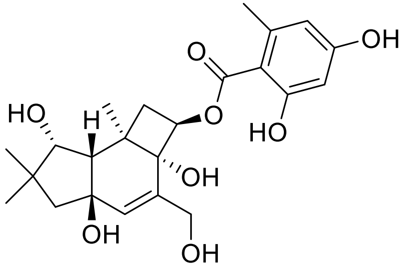	*Armillaria mellea*, **1985** [70]	Not cytotoxic against HCT-116, MCF7, Jurkat and CCRF-CEM at IC_{50} 100 μM [56].		
Armillarin (**111**)	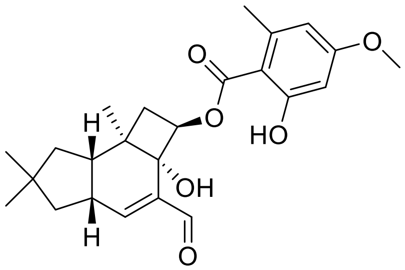	*Armillaria mellea*, **1984** [73]	No antibiotic activity against *H. annosum*, *G. abietinum*, *E. coli*, *M. luteus*, *B. subtilis*, *C. albicans* and *S. aureus* [74]. Cytotoxicity against MCF7 (IC_{50} 11.6±0.5 μM), HeLa (IC_{50} 16.7±2.1 μM), K-562 (IC_{50} 9.9±0.6 μM) [59]. Cytotoxicity against HUVEC (GI_{50} 9.9±0.8 μM), K-562 (GI_{50} 9.9±0.6 μM), MCF-7 (GI_{50} 11.6±0.5 μM) and HeLa (CC_{50} 16.7±2.1 μM) [57].	**1984**- Absolute by X-ray [73].	

Armillaridin (**112**)	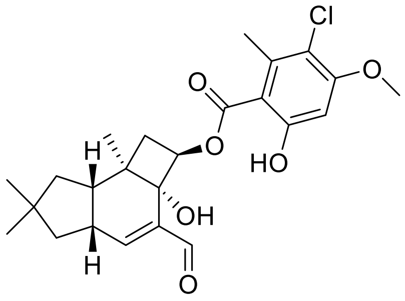	*Armillaria mellea*, **1984** [73]	Cytotoxicity against MCF7 (IC_{50} 7.8±0.9 μM), Jurkat (IC_{50} 3.0±0.3 μM), HeLa (IC_{50} 9.2±1.6 μM), K-562 (IC_{50} 8.9±1.3 μM), HepG2 (IC_{50} 13.37±2.69 μM) and L02 (IC_{50} 12.15±0.95 μM) [59,61]. Cytotoxicity against HUVEC (GI_{50} 7.8±0.3 μM), K-562 (GI_{50} 8.9±1.3 μM), MCF-7 (GI_{50} 7.8±0.9 μM) and HeLa (CC_{50} 9.2±1.6 μM) [57]. Cytotoxicity against MCF7 (IC_{50} 1.7±0.2 μM), H460 (IC_{50} 4.5±0.3 μM), HT-29 (IC_{50} 42.1±5.1 μM) and CEM (IC_{50} 5.1±0.4 μM) [55].	**1984**- Relative by NOESY. Absolute by biogenetic consideration with armillarin [73].	
Armillarigin (**113**)	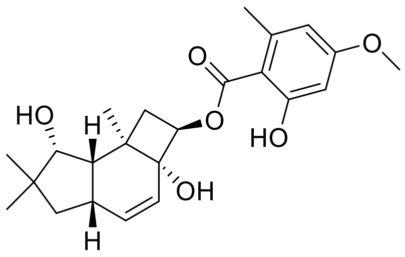	*Armillaria mellea*, **1989** [75]	Cytotoxicity against HL-60 (IC_{50} 14.27 μM), SMMC-7721 (IC_{50} 19.51 μM), A-549 (IC_{50} 10.01 μM), MCF-7 (IC_{50} 10.67 μM), SW480 (IC_{50} 19.19 μM), HepG2 (IC_{50} 37.65±3.46 μM) and L02 (IC_{50} 69.10±5.86 μM) [61,63].		

Armillarilin (**114**)	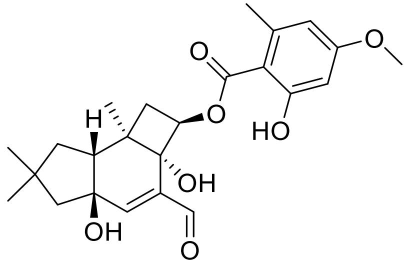	*Armillaria mellea*, **1990** [76]	Cytotoxicity against HepG2 (IC_{50} 13.25±0.95 μM) and L02 (IC_{50} 18.00±3.80 μM) [61].		
Armillarinin (**115**)	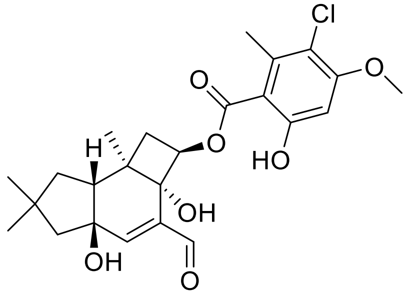	*Armillaria mellea*, **1990** [76]			
Armillaripin (**116**)	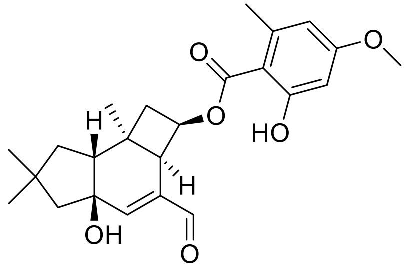	*Armillaria mellea*, **1990** [77]			
Armillaritin (**117**)	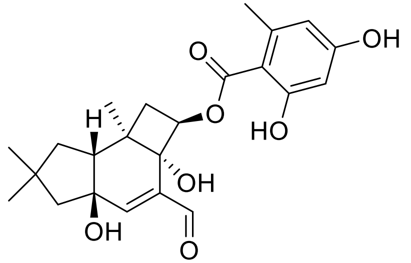	*Armillaria mellea*, **1991** [78]	Cytotoxicity against HepG2 (IC_{50} 12.26±3.02 μM) and L02 (IC_{50} 31.95±1.05 μM) [61].		

Armillarivin (**118**)	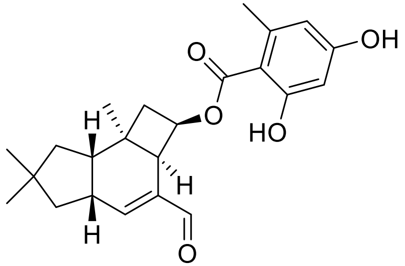	*Armillaria mellea*, **1991** [78]			
Armillasin (**119**)	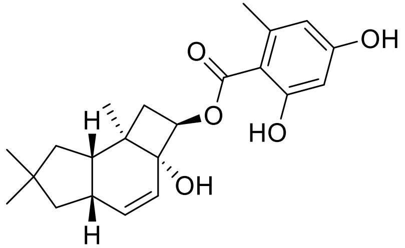	*Armillaria mellea*, **1991** [79]	Cytotoxicity against HepG2 (IC_{50} 15.63±3.35 μM) and L02 (IC_{50} 14.38±3.60 μM) [61].	**1991**- Relative by NOESY [79].	
Armillatin (**120**)	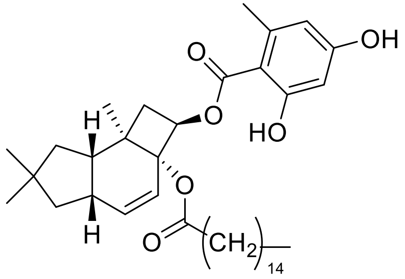	*Armillaria mellea*, **1991** [79]		**1991**- Relative by NOESY [79].	
Armillaric acid (**121**)	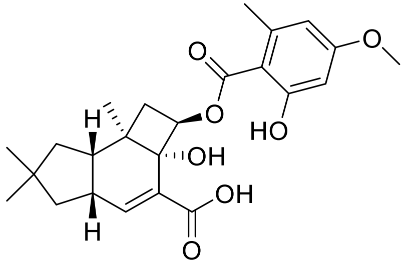	*Armillaria mellea*, **1989** [80]	Antimicrobial activity against at 250 μg/mL *S. aureus* (19.8mm), *M. lutaus* (8.8 mm) and *C. albicans* (10.1 mm) [81].	**1990**- Relative by NOESY [81].	

10α-Hydroxyarmillarin (**122**)	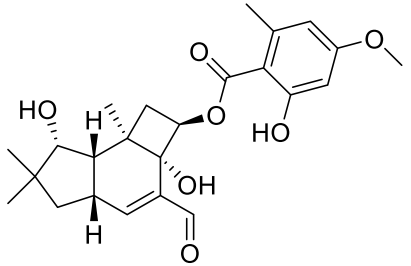	*Armillaria mellea*, **1990** [82]		**1990**- Relative by biogenetic consideration with melleolide B [82].	
4-*O*-Methylarmillaridin (**123**)	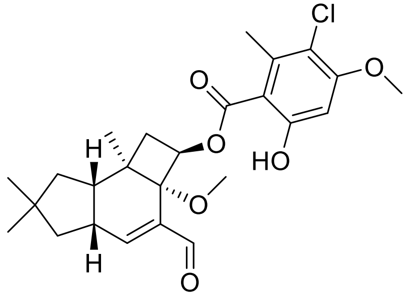	*Armillaria mellea*, **1990** [82]	Cytotoxicity against MCF7 (IC_{50} 6.7±1.1 μM), Jurkat (IC_{50} 4.1±0.8 μM), HeLa (IC_{50} 18.8±2.9 μM) and K-562 (IC_{50} 20.6±2.2 μM) [59].	**1990**- Relative by biogenetic consideration with 4-*O*-methylmelleolide [82].	
5′-Methoxy-armillasin (**124**)	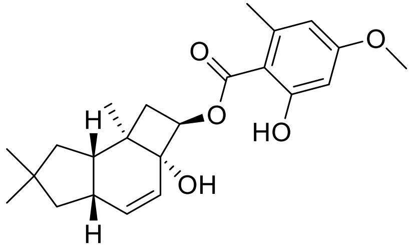	*Armillaria mellea*, **2016** [61]		**2016**- Relative by NOESY [61].	
5-Hydroxyl-armillarivin (**125**)	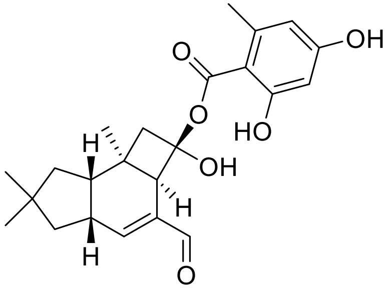	*Armillaria mellea*, **2016** [61]	Cytotoxicity against HepG2 (IC_{50} 18.03±6.03 μM) and L02 (IC_{50} 22.70±1.42 μM) [61].	**2016**- Relative by NOESY [61].	

Armellide A (**126**)	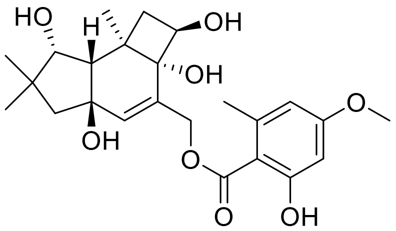	*Armillaria novae-zelandiae*, **1988** [65]		**1998**- Relative by NOESY [65].	
Armellide B (**127**)	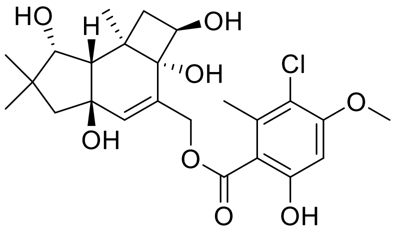	*Armillaria novae-zelandiae*, **1988** [65]	Cytotoxicity against MCF7 (IC_{50} >100 μM), Jurkat (IC_{50} >100 μM), HeLa (IC_{50} >100 μM) and K-562 (IC_{50} >100 μM) [59].	**1998**- Relative by NOESY [65].	
13-Deoxyarmellide A (**128**)	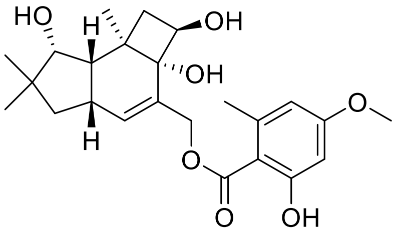	*Armillaria mellea*, **2014** [57]	Cytotoxicity against HUVEC (GI_{50} >115 μM), K-562 (GI_{50} >115 μM), MCF-7 (GI_{50} >115 μM) and HeLa (CC_{50} >115 μM) [57].	**2014**- Relative established by biogenetic consideration with melleolide I [57].	
13-Deoxyarmellide B (**129**)	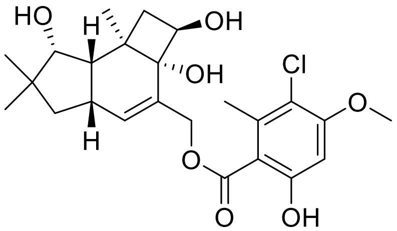	*Armillaria mellea*, **2014** [57]	Cytotoxicity against HUVEC (GI_{50} 91.0±2.0 μM), K-562 (GI_{50} 91.8±1.4 μM), MCF-7 (GI_{50} 90.8±0.9 μM) and HeLa (CC_{50} 89.5±0.5 μM) [57].	**2014**- Relative established by biogenetic consideration with melleolide I [57].	
5β,10α-Dihydroxy-1-orsellinate-dihydromelleolide (**130**)	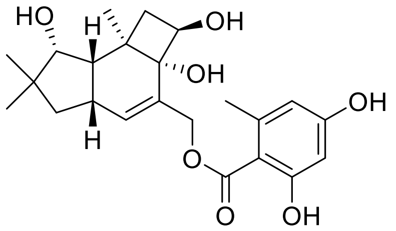	*Armillaria tabescens*, **1997** [69]		**1997**- Relative by NOESY [69].	

4-Dehydro-14-hydroxydihydromelleolide (**131**)	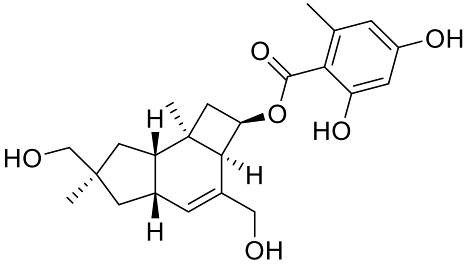	*Armillaria tabescens*, **1997** [69]		**1997**- Relative by NOESY [69].	
4-Dehydrodihydromelleolide (**132**)	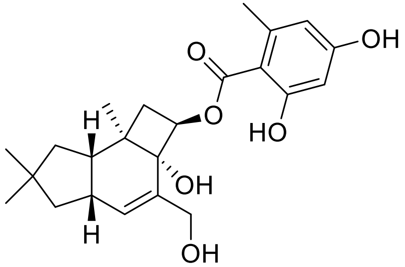	*Armillaria tabescens*, **1997** [69]		**1997**- Relative by NOESY [69].	
14-Hydroxydihydromelleolide (**133**)	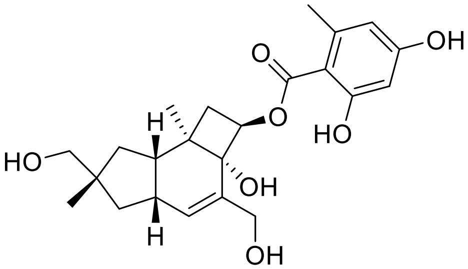	*Armillaria tabescens*, **1997** [69]		**1997**- Relative by NOESY [69].	
10-Hydroxydihydromelleolide (**134**)	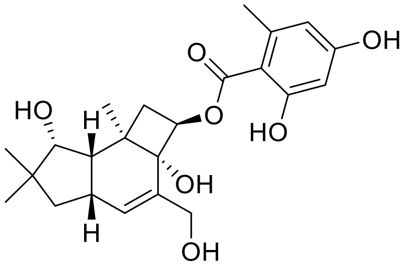	*Armillaria* sp., **1995** [83]			

A52a (**135**)	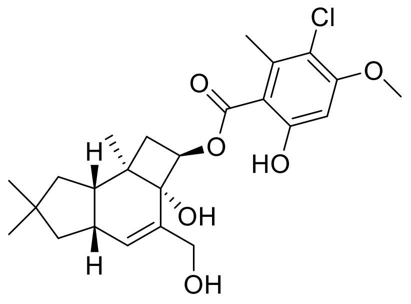	*Armillaria* sp., **1997** [74]	No antibiotic activity against *H. annosum*, *G. abietinum*, *E. coli* and *S. aureus* [74]. Cytotoxicity against Jurkat (IC_{50} 10.4±0.2 μM), HeLa (IC_{50} 40.0±1.1 μM), K-562 (IC_{50} 38.9±0.7 μM), HL-60 (IC_{50} 17.06 μM), SMMC-7721 (IC_{50} 17.77 μM), A-549 (IC_{50} 15.89 μM), MCF-7 (IC_{50} 14.10 μM) and SW480 (IC_{50} 15.70 μM) [59,63].		
A52b/ 10-Dehydroxy-melleolide B (**136**)	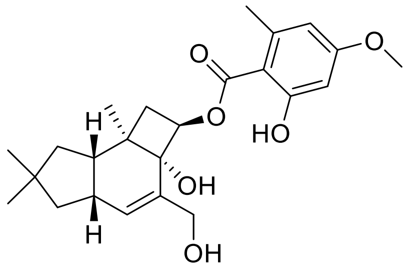	*Armillaria* sp., **1997** [74]	No antibiotic activity against *H. annosum*, *G. abietinum*, *E. coli* and *S. aureus* [74]. Cytotoxicity against HL-60 (IC_{50} 17.79 μM), SMMC-7721 (IC_{50} 20.90 μM), A-549 (IC_{50} 16.79 μM), MCF-7 (IC_{50} 16.49 μM) and SW480 (IC_{50} 17.44 μM) [63].	**2012**- Relative by NOESY [63].	
1-*O*-Formyl-10-dehydroxy-melleolide B (**137**)	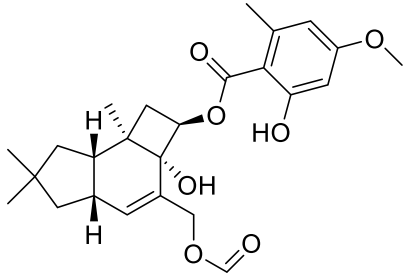	*Armillaria* sp., **2012** [63]	Cytotoxicity against HL-60 (IC_{50} 14.50 μM), SMMC-7721 (IC_{50} 23.16 μM), A-549 (IC_{50} 18.41 μM), MCF-7 (IC_{50} 5.34 μM) and SW480 (IC_{50} 10.77 μM) [63].	**2012**- Relative by biogenetic consideration with10-dehydroxy-melleolide B [63].	

10-Oxo-melleolide B (**138**)	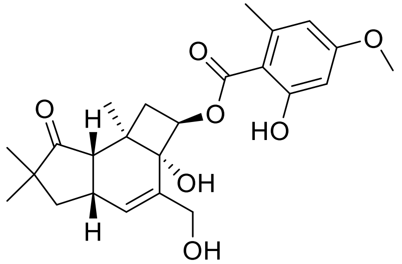	*Armillaria* sp., **2012** [63]	Cytotoxicity against HL-60 (IC_{50} >40 μM), SMMC-7721 (IC_{50} >40 μM), A-549 (IC_{50} >40 μM), MCF-7 (IC_{50} >40 μM) and SW480 (IC_{50} >40 μM) [63].	**2012**- Relative by biogenetic consideration with10-dehydroxy-melleolide B [63].	
10-Dehydroxymelleolide D (**139**)	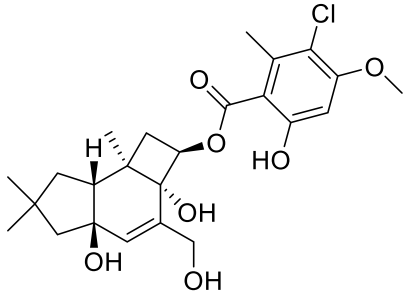	*Armillaria* sp., **2015** [84]		**2015-** Relative by biogenetic consideration with melleolide D. Absolute established by ECD [84].	
Armilliphatic A (**140**)	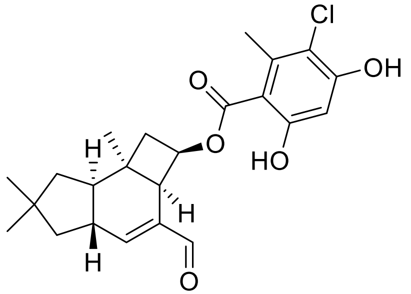	*Armillaria* sp. *and Epicoccum* sp., **2020** [36]	Moderate cytotoxicity against HL-60 (IC_{50} 15.8±0.32 μM), A-549 (IC_{50} 15.93±0.21 μM), SMMC-7721 (IC_{50} 19.42±0.81 μM), MCF-7 (IC_{50} 19.22±0.69 μM), SW480 (IC_{50} 23.03±0.44 μM). Weak acetylcholinesterase inhibitory activity (IC_{50} 23.85±0.20 μM) [36].	**2020-** Relative by ROESY. Absolute by ECD [36].	

13,14-Dihydroxy-A52a (**141**)	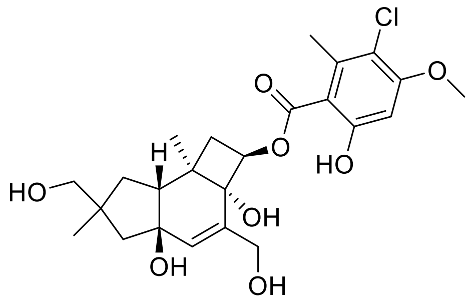	*Armillaria mellea*, **2011** [59]	Cytotoxicity against MCF7 (IC_{50} >100 μM), Jurkat (IC_{50} >100 μM), HeLa (IC_{50} >100 μM) and K-562 (IC_{50} >100 μM) [59].		
Armillane / Armillarizin (**142**)	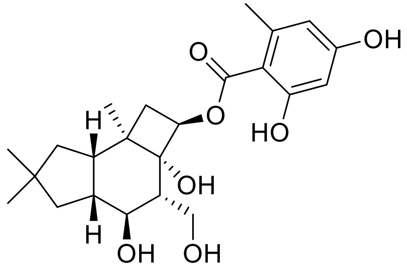	*Armillaria mellea*, **1990** [82]		**1990**- Relative established by NOESY [82].	
5′-Methoxy-6′-chloroarmillane (**143**)	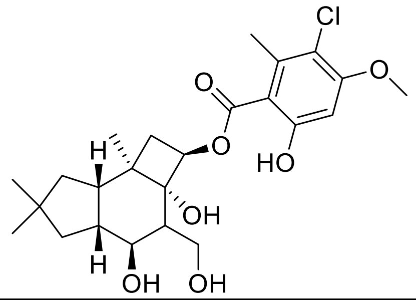	*Armillaria mellea*, **2011** [59]	Cytotoxicity against MCF7 (IC_{50} 26.5±0.4 μM), Jurkat (IC_{50} 13.3±0.5 μM), HeLa (IC_{50} 35.8±2.6 μM) and K-562 (IC_{50} 25.0±0.9 μM) [59].		
10-Hydroxy-5′-Methoxy-6′-chloroarmillane (**144**)	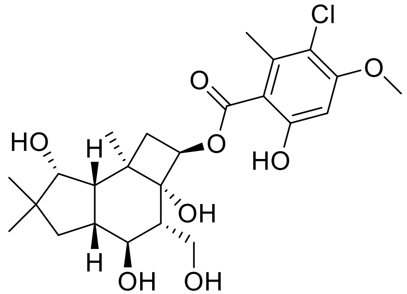	*Armillaria mellea*, **2014** [57]	Cytotoxicity against HUVEC (GI_{50} 58.7±1.8 μM), K-562 (GI_{50} 46.3±1.3 μM), MCF-7 (GI_{50} 64.7±1.2 μM) and HeLa (CC_{50} 76.2±1.1 μM) [57].	**2014**- Relative established by biogenetic consideration with 5′-methoxy-6′-chloroarmillane [57].	

Melleolide Q (**145**)	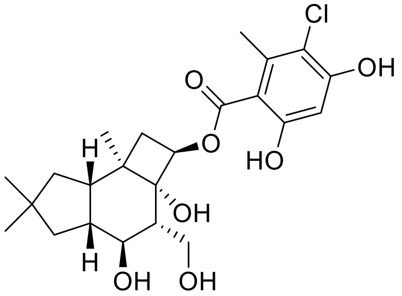	*Armillaria mellea*, **2015** [55]	Cytotoxicity against MCF7 (IC_{50} 1.5±0.1 μM), H460 (IC_{50} 80.0±8.9 μM), HT-29 (IC_{50} 54.2±4.7 μM) and CEM (IC_{50} 10.3±2.3 μM) [55].	**2015**- Relative by NOESY [55].	
Melleolide R (**146**)	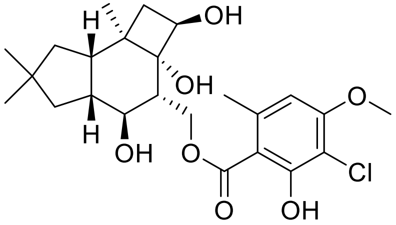	*Armillaria mellea*, **2015** [55]	Cytotoxicity against MCF7 (IC_{50} 3.7±0.3 μM), H460 (IC_{50} 53.8±6.2 μM), HT-29 (IC_{50} 18.7±3.2 μM) and CEM (IC_{50} 3.4±0.2 μM) [55].	**2015**- Relative by NOESY [55].	
Melleolide S (**147**)	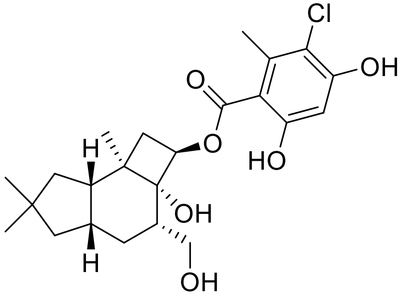	*Armillaria mellea*, **2011** [67]			
**Ascomycota**					
Punctaporonin A (**148**)	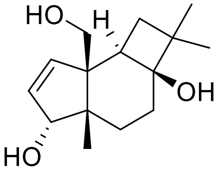	*Poronia punctata*, **1984** [85]		**1984-** X-ray [85] **1986-** Absolute established by synthesis [86]	**1986** – enantiospecific [86]

Punctaporonin D (**149**)	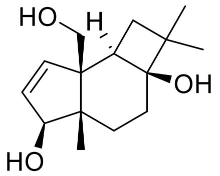	*Poronia punctata*, **1986** [87]	Inhibits a mycelial form of *C. albicans* at 1ppm [87].	**1986**- X-ray [87] **1986**- Absolute established by synthesis [86]	**1986 –** enantiospecific [86]
Punctaporonin E (**150**)	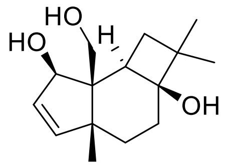	*Poronia punctata*, **1986** [87]	Inhibits *T. vaginalis* at 100 ppm *in vitro* [87].	**1986**- X-ray of punctatin E acetonide [87]	
Punctaporonin F (**151**)	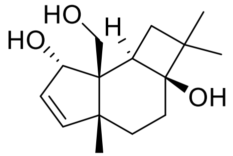	*Poronia punctata*, **1986** [87]			
6-Hydroxypunctaporonin A (**152**)	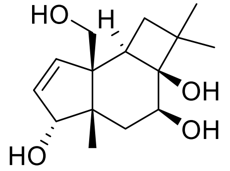	*Pestalotiopsis disseminate*, **2006** [88]	No activity against *B. subtilis* (ATCC6051), *S. aureus* (ATCC29213), *E.coli* (ATCC25922) and *C. albicans* (ATCC14053) [88]. No cytotoxicity against HeLa cells (IC_{50} >100 μM) [89].	**2006**- Relative by NOESY. Absolute established by biogenetic consideration with 6-hydroxypunctaporonin E [88].	
6-Hydroxypunctaporonin E (**153**)	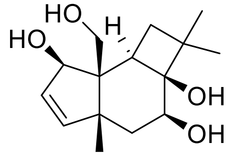	*Pestalotiopsis disseminate*, **2006** [88]	Antibacterial activity against *B. subtilis* (ATCC6051) 100 μg and *S. aureus* (ATCC29213) 100 μg. No activity against *E. coli* (ATCC25922) and *C. albicans* (ATCC14053) [88]. No cytotoxicity against HeLa cells (IC_{50} >100 μM) [89].	**2006**- Relative by NOESY. Absolute established by X-ray of mono-bromobenzoate derivative [88].	

Punctaporonin L (**154**)	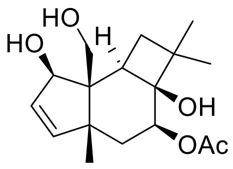	*Hansfordia sinuosae*, **2014** [90]	Weak cytotoxicity (IC_{50} >10 μM) against HCT-8, Bel7402, BGC823, A549, A2780 [90]. Weak antibacterial activity (MIC >125 μM) against *E. coli*, *S. aureus*, *B. thuringensis* and *B. subtilis* [90].	**2014-** Relative by NOESY. Absolute by biogenetic consideration with 6-hydroxypunctaporonin E [90].	
Punctaporonin M (**155**)	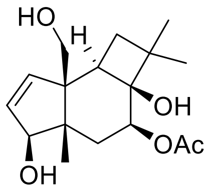	*Hansfordia sinuosae*, **2014** [90]	Weak cytotoxicity (IC_{50} >10 μM) against HCT-8, Bel7402, BGC823, A549, A2780 [90]. Weak antibacterial activity (MIC >125 μM) against *E. coli*, *S. aureus*, *B. thuringensis* and *B. subtilis* [90].	**2014-** Relative by NOESY [90].	
Punctaporonin N (**156**)	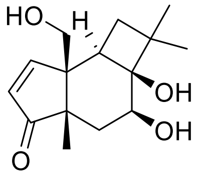	*Pestalotiopsis* sp., **2016** [91]	No antifungal activity against *A. flavus* and *F. verticillioides* and no antimicrobial activity against *S. aureus*, *B. subtilis*, *E. coli* and *C. albicans* on disks at 100 μg [91].	**2016-** Absolute by oxidation of 6-hydroxypunctaporonin A and biogenetic consideration [91].	
Punctaporonin O (**157**)	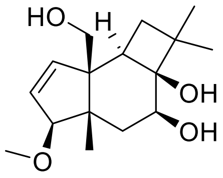	*Pestalotiopsis* sp., **2016** [91]	No antifungal activity against *A. flavus* and *F. verticillioides* and no antimicrobial activity against *S. aureus*, *B. subtilis*, *E. coli* and *C. albicans* on disks at 100 μg [91].	**2016-** Relative by NOESY. Absolute by biogenetic consideration with 6-hydroxypunctaporonin D [91].	

6-Hydroxypunctaporonin D (**158**)	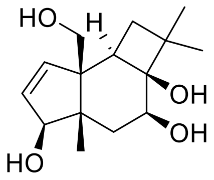	*Pestalotiopsis* sp., **2016** [91]	No antifungal activity against *A. flavus* and *F. verticillioides* and no antimicrobial activity against *S. aureus*, *B. subtilis*, *E. coli* and *C. albicans* on disks at 100 µg [91].	**2016-** Relative by NOESY. Absolute by biogenetic consideration with 6-hydroxypunctaporonin A [91].	
6,13-Dihydroxypunctaporonin A (**159**)	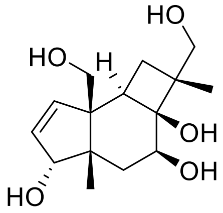	*Pestalotiopsis* sp., **2016** [91]	No antifungal activity against *A. flavus* and *F. verticillioides* and no antimicrobial activity against *S. aureus*, *B. subtilis*, *E. coli* and *C. albicans* on disks at 100 µg [91].	**2016-** Relative by NOESY. Absolute by biogenetic consideration with 6-hydroxypunctaporonin A [91].	
6,13-Dihydroxypunctaporonin E (**160**)	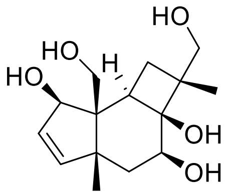	*Pestalotiopsis* sp., **2016** [91]	No antifungal activity against *A. flavus* and *F. verticillioides* and no antimicrobial activity against *S. aureus*, *B. subtilis*, *E. coli* and *C. albicans* on disks at 100 µg [91].	**2016-** Relative by NOESY. Absolute by biogenetic consideration with 6-hydroxypunctaporonin E [91].	
Punctaporonin O (**161**)	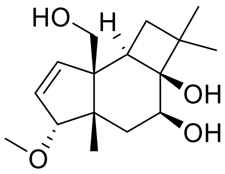	*Cytospora* sp., **2017** [89]	Moderate cytotoxicity against HeLa cells (IC_{50} 16.6 µM) [89].	**2017**- Absolute by biogenetic consideration with 6-hydroxypunctaporonin A [89].	

Punctaporonin P (**162**)	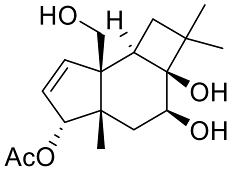	*Cytospora* sp., **2017** [89]	No cytotoxicity against HeLa cells (IC_{50} >100 μM) [89].	**2017**- Absolute by biogenetic consideration with 6-hydroxypunctaporonin A [89].	
Punctaporonin Q (**163**)	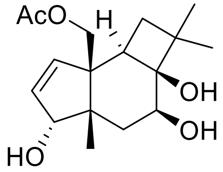	*Cytospora* sp., **2017** [89]	No cytotoxicity against HeLa cells (IC_{50} >100 μM) [89].	**2017**- Absolute by biogenetic consideration with 6-hydroxypunctaporonin A [89].	
Punctaporonin R (**164**)	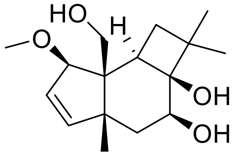	*Cytospora* sp., **2017** [89]	Moderate cytotoxicity against HeLa cells (IC_{50} 10.4 μM) [89].	**2017**- Absolute by biogenetic consideration with 6-hydroxypunctaporonin E [89].	
Punctaporonin S (**165**)	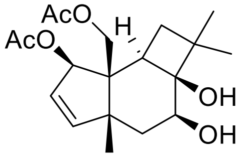	*Cytospora* sp., **2017** [89]	Weak cytotoxicity against HeLa cells (IC_{50} 47.4 μM) [89].	**2017**- Absolute by biogenetic consideration with 6-hydroxypunctaporonin E [89].	
Phomophyllin B (**166**)	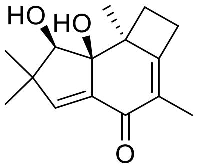	*Phomopsis* sp. TJ507A, **2018** [92]	Inhibitor of BACE1 (target for Alzheimer disease) (~35% inhibition at 40 μM).<br>No hepatoxicity to L-02 liver cells at 40 μM [92].	**2018**- Relative by NOESY. Absolute established by X-ray [92].	

Phomophyllin C (**167**)	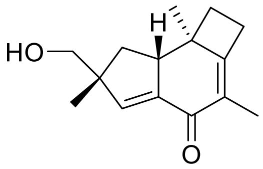	*Phomopsis* sp. TJ507A, **2018** [92]	Inhibitor of BACE1 (target for Alzheimer disease) (~20% inhibition at 40 μM). No hepatoxicity to L-02 liver cells at 40 μM [92].	**2018**- Relative by NOESY. Absolute established by ECD [92].	
Phomophyllin D (**168**)	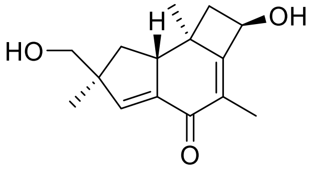	*Phomopsis* sp. TJ507A, **2018** [92]	Inhibitor of BACE1 (target for Alzheimer disease) (~40% inhibition at 40 μM). No hepatoxicity to L-02 liver cells at 40 μM [92].	**2018**- Relative by NOESY. Absolute established by ECD [92].	
Phomophyllin E (**169**)	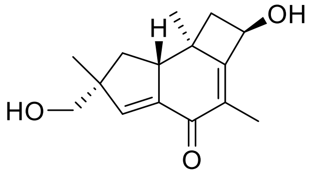	*Phomopsis* sp. TJ507A, **2018** [92]	Inhibitor of BACE1 (target for Alzheimer disease) (~37% inhibition at 40 μM). No hepatoxicity to L-02 liver cells at 40 μM [92].	**2018**- Relative by NOESY. Absolute established by ECD [92].	
Phomophyllin F (**170**)	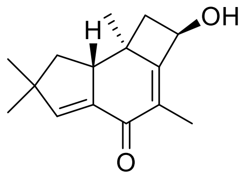	*Phomopsis* sp. TJ507A, **2018** [92]	Inhibitor of BACE1 (target for Alzheimer disease) (~38% inhibition at 40 μM). No hepatoxicity to L-02 liver cells at 40 μM [92].	**2018**- Relative by NOESY. Absolute established by modified Mosher's method (MTPA) [92].	
Phomophyllin G (**171**)	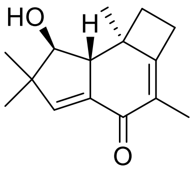	*Phomopsis* sp. TJ507A, **2018** [92]	Inhibitor of BACE1 (target for Alzheimer disease) (~21% inhibition at 40 μM). No hepatoxicity to L-02 liver cells at 40 μM [92].	**2018**- Relative by NOESY. Absolute established by modified Mosher's method (MTPA) [92].	

Phomophyllin H (**172**)	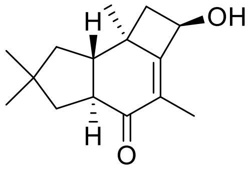	*Phomopsis* sp. TJ507A, **2018** [92]		**2018**- Relative by NOESY. Absolute established by ECD [92].	
Phomophyllin I (**173**)	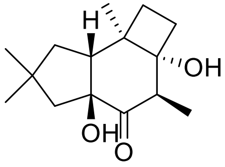	*Phomopsis* sp. TJ507A, **2018** [92]	Inhibitor of BACE1 (target for Alzheimer disease) (~39% inhibition at 40 μM).<br>No hepatoxicity to L-02 liver cells at 40 μM [92].	**2018**- Absolute established by X-ray [92].	
**Marine**					
Paesslerin A (**174**)	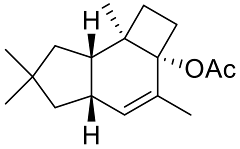	*Alcyonium paessleri*, **2001** [93]	Moderate cytotoxicity in preliminary studies [93].	**2001**- Relative by NOESY [93].	**2019**- Total synthesis and structure correction [94]
Paesslerin B (**175**)	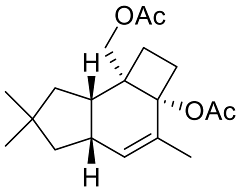	*Alcyonium paessleri*, **2001** [93]	Moderate cytotoxicity in preliminary studies [93].	**2001**- Relative by NOESY [93].	
**Plants**					
Pteridanoside (**176**)	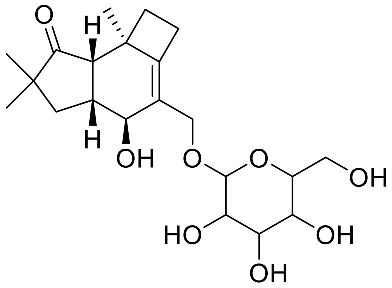	*Pteridium aquilinum* var. *caudatum*, **1999** [95]	Toxicity against brine shrimp *Artemia salina* (LC_{50} = 250 and 62.5 μg/mL, 24h and 48h) [95].	**1999**- Relative established by NOESY. Absolute by biogenetic consideration with pteridanone [95].	

Pteridanone (**177**)	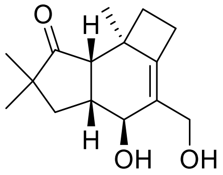	*Pteridium aquilinum* var. *caudatum*, **1999** [95]	No toxicity against brine shrimp *Artemia salina* [95].	**1999**- Relative established by NOESY. Absolute by ECD and conformational calculations [95].	
Xanthocerapene (**178**)	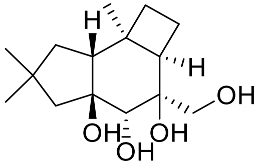	*Xanthoceras sorbifolia*, **2004** [96]		**2004**- Relative by NOESY [96].	
2,2,4a,7a-Tetramethyldecahydro-1*H*-cyclobuta[*e*]inden-5-ol (**179**)	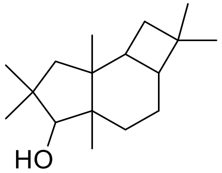	*Radix et Rhizoma ginseng*, **2011** [97]		**2011**- Structure by GC-MS [97].	
(3S,4aS,6R,8aS,8bR)-6-Hydroxy-3,6,8a-trimethyloctahydro-3,8b-methanocyclobuta[h]chromene-5,9(6H)-dione (**180**)	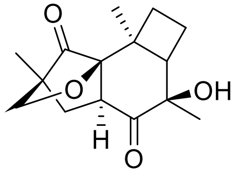	*Lindera strychnifolia*, **2016** [98]	Improve the cell viability of human umbilical vein endothelial cells injured by ox-LDL [98].	**2016-** Relative by NOESY. Absolute by ECD [98].	

References

1. Anchel, M.; Hervey, A.; Robbins, W.J. *PNAS* **1950**, *36*, 300–305.
2. McMorris, T.C.; Nair, M.S.R.; Anchel, M. *J. Am. Chem. Soc.* **1967**, *89*, 4562–4563.
3. McMorris, T.C.; Nair, M.S.R.; Singh, P.; Anchel, M. *Phytochemistry* **1971**, *10*, 1611.
4. Matsumoto, T.; Miyano, K.; Kagawa, S.; Yi, S.; Ogawa, J.; Ichihara, A. *Tet. Lett.* **1971**, *38*, 3521–3524.
5. Nair, M.S.R.; Anchel, M. *Tet. Lett.* **1975**, *14*, 1267–1268.
6. Suzuki, S.; Murayama, T.; Shiono, Y. *Z. Naturforsch.* **2006**, *61b*, 1295–1298.
7. Arnone, A.; Cardillo, R.; Modugno, V.D.; Nasini, G. *J. Chem. Soc. Perkin Trans. 1* **1989**, 1995–2000.

8. Suzuki, S.; Murayama, T.; Shiono, Y. *Phytochemistry* **2005**, *66*, 2329–2333.
9. Assante, G.; Dallavalle, S.; Martino, P.A. *J Antibiot* **2013**, *66*, 43–45.
10. Elliott, M.R.; Dhimane, A.-L.; Malacria, M. *J. Am. Chem. Soc.* **1997**, *119*, 3427–3428.
11. McMorris, T.C.; Kashinatham, A.; Lira, R.; Rundgren, H.; Gantzel, P.K.; Kelner, M.J.; Dawe, R. *Phytochemistry* **2002**, *61*, 395–398.
12. Yang, X.-Y.; Li, Z.-H.; Dong, Z.-J.; Feng, T.; Liu, J.-K. *J. Asian Nat. Prod. Res.* **2015**, *17*, 1–6.
13. Nozoe, S.; Kobayashi, H.; Urano, S.; Furukawa, J. *Tet. Lett.* **1977**, 1381–1384.
14. Furukawa, J.; Morisaki, N.; Kobayashi, H.; Iwasaki, S.; Nozoe, S.; Okuda, S. *Chem. Pharm. Bull.* **1985**, *33*, 440–443.
15. Rabe, P.; Rinkel, J.; Nubbemeyer, B.; Köllner, T.G.; Chen, F.; Dickschat, J.S. *Angew. Chem. Int. Ed.* **2016**, *55*, 15420–15423.
16. Takeshita, H.; Iwabuchi, H.; Kouno, I.; Iino, M.; Nomura, D. *Chem. Lett.* **1979**, *8*, 649–652.
17. Sasaki, H.; Kubohara, Y.; Ishigaki, H.; Takahashi, K.; Eguchi, H.; Sugawara, A.; Oshima, Y.; Kikuchi, H. *Molecules* **2020**, *25*, 2895.
18. Arnone, A.; Nasini, G.; Assante, G.; Roeijmans, H.J.; Van Euk, G.W. *Phytochemistry* **1987**, *26*, 1739–1742.
19. Arnone, A.; Nasini, G.; Assante, G.; Eijk, G.W.V. *Phytochemistry* **1992**, *31*, 2047–2050.
20. Arnone, A.; Brambilla, U.; Nasini, G.; de Pava, O.V. *Tetrahedron* **1995**, *51*, 13357–13364.
21. Arnone, A.; De Gregorio, C.; Meille, S.V.; Nasini, G.; Sidoti, G. *J. Nat. Prod.* **1999**, *62*, 51–53.
22. Zheng, Y.; Shen, Y. *Org. Lett.* **2009**, *11*, 109–112.
23. Zheng, Y.-B.; Lu, C.-H.; Zheng, Z.-H.; Lin, X.-J.; Su, W.-J.; Shen, Y.-M. *Helv. Chim. Acta.* **2008**, *91*, 2174–2180.
24. Clericuzio, M.; Fu, J.; Pan, F.; Pang, Z.; Sterner, O. *Tetrahedron* **1997**, *53*, 9735–9740.
25. Clericuzio, M.; Han, F.; Pan, F.; Pang, Z.; Sterner, O. *Acta Chem. Scand.* **1998**, *52*, 1333–1337.
26. Clericuzio, M.; Pan, F.; Han, F.; Pang, Z.; Sterner, O. *Tet. Lett.* **1997**, *38*, 8237–8240.
27. Yoshikawa, K.; Kaneko, A.; Matsumoto, Y.; Hama, H.; Arihara, S. *J. Nat. Prod.* **2006**, *69*, 1267–1270.
28. Clericuzio, M.; Mella, M.; Toma, L.; Finzi, P.V.; Vidari, G. *Eur. J. Org. Chem.* **2002**, *6*, 988–994.
29. Zech, A.; Jandl, C.; Bach, T. *Angew. Chem. Int. Ed.* **2019**, *58*, 14629–14632.
30. Proessdorf, J.; Zech, A.; Jandl, C.; Bach, T. *Synlett* **2020**, *31*, 1598–1602.
31. Hirota, M.; Shimizu, Y.; Kamo, T.; Makabe, H.; Shibata, H. *Biosci. Biotechnol. Biochem.* **2003**, *67*, 1597–1600.
32. Takeuchi, T.; Iinuma, H.; Momose, I.; Matsui, S. Antibiotic pasteurestin A and B and their manufacture with *Agrocybe cylindracea*. **2002**, *Jpn. Kokai Tokkyo Koho*, JP 2002212137.
33. Kögl, M.; Brecker, L.; Warrass, R.; Mulzer, J. *Angew. Chem. Int. Ed.* **2007**, *46*, 9320–9322
34. Kögl, M.; Brecker, L.; Warrass, R.; Mulzer, J. *Eur. J. Org. Chem.* **2008**, 2714–2730.
35. Surup, F.; Hennicke, F.; Sella, N.; Stroot, M.; Bernecker, S.; Pfütze, S.; Stadler, M.; Rühl, M. *Beilstein J. Org. Chem.* **2019**, *15*, 1000–1007.
36. Li, H.-T.; Tang, L.-H.; Liu, T.; Yang, R.-N.; Yang, Y.-B.; Zhou, H.; Ding, Z.-T. *Bioorg. Chem.* **2020**, *95*, 103503.
37. Stärk, A.; Anke, T.; Mocek, U.; Steglich, W.; Kirfel, A.; Will, G. *Z. Naturforsch. C* **1988**, *43*, 177–183.
38. Wunder, A.; Anke, T.; Klostermeyer, D.; Steglich, W. *Z. Naturforsch. C* **1996**, *51*, 493–499.

39. Fabian, K.; Lorenzen, K.; Anke, T.; Johansson, M.; Sterner, O. *Z. Naturforsch. C* **1998**, *53*, 939–945.
40. Chang, E.L.; Bolte, B.; Lan, P.; Willis, A.C.; Banwell, M.G. *J. Org. Chem.* **2016**, *81*, 2078–2086.
41. Starratt, A.N.; Stothers, J.B.; Ward, E.W.B. *J. Chem. Soc., Chem. Commun.* **1988**, 590–591.
42. Starratt, A.N.; Ward, E.W.B.; Stothers, J.B. *Can. J. Chem.* **1989**, *67*, 417–427.
43. Nord, C.L.; Menkis, A.; Vasaitis, R.; Broberg, A. *Phytochemistry* **2013**, *90*, 128–134.
44. Nord, C.L.; Menkis, A.; Lendel, C.; Vasaitis, R.; Broberg, *Phytochemistry* **2014**, *102*, 197–204.
45. Shiono, Y.; Seto, T.; Kamata, M.; Takita, C.; Suzuki, S.; Murayama, T.; Ikeda, M. *Z. Naturforsch.* **2004**, *59b*, 925–929.
46. Shimoda, K.; Yamaoka, Y.; Yoo, D.; Yamada, K.; Takikawa, H.; Takasu, K. *J. Org. Chem.* **2019**, *84*, 11014–11024.
47. Shiono, Y.; Suzuki, S.; Murayama, T.; Ikeda, M.; Abe, Y.; Sassa, T. *Z. Naturforsch. B* **2005**, *60*, 449–452.
48. Donnelly, D.; Sanada, S.; O'Reilly, J.; Polonsky, J.; Prangé, T.; Pascard, C. *J. Chem. Soc., Chem. Commun.* **1982**, 135–137.
49. Cremin, P.; Guiry, P.J.; Wolfender, J.-L.; Hostettmann, K.; Donnelly, D.M.X. *J. Chem. Soc., Perkin Trans. 1* **2000**, 2325–2329.
50. Donnelly, D.M.X.; Polonsky, J.; Prangé, T.; Snatzke, G.; Wagner, U. *J. Chem. Soc., Chem. Commun.* **1984**, 222–223.
51. Donnelly, D.M.X.; Abe, F.; Coveney, D.; Fukuda, N.; O'Reilly, J.; Polonsky, J.; Prangé, T. *J. Nat. Prod.* **1985**, *48*, 10–16.
52. Donnelly, D.M.X.; Coveney, D.J.; Fukuda, N.; Polonsky, J. *J. Nat. Prod.* **1986**, *49*, 111–116.
53. Yang, J.-S.; Cong, P.-Z. *Huaxue Xuebao* **1988**, *46*, 1093–1100.
54. Yang, J.S.; Chen, Y.W.; Feng, X.Z.; Yu, D.Q.; He, C.H.; Zheng, Q.T.; Yang, J.; Liang, X.T. *Planta Med* **1989**, *55*, 564–565.
55. Chen, C.-C.; Kuo, Y.-H.; Cheng, J.-J.; Sung, P.-J.; Ni, C.-L.; Chen, C.-C.; Shen, C.-C. *Molecules* **2015**, *20*, 9994–10003.
56. Misiek, M.; Williams, J.; Schmich, K.; Hüttel, W.; Merfort, I.; Salomon, C.E.; Aldrich, C.C.; Hoffmeister, D. *J. Nat. Prod.* **2009**, *72*, 1888–1891.
57. Bohnert, M.; Nützmann, H.-W.; Schroeckh, V.; Horn, F.; Dahse, H.-M.; Brakhage, A.A.; Hoffmeister, D. *Phytochemistry* **2014**, *105*, 101–108.
58. Misiek, M.; Hoffmeister, D. *Mycol. Progress* **2012**, *11*, 7–15.
59. Bohnert, M.; Miethbauer, S.; Dahse, H.-M.; Ziemen, J.; Nett, M.; Hoffmeister, D. *Bioorg. Med. Chem. Lett.* **2011**, *21*, 2003–2006.
60. Midland, S.L.; Izac, R.R.; Wing, R.M.; Zaki, A.I.; Munnecke, D.E.; Sims, J.J. *Tet. Lett.* **1982**, *23*, 2515–2518.
61. Li, Z.; Wang, Y.; Jiang, B.; Li, W.; Zheng, L.; Yang, X.; Bao, Y.; Sun, L.; Huang, Y.; Li, Y. *J. Ethnopharmacol.* **2016**, *184*, 119–127.
62. Arnone, A.; Cardillo, R.; Nasini, G. *Phytochemistry* **1986**, *25*, 471–474.
63. Yin, X.; Feng, T.; Liu, J.-K. *Nat. Prod. Bioprospect.* **2012**, *2*, 245–248.
64. Arnone, A.; Cardillo, R.; Di Modugno, V.; Nasini, G. *Gazz. Chim. Ital.* **1988**, *118*, 517–521.
65. Arnone, A.; Cardillo, R.; Nasini, G. *Gazz. Chim. Ital.* **1988**, *118*, 523–527.
66. Momose, I.; Sekizawa, R.; Hosokawa, N.; Iinuma, H.; Maisui, S.; Nakamura, H.; Naganawa, H.; Hamada, M.; Takeuchi, T. *J. Antibiot.* **2000**, *53*, 137–143.
67. Chen, C.-C.; Cheng, J.-J.; Shen, C.-C. Protoilludane norsesquiterpenoid esters and uses thereof. **2011**, US 20110262561.

68. Donnelly, D.M.X.; Hutchinson, R.M.; Coveney, D.; Yonemitsu, M. *Phytochemistry* **1990**, *29*, 2569–2572.
69. Donnelly, D.M.X.; Konishi, T.; Dunne, O.; Cremin, P. *Phytochemistry* **1997**, *44*, 1473–1478.
70. Donnelly, D.M.X.; Coveney, D.J.; Polonsky, J. *Tet. Lett.* **1985**, *26*, 5343–5344.
71. Arnone, A.; Cardillo, R.; Nasini, G.; Meille, S.V. *J. Chem. Soc., Perkin Trans. 1* **1988**, 503–510.
72. Donnelly, D.M.X.; Quigley, P.F.; Coveney, D.J.; Polonsky, J. *Phytochemistry* **1987**, *26*, 3075–3077.
73. Yang, J.S.; Chen, Y. W.; Feng, X. Z.; Yu, D. Q.; Liang, X. T. *Planta Med.* **1984**, *50*, 288–290.
74. Sonnenbichler, J.; Guillaumin, J.-J.; Peipp, H.; Schwarz, D. *Eur. J. For. Path.* **1997**, *27*, 241–249.
75. Yang, J.S.; Su, Y.L.; Wang, Y.L.; Feng, X.Z.; Yu, D.Q.; Cong, P.Z.; Tamai, M.; Obuchi, T.; Kondoh, H.; Liang, X.T. *Planta Med.* **1989**, *55*, 479–481.
76. Yang, J.S.; Su, Y.L.; Wang, Y.L.; Feng, X.Z.; Yu, D.Q.; Liang, X.T. *Yao Xue Xue Bao* **1990**, *25*, 24–28.
77. Yang, J.S.; Su, Y.L.; Wang, Y.L.; Feng, X.Z.; Yu, D.Q.; Liang, X.T.; He, C.H.; Zheng, Q.T.; Yang, J.J.; Yang, J. *Yao Xue Xue Bao* **1990**, *25*, 353–356.
78. Yang, J.S.; Su, Y.L.; Wang, Y.L.; Feng, X.Z.; Yu, D.Q.; Liang, X.T. *Yao Xue Xue Bao* **1991**, *26*, 117–122.
79. Yang, J.S.; Su, Y.L.; Wang, Y.L.; Feng, X.Z.; Yu, D.Q.; Liang, X.T. *Planta Med.* **1991**, *57*, 478–480.
80. Yang, J.S.; Watube, S. Antibiotic armillaric acid and its manufacture with *Armillaria mellea*. **1989**, CN 1034956.
81. Obuchi, T.; Kondoh, H.; Watanabe, N.; Tamai, M.; Omura, S.; Yang, J.S.; Liang, X.T. *Planta Med.* **1990**, *56*, 198–201.
82. Donnelly, D.M.X.; Hutchinson, R.M. *Phytochemistry* **1990**, *29*, 179–182.
83. Cremin, P.; Donnelly, D.M.X.; Wolfender, J.-L.; Hostettmann, K. *J. Chromatogr. A* **1995**, *710*, 273–285.
84. Kobori, H.; Sekiya, A.; Suzuki, T.; Choi, J.-H.; Hirai, H.; Kawagishi, H. *J. Nat. Prod.* **2015**, *78*, 163–167.
85. Anderson, J.R.; Briant, C.E.; Edwards, R.L.; Mabelis, R.P.; Poyser, J.P.; Spencer, H.; Whalley, A.J.S. *J. Chem. Soc. Chem. Commun.* **1984**, *7*, 405–406.
86. Sugimura, T.; Paquette, L.A. *J. Am. Chem. Soc.* **1987**, *109*, 3017–3024.
87. Poyser, J.P.; Edwards, R.L.; Anderson, J.R.; Hursthouse, M.B.; Walker, N.P.C.; Sheldrick, G.M.; Whalley, A.J.S. *J. Antibiot.* **1986**, *39*, 167–169.
88. Deyrup, S.T.; Swenson, D.C.; Gloer, J.B.; Wicklow, D.T. *J. Nat. Prod.* **2006**, *69*, 608–611.
89. Li, Y.; Wang, Q.; Liu, X.; Che, Y. *Biomed. Res. Int.* **2017**, 7871459.
90. Wu, Z.; Liu, D.; Proksch, P.; Guo, P.; Lin, W. *Mar. Drugs* **2014**, *12*, 3904–3916.
91. Hwang, I.H.; Wicklow, D.T.; Gloer, J.B. *Phytochem. Lett.* **2016**, *16*, 257–262.
92. Xie, S.; Wu, Y.; Qiao, Y.; Guo, Y.; Wang, J.; Hu, Z.; Zhang, Q.; Li, X.; Huang, J.; Zhou, Q.; Luo, Z.; Liu, J.; Zhu, H.; Xue, Y.; Zhang, Y. *J. Nat. Prod.* **2018**, *81*, 1311–1320.
93. Brasco, M.F.R.; Seldes, A.M.; Palermo, J.A. *Org. Lett.* **2001**, *3*, 1415–1417.
94. Mogi, Y.; Inanaga, K.; Tokuyama, H.; Ihara, M.; Yamaoka, Y.; Yamada, K.; Takasu, K. *Org. Lett.* **2019**, *21*, 3954–3958.

95. Castillo, U.F.; Sakagami, Y.; Alonso-Amelot, M.; Ojika, M. *Tetrahedron* **1999**, *55*, 12295–12300.
96. Ma, C.M.; Nakamura, N.; Nawawi, A.; Hattori, M.; Cai, S.Q. *Chinese Chem. Lett.* **2004**, *15*, 65–67.
97. Wang, J.; Zhang, L.-X.; Zhao, Y.; Chen, W.-X.; Yang, Q.; Wang, Y.-X. *Medicinal Plant* **2011**, *2*, 40–43, 51.
98. Wu, J. Protoilludane sesquiterpenoid compound, preparation method and medical applications thereof. **2016**, CN 105418544.
